# Characterization of a new murine cell line of sarcomatoid hepatocellular carcinoma and its application for biomarker/therapy development

**DOI:** 10.1038/s41598-017-03164-3

**Published:** 2017-06-08

**Authors:** Chia-Hung Yen, Chih-Chung Lai, Chen-Chung Liao, Sheng-Fan Wang, Yi-Jen Liao, Chien-Yi Tung, Jung-Hsien Hung, Shiu-Feng Huang, Yi-Ming Arthur Chen

**Affiliations:** 1 Graduate Institute of Natural Products, College of Pharmacy, Kaohsiung Medical University, Kaohsiung, Taiwan; 2 Center for Infectious Disease and Cancer Research, Kaohsiung Medical University, Kaohsiung, Taiwan; 3 Research Center for Natural products & Drug Development, Kaohsiung Medical University, Kaohsiung, Taiwan; 4 Department of Medical Laboratory Science and Biotechnology, Kaohsiung Medical University, Kaohsiung, Taiwan; 5 Department of Medical Research, Kaohsiung Medical University Hospital, Kaohsiung, Taiwan; 6 Proteomics Research Center, National Yang-Ming University, Taipei, Taiwan; 7 VYM Genome Research Center, National Yang-Ming University, Taipei, Taiwan; 8 School of Medical Laboratory Science and Biotechnology, College of Medical Science and Technology, Taipei Medical University, Taipei, Taiwan; 9 Division of Molecular and Genomic Medicine, National Health Research Institutes, Miaoli, Taiwan; 10 Graduate Institute of Medicine, College of Medicine, Kaohsiung Medical University, Kaohsiung, Taiwan; 11 Institute of Biomedical Sciences, National Sun Yat-sen University, Kaohsiung, Taiwan

## Abstract

Sarcomatoid hepatocellular carcinoma (SHC) is a rare type of HCC with significantly poorer survival than ordinary HCC. Little is known about the mechanism associated with SHC and its biomarkers and therapy. Here, we established a mouse liver cancer cell line and designated as Ymac-1. A sarcomatous appearance was observed in the allograft tumor arose from Ymac-1. Liver-secreted plasma proteins were found in Ymac-1 cultured supernatant by proteomics analysis. The positive staining of CK7, CK8, Vimentin and the suppressed expression of AFP indicated that Ymac-1 is a SHC cell line. Compared to its original tumor, an elevated level of EMT markers, N-cadherin and Vimentin, was found in Ymac-1. Ymac-1 displayed a higher migration rate and side population percentage than a mouse ordinary HCC cell line-Hepa1-6. Microarray analysis was performed to identify potential biomarkers/therapeutic targets for SHC. G6pd, a vital enzyme in pentose phosphate pathway, is highly expressed in Ymac-1. Depletion of G6pd in Ymac-1 reduced CD133 expression and sphere formation. Positive correlations between G6PD and CD133 were observed in human specimen. Higher expression of both G6PD and CD133 in tumor were associated with poor survival. In summary Ymac-1 can be a useful SHC cell model for novel biomarker and therapy development.

## Introduction

Sarcomatoid dedifferentiation of cancer cells (carcinomas with spindle-cell components) is one of the interesting histopathologic features of carcinomas^1, 2^. Sarcomatoid changes of carcinoma can be observed in many organs, including the kidney, bladder, prostate, lung, skin, thyroid, Gastrointestinal tract and liver^1, 3–5^. The incidence of sarcomatoid hepatocellular carcinoma (SHC) is quite low with ~2% in surgically resected cases and ~10% in autopsied cases^5, 6^. Although SHC is a very rare histologic variant of hepatocellular carcinoma (HCC), the prognosis of patients with the SHC was significantly worse than ordinary HCC cases^5, 7^. The poor prognosis has been attributed to the highly metastatic property of sarcomatous cells^8, 9^. In addition, SHC has been reported to be relatively resistant to transarterial (chemo) embolization (TAE/TACE) therapy, thus tumor recurs early after treatment^9, 10^. Interestingly, more than 20% of the cases who received anticancer treatment showed sarcomatoid changes, while a sarcomatous appearance was found in only 4.2% of the cases without anticancer treatment^11^. Together, SHC is a malignant liver tumor which possesses metastatic and chemotherapy resistant abilities.

It has been proposed that sarcomatoid cells in liver cancers are originated from trans-differentiation of HCC or cholangiocarcinoma^12, 13^. The activation of an epithelial–mesenchymal transition (EMT) program is proposed to play a crucial role in the trans-differentiation process from epithelial into sarcoma/sarcoma-like cells^1, 2, 14^. With regard to the histopathological characteristics, sarcomatoid elements of HCC showed positive staining for Vimentin. Cytokeratin 7 and 8 (CK7 and CK8) staining has been recommended for differentiating SHC from true sarcomas^8, 15–17^. In addition, unlike ordinary HCC that frequently expressed high level of α-fetoprotein (AFP), one special clinical features of SHC is characterized by the negative or low expression of AFP^16, 18^. However, due to the heterogeneity nature of liver cancer, it is difficult to distinguish SHC from ordinary HCC on imaging findings alone. SHC can only be detected in 1.8% of surgically resected cases, not even to mention detecting SHC form needle biopsy sample^18^. Therefore, identifying molecular markers for SHC early diagnosis are urgently needed. In addition, developing novel therapeutic modalities by targeting SHC population could also be benefit to future HCC management.

Glycine N-methyltransferase (GNMT) is a tumor suppressor gene for HCC^19, 20^. Two *Gnmt*−/− mouse models have been established and spontaneous HCC development was found in both *Gnmt*−/− mouse models^21, 22^. It has been reported that regeneration of the liver by hepatocyte is inhibited in *Gnmt*−/− mice^23^. Therefore, the normally dormant stem cells/progenitor cells in liver take the place of hepatocyte and proliferate and differentiate to replenish the liver parenchyma. Eventually, these stem cells/progenitor cells may undergo un-controlled proliferation and form liver tumors. Therefore, it is reasonable to hypothesize that liver tumor from *Gnmt*−/− mice should be composed of heterogeneous cell population containing stem cell/progenitor cell-like subpopulation. Previously, we established a cancer cell line from the liver tumor of *Gnmt*−/− mice and designated it as Ymac-1 cell. We successively subcultured the cell more than 100 passages. Herein, we showed that the cell morphology of Ymac-1 cell was not similar to typical epithelial type HCC cells. Surprisingly, a sarcomatous appearance was observed in the allograft tumor arising from Ymac-1 by both subcutaneously and orthotopic transplantation. The positive staining of CK8 and suppressed expression of AFP indicated Ymac-1 cell is a sarcomatoid HCC cell line. Furthermore, Ymac-1 cell expressed EMT markers and possessed cancer stem cell-like properties. Microarray profiling revealed the enrichment of glutathione pathway in Ymac-1 cell. Depletion of glucose-6-phosphate dehydrogenase (G6PD), the pivotal enzyme in pentose phosphate pathway and glutathione metabolism, reduced CD133 expression and sphere forming ability of Yamc-1 cell. Accordingly, Ymac-1 cell could be an excellent model for SHC research and may shed light on the prevention, diagnosis and treatment of SHC.

## Results

### Establishment of Ymac-1 cell line

**Figure 1:**
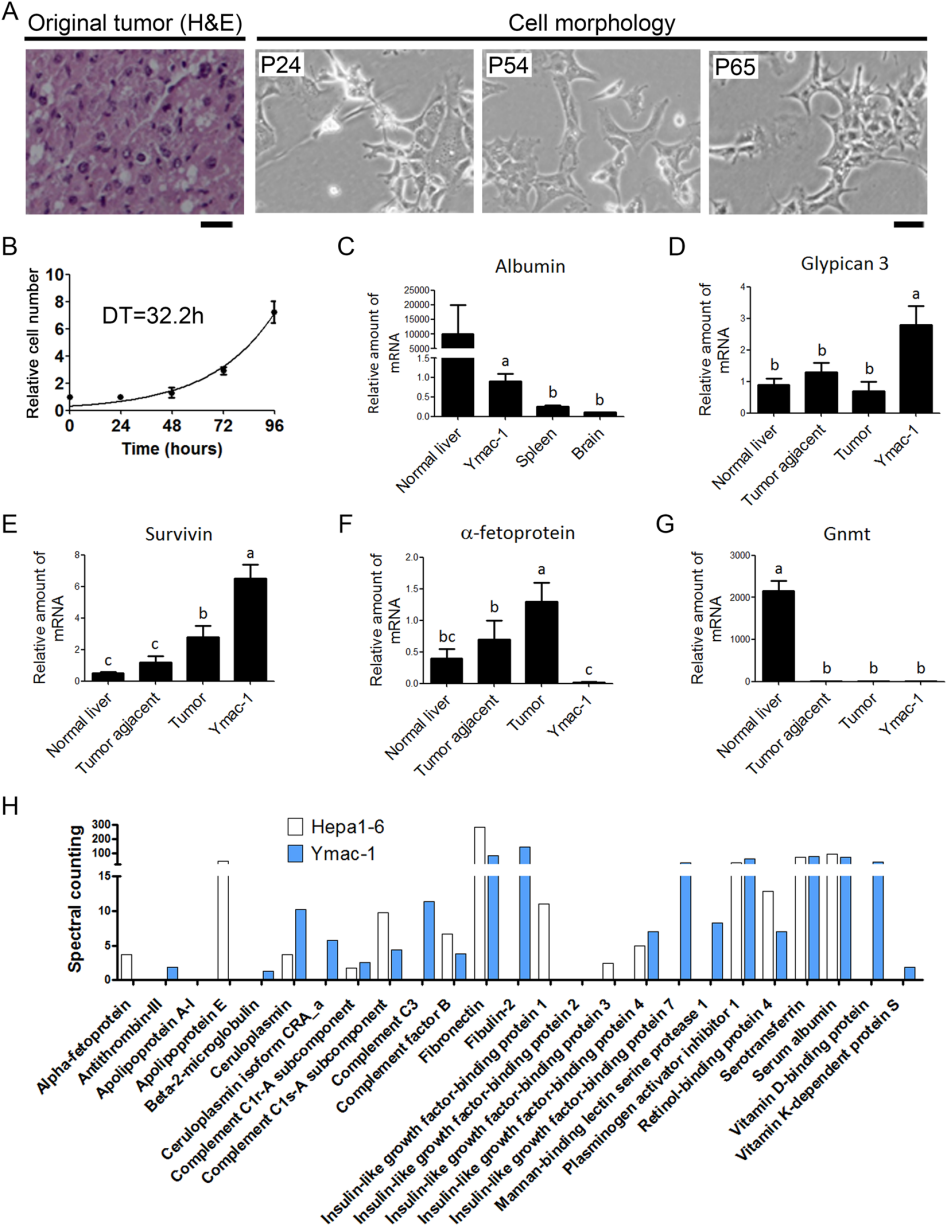
Establishment of Ymac-1 cell line. (**A**) H&E staining of original tumor and the cellular morphology of Ymac-1 at different passages. Bar: 25 μm. (**B**) Growth curve of Ymac-1 cell. Graphs show mean and s.d. of triplicate wells. DT, Doubling time. (**C**–**G**) mRNA expression levels of hepatocyte and HCC markers. n = 4~5 for samples from mice (normal liver, spleen and brain from wild-type mice, HCC tumor tissues and tumor-adjacent tissue from *Gnmt*−/− mice). RNA samples of Ymac-1 were harvested from 3 replicates. Data is presented as mean ± s.d. Means with the same letter are not statistically significant using one-way ANOVA test (*P* < 0.05). (**H**) Proteomic analyses of culture supernatant of Ymac-1 and Hepa1-6 cells cultured in serum free DMEM. The relative amount were quantified by spectral counting.

We removed the liver tumor from a 22-month old *Gnmt*−/− mice. A small part of the tumor was fixed in formalin for histology examination. In consistency with our previous finding, the H&E staining revealed a typical HCC morphology with severe steatosis of liver tumor (Fig. 1A). The remaining tumor were minced and digested with collagenase and trypsin. The resultant cells were maintained on petri-dish in DMEM, and denominated as Ymac-1. We successively subcultured Ymac-1 cells for more than 100 passages. Pictures of the morphology of Ymac-1 cells showed elongated or triangular mesenchymal like cell shape (Fig. 1A). The morphology of Ymac-1 cells remained unchanged after the twentieth passage. The doubling time of Ymac-1 is around 32.2 hour (Fig. 1B). Next, we examined whether Ymac-1 cells express known hepatocyte and HCC markers. Compared to non-liver tissue (spleen and brain), Ymac-1 cells expressed a notable level of hepatocyte marker-Albumin despite the level still being far from that in normal liver tissue (Fig. 1C and Supplementary Fig. 1A). For HCC markers, Ymac-1 cells had a higher level of Glypican-3 and Survivin than liver tumor form *Gnmt*−/− mice, while they lack AFP expression (Fig. 1D–F and Supplementary Fig. 1B). The lack of Gnmt expression in all samples was also confirmed by qPCR (Fig. 1G). Next, we grew Ymac-1 cells in serum free medium and collected the culture supernatant for SDS-PAGE analysis. A remarkable signal around molecular weight of 69 KDa, which is believed to be albumin, was observed. A similar profile was observed in the cultured medium of a mouse HCC cell line-Hepa1-6 cell and in the serum of wild type mouse (Supplementary Fig. 1C). The SDS-PAGE gel was then used for proteomic analysis. In addition to Albumin, several liver secreted plasma proteins were also found in Ymac-1 cell cultured supernatant (Fig. 1H), which suggested the capability of Ymac-1 cells to produce and secret plasma proteins. Taken together, these findings suggested a hepatic origin of Ymac-1 cells.

### Ymac-1 cell shows sarcomatoid HCC characteristics

**Figure 2:**
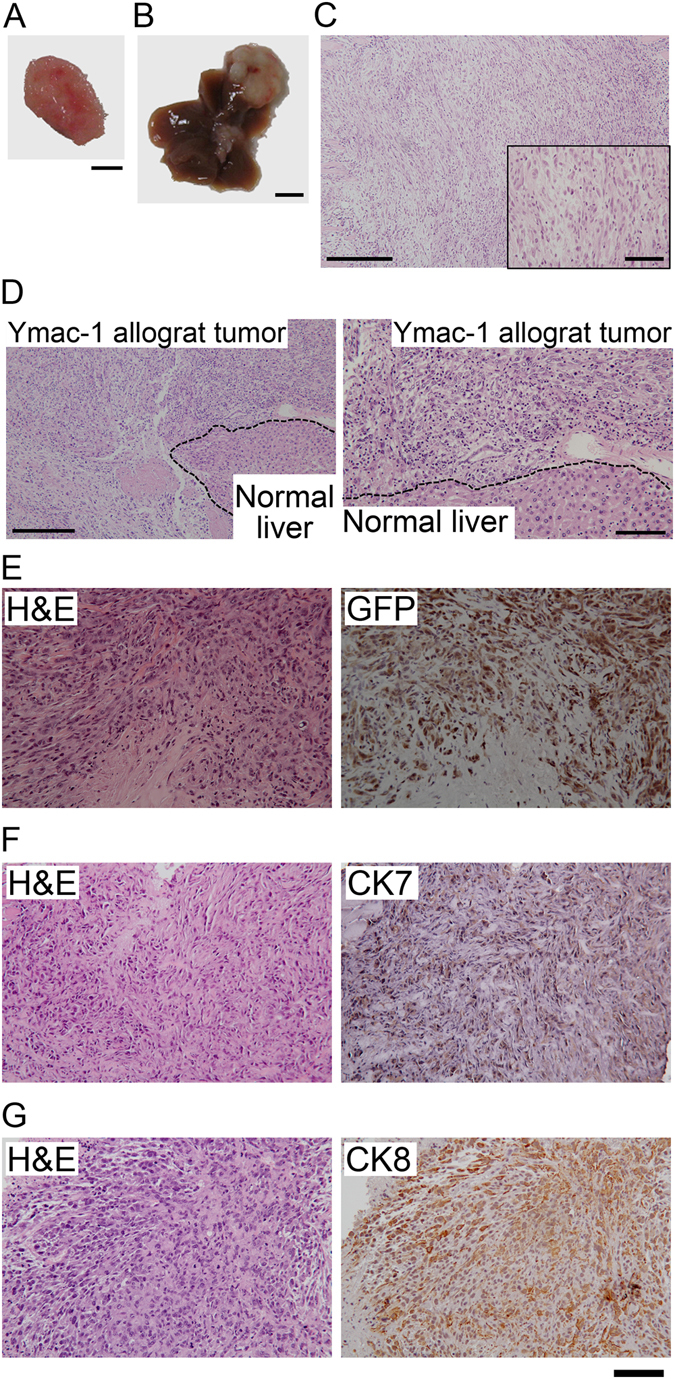
Ymac-1 cell showed sarcomatoid HCC characteristics. Gross pictures of Ymac-1 allograft tumor formed from subcutaneous (**A**) and intrahepatic (**B**) injection. Bar: 50 mm. (**C**) H&E staining of tumors from (**A**). Inset: higher magnification view. (**D**) H&E staining of tumors from (**B**). Right panel: higher magnification view of left panel. (**E**) H&E and IHC of GFP in GFP-expressed Ymac-1 allograft tumor section. (F-G) IHC of CK7 (**F**) and CK8 (**G**) and respective H&E staining in representative Ymac-1 allograft tumor section. Bars: 250 μm for lower magnification of (C and D), 50 μm for inset in (**C**), 100 μm for higher magnification of (**D**) and for (**E**) to (**G**).

To test whether Ymac-1 cell is tumorigenic, we transplanted 5 × 10^5^ Ymac-1 cells into NOD-SCID mice both subcutaneously (n = 3) and intrahepatically (n = 3). Allograft tumors were observed in all mice after 4 weeks of inoculation (Fig. 2A,B and Supplementary Fig. 2). To our surprise, pathological examination revealed a sarcomatoid appearance of all allograft tumors from Ymac-1 (Fig. 2C,D), while allograft tumor formed from Hepa1-6 cells displayed typical HCC morphological features (Supplementary Fig. 3). We stably expressed GFP in Ymac-1 cell, and used this cell to establish allograft tumor. Result from IHC staining with anti-GFP antibody demonstrated that the allograft tumor was developed from Ymac-1 cell (Fig. 2E). Since cytokeratin 7 and 8 (CK7 and CK8) are frequently used for differentiating SHC from true sarcomas^8, 15, 24^, we next determined the expression of these proteins in Ymac-1 allograft tumor. As shown in Fig. 2F and G, the allograft tumor formed from Ymac-1 cell was positive for both CK8 and CK7 staining. These findings together with the observation of lacking AFP expression in Ymac-1 cells (Fig. 1F) suggested that Ymac-1 cell is a sarcomatoid HCC cell line.

### Ymac-1 cell displays characteristics consistent with EMT

**Figure 3:**
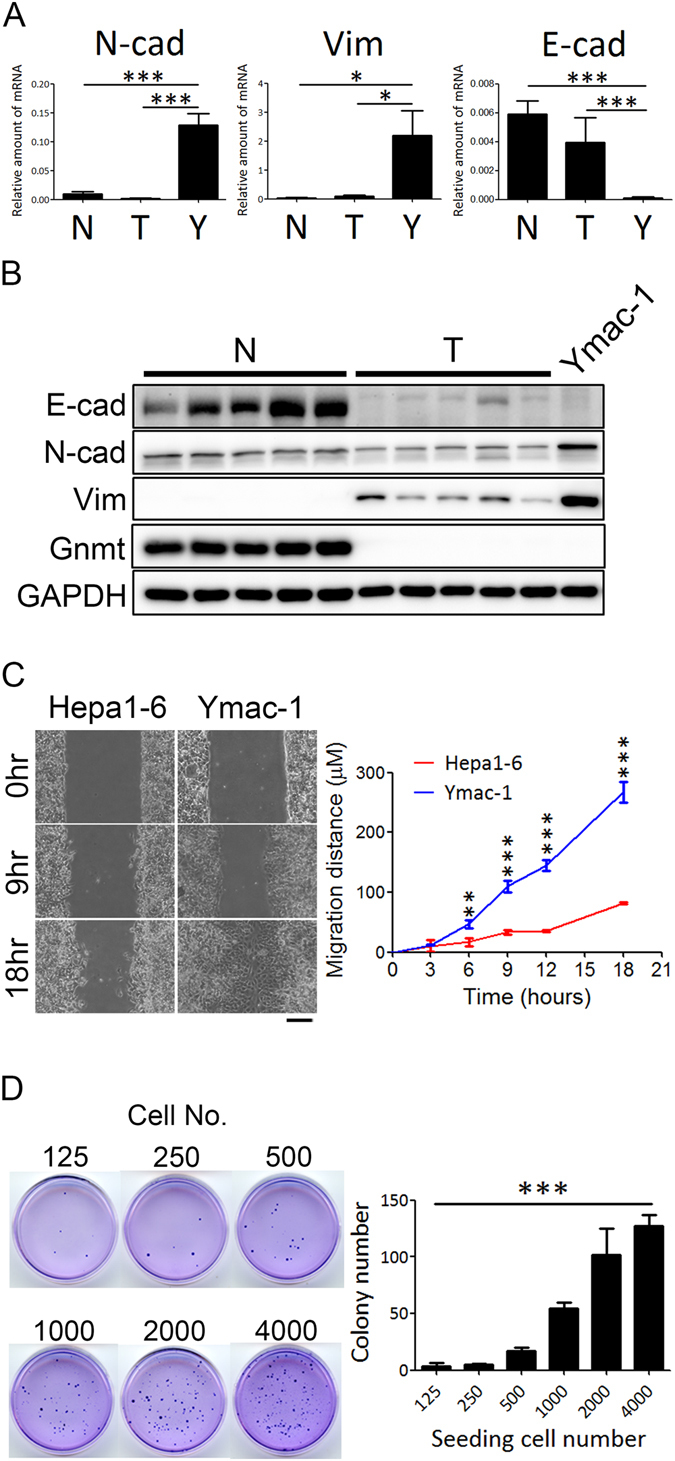
Ymac-1 cell displays characteristics consistent with EMT. (**A**) Determination of N-cadherein (N-cad), E-cadherin (E-cad), Vimentin (Vim) mRNA expression in normal liver (N, n = 3), HCC tissues from *Gnmt*−/− mice (T, n = 4) and Ymac-1 cells (Y). Data is presented as mean ± s.d. *P < 0.05 and ***P < 0.001, using Student’s t-test. (**B**) Western blot of analyses of N-cad, Vim, and E-cad in normal liver (N, n = 4), HCC tissues from *Gnmt*−/− mice (T, n = 4) and Ymac-1 cells. (**C**) Representative picture and quantitative results of wound healing assay of Ymac-1 and Hepa1-6 cells. Bar: 100 μm. Data is presented as mean ± s.d. **P < 0.01, ***P < 0.001, using repeated measures ANOVA analysis with a Bonferroni post hoc test. (**D**) Anchorage-independent growth of Ymac-1 cell. Right panel is the quantitative results. Data is presented as mean ± s.d. ***P < 0.001, significant linear trend over dose levels.

EMT has been implicated in the process of epithelial transdifferentiation into sarcoma/sarcoma-like cells^1, 2, 14^. As shown in Figs 1A and 2C, Ymac-1 cells rather displayed elongated, irregular fibroblastoid morphology than a typical polygonal epithelial morphology, and the allograft tumor from Ymac-1 cell showed a sarcomatoid morphology. We then examined whether Ymac-1 cell displays EMT-like characteristics. Compared to HCC samples from *Gnmt*−/− mice, the mRNA and protein level of N-cadherin and Vimentin increased dramatically in Ymac-1 cells, while E-cadherin protein expression was greatly reduced in Ymac-1 cells (Fig. 3A,B). Moreover, results from wound healing assay showed that Ymac-1 cells migrated more rapidly than Hepa1-6 cells (Fig. 3C). Since sarcomatoid HCC is a malignant liver tumor, we used anchorage-independent colony formation as a measure of malignant potential of Ymac-1 cell. As shown in Fig. 3D, Ymac-1 cells formed colonies in soft agar in a dose-dependent manner.

### Cancer stem cell like properties of Ymac-1 cells

**Figure 4:**
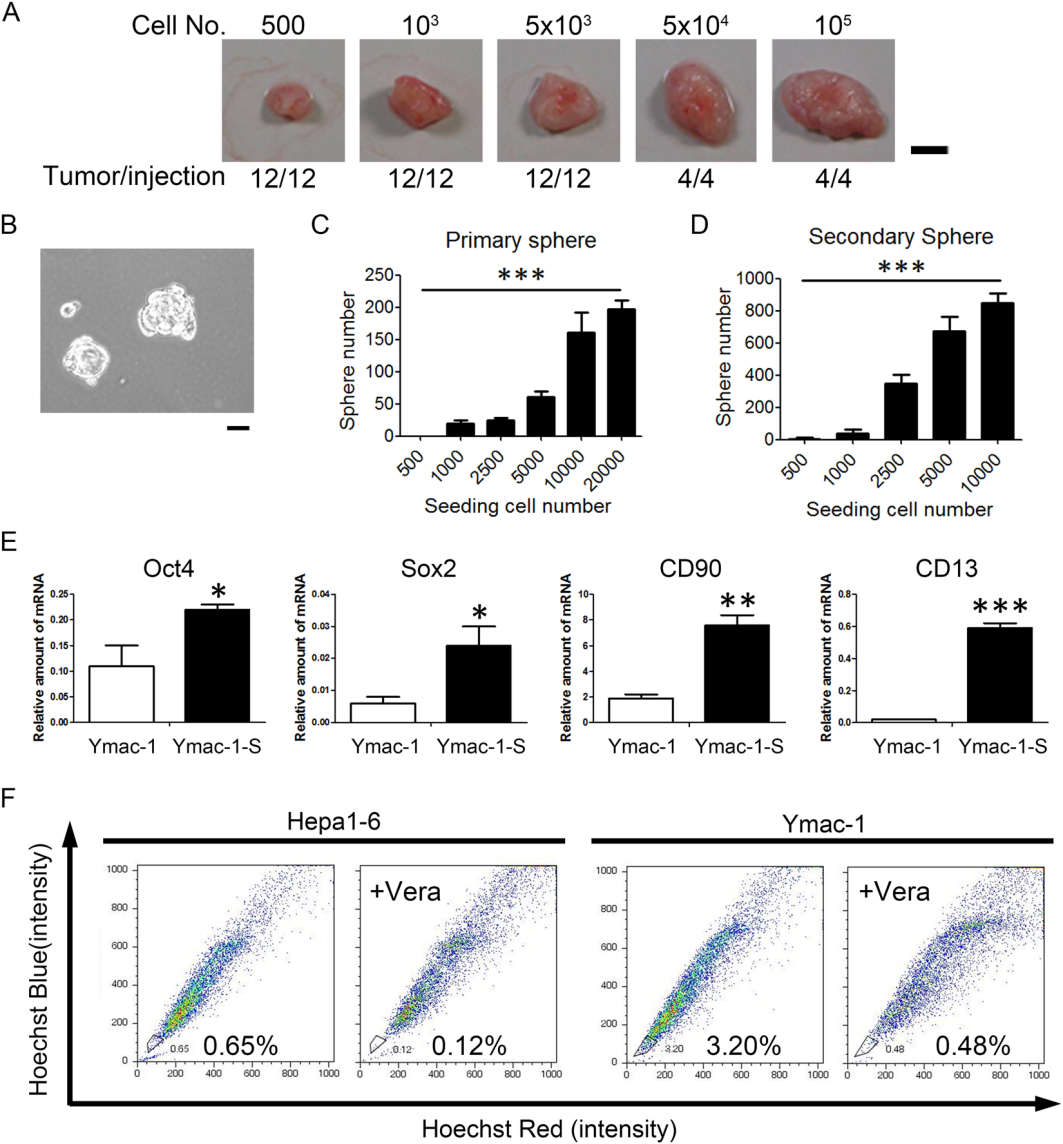
Cancer stem cell like properties of Ymac-1 cells. (**A**) Tumorigenicity of Ymac-1 cell. Different numbers of Ymac-1 cells were injected subcutaneously in NOD-SCID mice. All the injections developed tumor within 2 months. Bar: 50 mm. (**B**) Primary sphere formed from Ymac-1 cells. (**C**) Quantitative results of sphere-forming efficiency of (**B**). (**D**) Secondary sphere-forming efficiency of Ymac-1 cells. Data is presented as mean ± s.d. ***P < 0.001, significant linear trend overdose levels (for **C** and **D**). (**E**) The expression of stemness markers in non-sphere (Ymac-1) and sphere (Ymac-1-S) cells. Data is presented as mean ± s.d. *P < 0.05 and **P < 0.01, using Student’s t-test. (**F**) Side population (SP) assay. The SP cells are outlined and shown as a percentage of the total cell population. This cell population was reduced in the presence of verapamil (Vera).

Studies indicated that the cancer stem cells (CSCs) also expressed EMT markers. Moreover, CSC population can be increased by inducing EMT in transformed epithelial cells^25, 26^. Thus, we tested whether Ymac-1 cells also acquired CSC-like properties. We investigated the tumorigenic ability of Ymac-1 cells. Different numbers (from 10^5^ down to 500) of Ymac-1 cells mixed with matrigel were injected in the right flank of NOD-SCID mice. As shown in Fig. 4A, the tumorigenicity of Ymac-1 was 100% (12/12) even when only 500 cells had been injected. These data suggested that the Ymac-1 cell population is enriched in cells that are capable of initiating tumor in NOD-SCID mice. The sphere-forming efficiency is commonly used to evaluate the number of CSCs in a cancer cell line. We examined the sphere-forming efficiency of Ymac-1 cells by using this approach. The sphere-forming efficiency of Ymac-1 cells was around 2% (~200 spheres in 10,000 cells) (Fig. 4C). The sphere-forming efficiency of secondary sphere was even higher (~8%) indicating further enrichment of stem cells in the primary sphere cell population (Fig. 4D). Moreover, an elevated level of the stem cell markers such as Oct4, Nanog, Sox2, CD90, Grp78 and CD13 was observed in the spheres formed from Ymac-1 cells (Fig. 4E and Supplementary Fig. 4). The side population (SP) assay is a frequently used assay for isolating or enumerating stem-like cells in cancer^27, 28^. We examined Ymac-1 cells for a SP using DNA staining with the fluorescent dye Hoechst33342. SP cells were detected in Ymac-1 cells at frequencies of ~2.7%, while there was only ~0.5% SP cells observed in Hepa1-6 cell line (Fig. 4F). Together, our results suggested that a substantial population of Ymac-1 cells possess CSC-like characteristics.

### Microarray analysis of Ymac-1 cells

**Figure 5:**
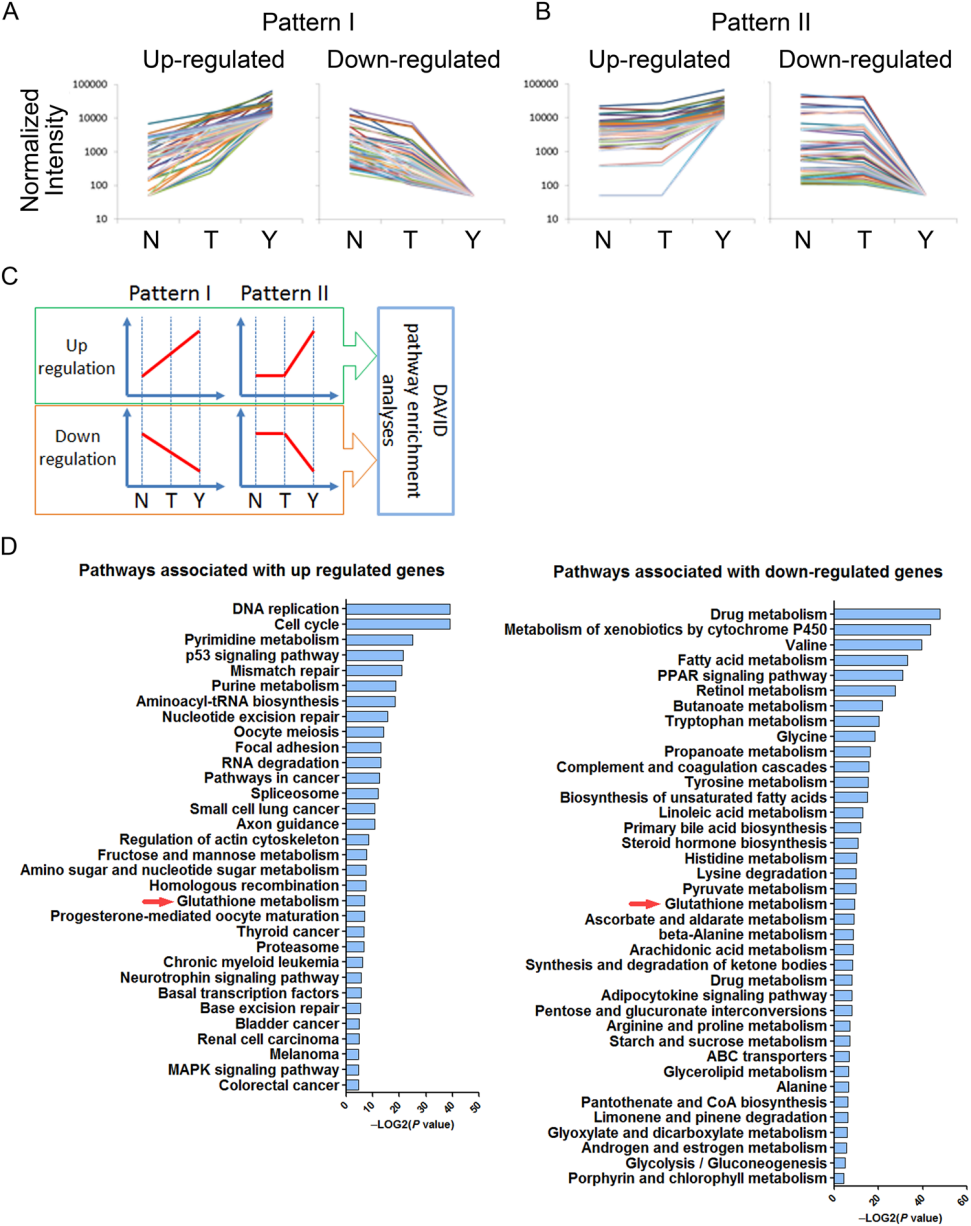
Microarray analysis of Ymac-1 cells. (**A**) Genes that were upregulated (or downregulated) progressively from mouse normal liver (N) to *Gnmt*−/− HCC (T) to Ymac-1 (Y) were categorized to pattern I. (**B**) Genes that were upregulated (or downregulated) only in Ymac-1 were categorized to pattern II. (**C**) Schematic illustration of pathway enrichment analyses. (**D**) Pathways that significantly associated with altered genes. Only the glutathione metabolism pathway showed significant association with both up- and down-regulated genes.

Our findings indicated that Ymac-1 cell is a malignant cell line with sarcomatoid HCC characteristics. In order to identify potential markers and therapeutic targets of sarcomatoid HCC, microarray analysis was performed with RNA prepared from Ymac-1 cells. The results were used to compare with the microarray data of liver tissues from wild type mice and HCC tissues from *Gnmt*−/− mice (GEO accession number: GSE9809)^21^. We focused on the genes that progressively up- or down-regulated from normal liver to HCC, then to sarcomatoid HCC (pattern I); and the genes changed only in sarcomatoid HCC (pattern II) (Fig. 5A,B). There are 785 and 78 progressively up- and down-regulated genes, respectively, belonging to pattern I; and 1681 and 1025 genes up- and down-regulated, respectively, only in sarcomatoid HCC which were categorized as pattern II (Supplementary Table 1). All the up- and down-regulated genes of pattern I and II were used separately for pathway enrichment analyses by DAVID (http://david.abcc.ncifcrf.gov/) (Fig. 5C). Only the glutathione metabolism pathway showed significant enrichment associated with both up- and down-regulated gene lists (Fig. 5D). Among the altered genes involved in glutathione metabolism pathway (Supplementary Table 2), two genes, glucose-6-phosphate dehydrogenase (G6pd) and 6-phosphogluconate dehydrogenase (6Pgd), called our attention. G6PD is a rate-limited enzyme of the pentose phosphate pathway (PPP). The main physiological function of the PPP is to produce NADPH and ribose 5-phosphate. There are two functions of NADPH: first, it provides reducing power and acts as a vital cofactor for many enzymatic reactions in various macromolecular biosynthesis pathways; second, it is also a crucial antioxidant, reducing the reactive oxygen species (ROS) production during rapid cell proliferation. Since G6PD is the vital enzyme and is responsible for the production of NADPH in PPP^29^, we next investigated the role of G6pd in Ymac-1 cells.

### G6PD is associated with CSC-like characteristics

**Figure 6:**
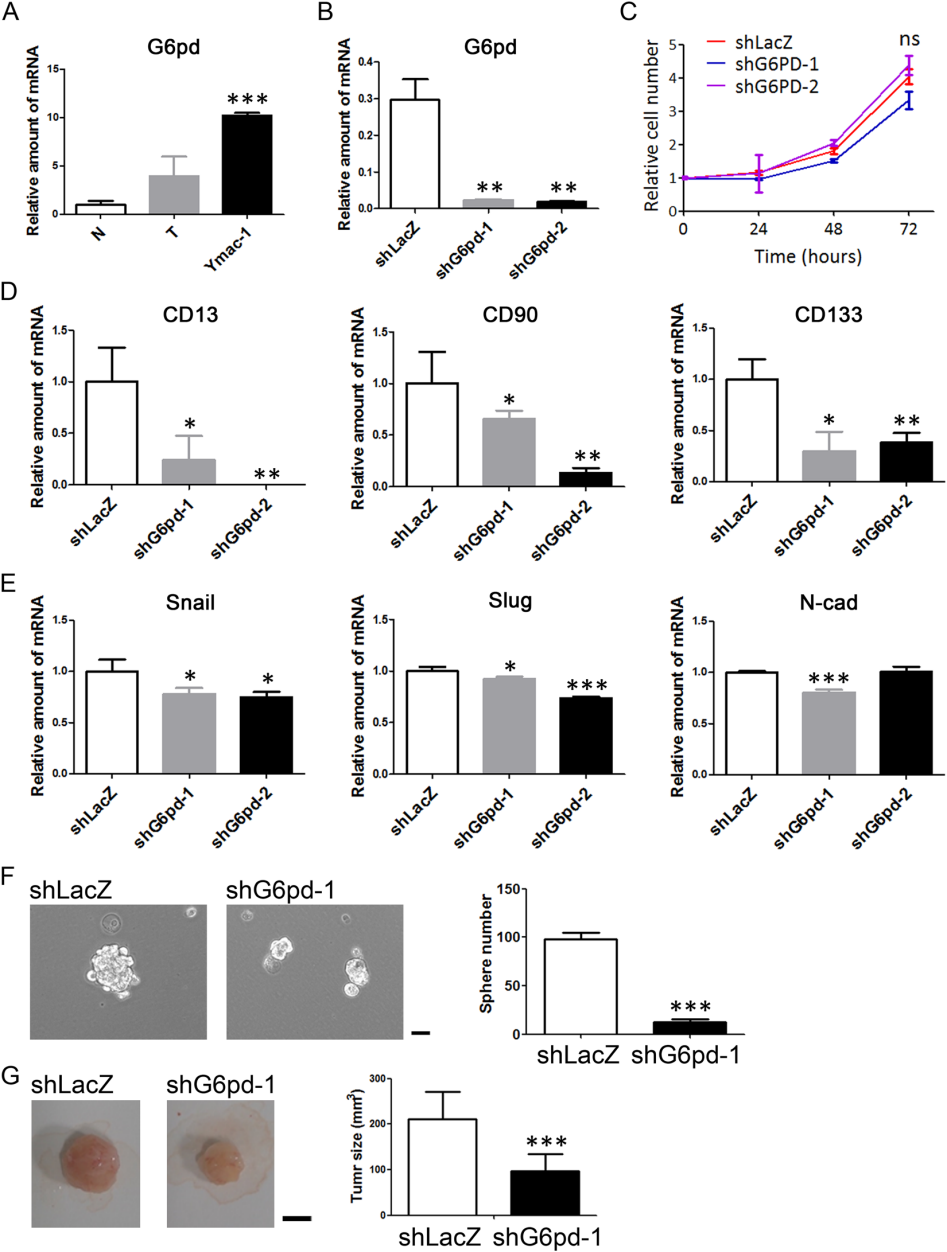
G6pd is associated with CSC-like characteristics of Ymac-1. (**A**) Determination of G6pd mRNA expression in normal liver (N, n = 4), HCC tissues from *Gnmt*−/− mice (T, n = 4) and Ymac-1 cells. (**B**) Determination of G6pd mRNA expression in G6pd knockdown (shG6pd-1 and shG6pd-2) and control (shLacZ) Ymac-1 cells. (**C**) Proliferation assay of cells in (**B**). ns, non-significant using one-ANOVA test. (**D**–**E**) mRNA expression levels of CSC (CD13, CD90 and CD133) and EMT (Snail, Slug and N-cad) markers in G6pd knockdown cells. (**F**) Sphere-forming efficiency of G6pd knockdown Ymac-1 cells. n = 4. Bar: 50 μm. (**G**) Tumorigenicity of G6pd knockdown Ymac-1 cells. n = 8 Bar: 50 mm. Data is presented as mean ± s.d. *P < 0.05, **P < 0.01 and ***P < 0.001, using Student’s t-test.

Microarray result showed that G6pd expression was up-regulated progressively from normal liver to HCC and to Ymac-1 (pattern I). These observation were confirmed by real-time PCR (Fig. 6A). To study the effects of G6pd on the characteristics of sarcomatoid HCC, we knocked its expression down in Ymac-1 cells (Fig. 6B). No obvious change in morphology was observed in G6pd-knock down Ymac-1 cells (Supplementary Fig. 5A). The proliferation rate was not affected by G6pd depletion (Fig. 6C). Regarding the markers of EMT and CSC, knocked G6pd down in Ymac-1 cell resulted in significant reductions in the expression of CSC markers such as CD13, CD90 and CD133, while only slight changes (although the differences were statistically significant) in Slug, Snail and N-cad (EMT markers) were observed (Fig. 6D,E). Moreover, the sphere forming and tumorigenic abilities of Ymac-1 cells were reduced significantly upon G6pd depletion (Fig. 6F,G). Pathological examination revealed a sarcomatoid appearance of allograft tumors formed from G6pd knock down Ymac-1 cells (Supplementary Fig. 5A), which was consistent with our previous finding that G6pd depletion caused vague changes on the expression of EMT markers. A notable reduction in CD133, but not CK7 and Vimentin, expression was observed in G6pd knocked-down Ymac-1 tumor, which further supported the notion that G6pd is associated with CSC-like characteristics (Supplementary Fig. 5B–D).

**Figure 7:**
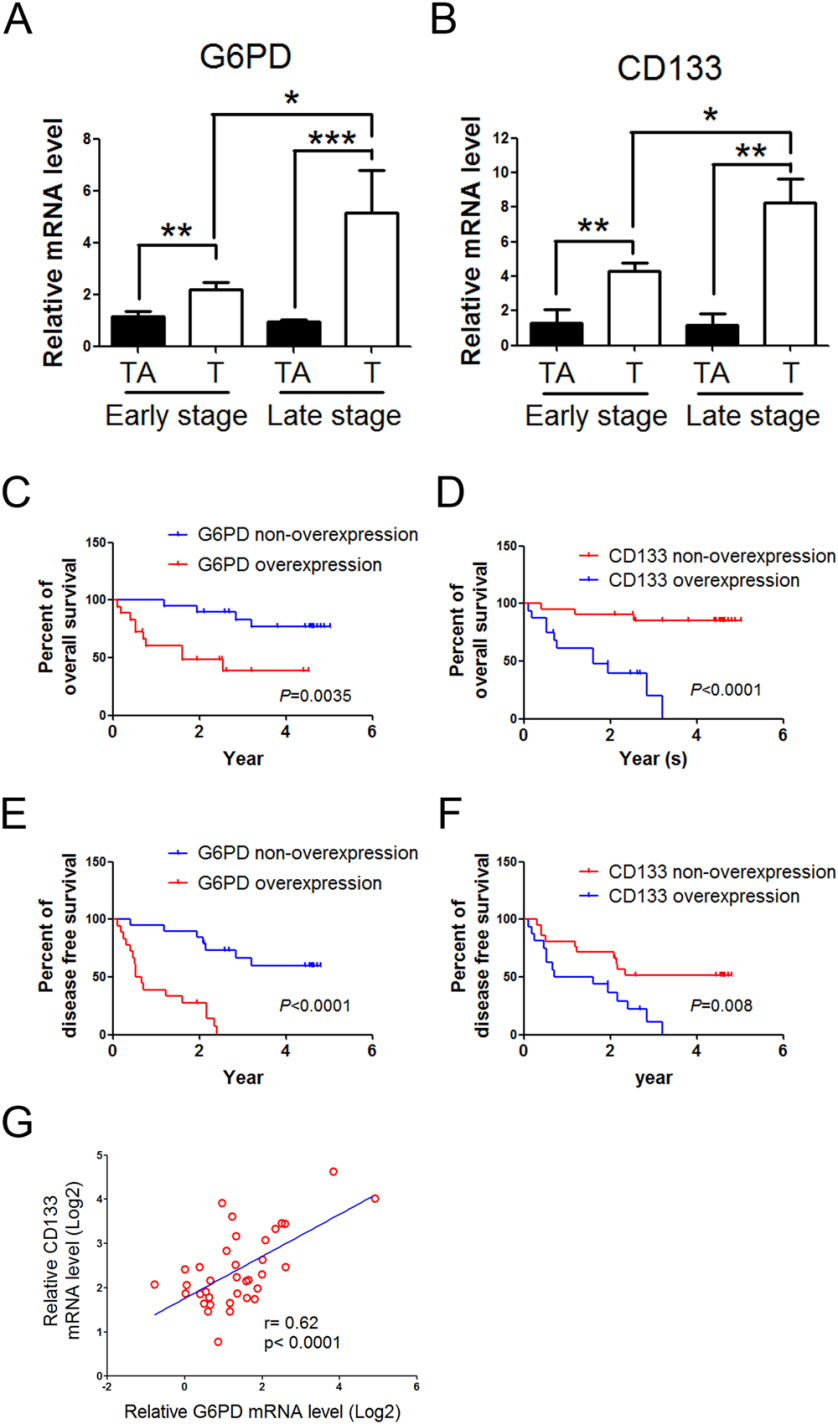
The positive correlation between G6PD and CD133 expression levels in HCC patients. (**A**,**B**) The expression of G6PD and CD133, respectively, in patients with HCC by real-time PCR. Data are presented as mean ± SEM. *P < 0.05, **P < 0.01 and ***P < 0.001, using Student’s t-test. (**C**–**F**) Kaplan-Meier analysis of overall survival (**C** and **D**) and disease-free survival (**E** and **F**) in 37 HCC patients with low or high G6PD (**C** and **E**) or CD133 (**D** and **F**) expression. The *P* values were calculated using the log rank test. (**G**) Pearson correlation analysis of G6PD and CD133 mRNA levels in tumor tissues.

To further determine the clinical relevance of G6PD and CSC-like characteristics in HCC, we conducted expression analyses of G6PD and CD133 based on real-time PCR data derived from human HCC cohorts. Up-regulation of G6PD and CD133 were observed and well correlated with pathological stage in HCC specimen (Fig. 7A,B and Supplementary Table 3). Lower expression of G6PD or CD133 were significantly associated with better disease-free survival and overall survival in these patients (Fig. 7C–F and Supplementary Table 4). Notably, a statistically significant positive correlation was found between G6PD and CD133 mRNA levels in tumor tissues (r = 0.62; *P* < 0.0001, Fig. 7G).

## Discussion

In this study, we established a liver cancer cell line from *Gnmt*−/− mice and designated it as Ymac-1. Although Ymac-1 cells expressed hepatocyte and HCC markers, this cell displayed elongated, irregular fibroblastoid morphology. Histological examination of the allograft tumor formed from Ymac-1 cells revealed a sarcomatiod appearance. Positive of CK7/CK8 staining indicated that Ymac-1 is a sarcomatoid HCC cell line. Further studies demonstrated that Ymac-1 cells displayed EMT phenotypes and contained a substantial population that possessed CSC-like properties, which resemble the clinical observations of sarcomatoid HCC.

It is interesting to note that almost all pathological examination of the liver tumor from *Gnmt*−/− mice showed typical HCC appearance with severe fatty change rather than sarcomatoid HCC phenotype. Then why do Ymac-1 cells possess characteristics of sarcomatoid HCC? Two factors might contribute to the sarcomatoid appearance. First, it has been proposed that stem cell/progenitor cells in *Gnmt*−/− mice liver take the place of hepatocyte for replenishing the liver parenchyma and ultimately form liver tumors^23^. Therefore, it is reasonable that liver tumor from *Gnmt*−/− mice should be highly heterogeneous with substantial stem cells/progenitor cells-like subpopulations that could further differentiate to different cell types. Second, the process of primary culture is bona fide a selection process. Only those cells possess strong growth capability and survivability that could be sustained for a long time. Thus, it is sensible to imagine that the population possess stem cells/progenitor cells properties which will be enriched during the primary culture process. Our culture condition seems to favor the commitment of mesenchymal lineage. The notion was strengthened by the following observation. We established two other cell lines from the liver tumor of *Gnmt*−/− mice. The Ymac-4 and Ymac-5 cells, established by the same condition as Ymac-1 cells, also showed an elongated, irregular fibroblastoid cell morphology. A cholangiocarcinoma-like appearance with spindle shape cell morphology was found in the allograft tumor formed from Ymac-4 cells. The morphology of allograft tumors from Ymac-5 cells was a mixture of sarcomatous (major) and adenocarcinomatous (few foci) elements (Supplementary Fig. 6). The other interesting question is whether *Gnmt* deficiency play crucial role for developing sarcomatoid morphology of Ymac series cell lines? We had reintroduced human GNMT back into Ymac-1 cells. However, compared to GFP overexpressed control Ymac-1 cells, neither the cell/tumor morphologies nor the expression profile of EMT/CSC markers were changed in GNMT overexpression Ymac-1 cell (data not shown). These results indicated that reintroducing GNMT back into Ymac-1 cell cannot change phenotype from sarcomatoid to ordinary HCC. Nonetheless, these findings also cannot exclude the possibility that GNMT deficient liver progenitor/stem cells are more susceptible for transdifferentiation into sarcoma-like cells; and further investigation is needed to evaluate the role of GNMT in this transdifferentiation.

To the best of our knowledge, only two sarcomatoid HCC cell lines have been reported. Kim *et al*. established a cell line, designated as SH-J1, from a patient that was diagnosed as sarcomatoid HCC with negative for serologic markers of hepatitis B and C virus^30^. Tai and his colleagues reported the other sarcomatoid HCC cell line and designated as SAR-HCV which was established from a liver lesion of a patient with HCV-related liver tumor^31^. There are plenty reports utilizing SH-J1 cell for studies related to HCC tumorigenesis, EMT and drug development^32–34^. SH-J1 and Ymac-1 both have spindle morphology, express EMT markers such as N-cadherin and Vimentin, and are tumorigenic on immunodeficient mice. In addition, several genes expressed similarly in SH-J1 and Ymac-1 were also observed (Supplementary Table 5). However, the CSC-like characteristics have never been determined in SH-J1 cell. In present study, we demonstrated that Ymac-1 cell not only displays EMT characteristics, but also possesses CSC-like properties. The side population (SP) of commonly used human HCC cell lines are ranged from 0% to 2%^35^. Ymac-1 cells contain SP cells in ~2.5%. Thus Ymac-1 can be considered as a relatively high SP cell model.

It is noteworthy that Ymac-1 originated from mouse that lacked Gnmt, an important enzyme with tumor suppressive function and is highly down-regulated in HCC^20^. The liver cancer of *Gnmt*−/− mice has an ordinary HCC morphology^21^. Since they have the same genetic background, the comparison of gene expression profile between Ymac-1 and *Gnmt*−/− liver tumor would provide insight into the underlying mechanism for sarcomatoid HCC formation. Ymac-1 and *Gnmt*−/− liver cancer can also serve as good models for identifying specific markers and/or therapeutic targets for sarcomatoid HCC. In addition, Ymac-1 is capable of forming tumors (both orthotopic and subcutaneously) in B6 mouse. Therefore, Ymac-1 will be an excellent cell for studying the interaction between sarcomatoid HCC and host immune-system or tumor micro-environment in *in vivo* models which are more close to clinical conditions.

Although sarcomatoid HCC has been considered as a rare histologic variant of HCC^36^, it is believed that it was underestimated due to the highly heterogeneous nature of HCC and the lack of diagnostic modalities for sarcomatoid HCC. More importantly, the potential therapeutic targets for sarcomatoid HCC have not been investigated thoroughly. Here, we used Ymac-1 cell as a sarcomatoid HCC model for microarray analysis and identifying genes highly and specifically expressed in Ymac-1 cells. This information could be useful for developing biomarkers and therapeutic modalities for sarcomatoid HCC. As a demonstration, we investigated the correlation between G6PD and characteristics of sarcomatoid HCC. G6PD, the first and rate-limiting enzyme of PPP pathway, has been reported to overexpress in HCC specimen and its expression correlates well with pathological stage and poor survival^37^. We observed same correlations in this study. Interestingly, depletion of G6PD in Ymac-1 cells led to substantial reduction in the expression of CSC markers, the sphere formation ability and the tumorigenesis. A significant correlation between the expression of G6PD and CD133 was also observed in human HCC specimen. These findings suggested that G6PD could hold the potential for targeting sarcomatoid HCC as well as CSC-like population in HCC, which is crucial for tumor initiation, drug resistance and relapse. An early study has demonstrated that inhibition of G6PD reduced carcinogenesis in experimental rat model by decreasing the growth and progression of persistent liver nodules^38^. Thus, it is reasonable and worthy to explore the feasibility and applicability of combination of G6PD inhibition and conventional HCC therapies.

## Materials and Methods

### Primary culture

All mice were kept in a 12-hour light-dark–cycle room with water and standard mouse pellet chow. All animal experiments were performed in accordance with the Laboratory Animal Welfare Act, Guide for the Care and Use of Laboratory Animals evaluated and approved by the Institutional Animal Care and Use Committee of Kaohsiung Medical University. Liver tumor from a 22 month old *Gnmt*−/− mouse was separated and divided into several parts for pathological examination, DNA/RNA/protein analyses, and primary culture. Detail procedure was described in Supplementary Materials and Methods.

### Allograft tumor morphology and tumorigenesis assay

All allograft tumor formation assays were carried out as described previously^39^. The NOD-SCID mice (6–8 week old, male) were obtained from National Laboratory Animal Center (NLAC), National Applied Research Laboratories (NARL), Taiwan. The mice were maintained in Specific Pathogen-Free (SPF) environment at Laboratory Animal Center of Kaohsiung Medical University. Mice were intrahepatically or subcutaneously (in the right flank of legs) inoculated with 1 × 10^6^ cells using 50% matrigel (Life Technologies). Eight weeks after inoculation, all the mice were sacrificed. The tumor size was measured every 2–3 days by using caliper and tumor volume was calculated as: L × W^2^ × 0.5, where L is the longest diameter and W is the shortest diameter. For tumorigenesis assay, different amount of Ymac-1 cell (from 100 to 10,000) were injected into NOD-SCID mice subcutaneously. Mice were sacrificed when 1) tumor size reach to 200 mm^3^ or 2) four months after inoculation. Allograft tumors were collected and cut into pieces for H&E and IHC staining, and for DNA, RNA, and protein analyses.

### RNA isolation, reverse transcription and real-time PCR

Detail procedure was described in Supplementary Materials and Methods. The primer pairs used are shown in Table 1.

**Table 1:** List of real-time PCR primers used in the study.

GENE	FORWARD PRIMER(5′->3′)	REVERSE PRIMER(5′->3′)
CD90	ATCCCCCAGACAGCGAGAGT	GCCTGCCCCTGAGATTAGG
Oct4	CTGTAGGGAGGGCTTCGGGCACTT	CTGAGGGCCAGGCAGGAGCACGAG
Nanog	AGGGTCTGCTACTGAGATGCTCTG	CAACCACTGGTTTTTCTGCCACCG
Sox2	TAAGGGTTCTTGCTGGGTTTT	AGACCACGAAAACGGTCTTG
Grp78	TCTTGCCATTCAAGGTGGTTG	TTCTTTCCCAAATACGCCTCAG
CD44	AGCTGACGAGACCCGGAAT	GCGTAGGCACTACACCCCAAT
CD133	GGCTGGGTGGCTTGATTGT	CAGCAAGCCCAGGAAAAAGA
CD13	CCGCCCCTCCGAGTTTA	AGTGATCCTCCTGTCCACTTTTG
Glypican 3	CCAACGCCATGTTCAAGAATAAC	TGAAAAATTCACCGACAAACTCAA
Survivin	CCTACCGAGAACGAGCCTGAT	GGGTTCCCAGCCTTCCAA
Afp	TCTGCTGGCACGCAAGAAG	TTGCAACTCTCGGCAGGTT
Gnmt	GTTGACGCTGGACAAAGA	AGCCTGTGCTGAGGATA
Gapdh	TCACCACCATGGAGAAGGC	GCTAAGCAGTTGGTGGTGCA
Albumin	GAAAACCAGGCGACTATCTCCA	TGCACACTTCCTGGTCCTCA
E-cadherin	ACTGTGAAGGGACGGTCAAC	GGAGCAGCAGGATCAGAATC
N-cadherin	GGCAGAAGAGAGACTGGGTC	GAGGCTGGTCAGCTCCTGGC
Vimentin	ACTCACCTGTGAAGTGGATGC	TGGTATTCACGAAGGTGACG
Snail	TGTCCAGAGGCTACACCTCA	CTCACTGCCAGGACTCCTTC
Slug	GATGTGCCCTCAGGTTTGAT	ACACATTGCCTTGTGTCTGC
G6pd	GCATCATCGTGGAGAAGC	TGTTGGCAAACCTCAGC
G6PD (human)	CAACATCGCCTGCGTTA	CTTGACCTTCTCATCACGG
TBP (human)	CAGAAGTTGGGTTTTCCAGCTAA	ACATCACAGCTCCCCACCAT

### Western blotting

Cell and tissue lysates preparation and Western blotting were performed as described previously^39^. Detailed procedure can be found in Supplementary Materials and Methods. The antibodies used were: Mouse-Anti-GNMT monoclonal antibody^40^; Goat-Anti-vimentin polyclonal antibody (Santa Cruz); Rabbit-Anti-N-cadherin polyclonal antibody (Abcam); Rat-Anti-E-cadherin monoclonal antibody (Abcam); Mouse-Anti-GAPDH (Millipore).

### Immunohistochemical (IHC) Staining

Detailed procedures for IHC staining have been described previously^39^. Signals were visualized using SuperPicTure™ Polymer Detection kits (Life Technologies). The antibodies used were: Rabbit-Anti-Cytokeratin 7 polyclonal antibody (Novus); Rat-Anti-Cytokeratin 8 monoclonal antibody (DSHB); Goat-Anti-Vimentin polyclonal antibody (Santa Cruz).

### Cell proliferation assay

Cells were seeded in a 96 well plate (1,000 cells per well) in at least triplicate for each experiment. At indicated time point, alamar Blue® (10%, Thermo Fisher Scientific) reagent was added and further incubated for 4 h at 37 °C. Fluorescence of the reduced alamar Blue® was measured by microplate reader (Synergy HT, BioTek Instruments). The amount of fluorescence for each group was normalized to the next day of seeding (day1) and shown as fold increase. All data are presented as mean ± SD.

### Proteomic experiment

Ymac-1 and Hepa1-6 cells were seeded for 24 hours, washed with PBS, then cultured in serum free DMEM for additional 24 hours. The cultured medium was collected and centrifuged at 2,000 rpm, 4 °C for 5 min (Allegra™64R, Beckman). The supernatant was concentrated using Vivaspin concentrators (3,000 Da molecular-weight cutoff) (Sartorius) at 3,000 × g for 15 min (4 °C). Concentrated samples were separated by SDS-PAGE. Coomassie Brilliant Blue R-250 was used to stain the gel. The digested sample preparation, mass spectrometry (MS) analyses and protein identification and quantification were performed as described previously^41^.

### Wound healing assay

Cell culture migration insert (ibidi) was placed into six-well plate. Cells were trypsinized and resuspended in complete tissue culture medium and were placed into each side of the insert. The inserts were removed 24 hours later and pictures were taken once every three hours.

### Sphere formation assay

Cells were washed with PBS to remove serum and suspended in serum-free DMEM/F-12 medium with N2 supplement (Life Technologies), 20 ng/ml EGF and 20 ng/ml basic-FGF (Peprotech). Cells were subsequently cultured in ultra-low attachment plates (Corning) for 2 weeks to allow tumor sphere formation. The spheres were counted under microscope. For sphere passage, spheres were collected and dissociated by trypsin, then cultured as mentioned above.

### Soft agar assay

For the soft agar assay, a bed of 0.6% agar in DMEM/10% FCS (bottom agar, SeaKem) was prepared in 3.5 cm culture dishes. Different numbers of cells were mixed with 0.3% agar in DMEM/10% FCS (top agar) and poured onto the 0.6% agar bed. Fresh medium was added periodically. The number of foci was counted after 3 weeks.

### Side-population (SP) assay

The protocol was based on Goodell *et al*.^42^ with slight modifications. Briefly, cells were incubated in prewarmed DMEM/2% FBS containing freshly added Hoechst 33342 (5 μg/mL final concentration) (Sigma-Aldrich) for 90 minutes at 37 °C with intermittent mixing. In some experiments, cells were stained with the Hoechst dye in the presence of Verapamil (100 μM, Sigma-Aldrich). After incubation, cells were collected by centrifugation and resuspended in ice-cold PBS. Cells were stained with propidium iodide (10 μg/mL final concentration) for dead cell discrimination. Cells were subjected to analysis using FacsAria (BD Biosciences).

### Microarray analysis

Total RNA was extracted as described previously. The RNA quality was accessed using Agilent 2100 Bioanalyzer (Agilent Technologies). One μg total RNA samples and Cy5 dyes (Amersham Pharmacia) were used to prepare fluorescent antisense RNA (aRNA) by using OneArray® Amino Allyl aRNA Amplification Kit (Phalanx Biotech Group). Fluorescent aRNA were hybridized to the Mouse Whole Genome OneArray® with Phalanx hybridization buffer using Phalanx Hybridization System. Detail procedure is described in Supplementary Materials and Methods. The microarray data are available at GSE88827. The spots with log2 ratio ≥ 1 or log2 ratio ≤−1 and *P* value < 0.05 are tested for further analysis. Differentially expressed genes were annotated and pathway enrichment analysis was performed by using DAVID Bioinformatics online tools (Database for Annotation, Visualization and Integrated Discovery; http://david.abcc.ncifcrf.gov/)^43^.

### Knock down experiment

Lentiviral plasmids encoding different shRNAs for *G6PD*: shG6PD-1 (5′-GAGGAGTTCTTTGCCCGTAAT-3′) and shG6PD-2 (5′-ACGTGGTCCTTGGCCAATATG-3′), the control plasmids for the RNA interference (pLKO.1-shLacZ), the packaging plasmid (pCMV-ΔR8.91), and the envelope plasmid (pMD.G) were obtained from National RNAi Core Facility, Academia Sinica, Taiwan. Lentivirus production, infection and stable cell selection were carried out according to the protocol published on the web site of RNAi Core (http://rnai.genmed.sinica.edu.tw/). Detailed procedure is described in Supplementary Materials and Methods.

### Hepatocellular Carcinoma (HCC) patients

The RNA samples of HCC patients were obtained from Taiwan Liver Cancer Network (TLCN, http://tlcn.nhri.org.tw/TLCN/index.jsp). Informed consent was obtained from all the patients before they received surgery. In addition, clinical and pathological data including duration of survival were provided by TLCN. This study was approved by the Institutional Review Board of Kaohsiung Medical University Hospital and the user committee of TLCN. Detailed characteristics of all patients are summarized in Supplementary Tables 3 and 4.

### Statistical Analysis

All data were analyzed using GraphPad Prism 5.01 software (La Jolla, CA, USA) and *P* value < 0.05 was considered to be statistically significant. Statistical analyses were made by using Student’s t test and one-way ANOVA with Tukey’s post-test or a post-test for linear trends. Repeated measurement ANOVA was applied for the comparison of migration distance in wound healing assay. The Kaplan-Meier estimation method was used for survival analysis, and a log-rank test was used to compare differences. *P* values for significance are indicated as follows: *P < 0.05; **P < 0.01; ***P < 0.001.

## Electronic supplementary material

Supplementary materials
