# Supplementary material for: Characterization of a new murine cell line of sarcomatoid hepatocellular carcinoma and its application for biomarker/therapy development
Source: Sci Rep. 2017 Jun 8;7:3052. doi: 10.1038/s41598-017-03164-3 (PMC5465177; doi:10.1038/s41598-017-03164-3)
Supplement: Supplementary file 1 — Supplementary materials [file 41598_2017_3164_MOESM1_ESM.pdf]

Supplementary material

Characterization of a new murine cell line of sarcomatoid hepatocellular carcinoma and its application for biomarker/therapy development

Chia-Hung Yen, Chih-Chung Lai, Chen-Chung Liao, Sheng-Fan Wang, Yi-Jen Liao, Chien-Yi Tung, Jung-Hsien Hung, Shiu-Feng Huang, Yi-Ming Arthur Chen*

Table of content

Supplementary Materials and Methods

Supplementary Fig 1 – Fig 6

Supplementary Table 1 – 5

Supplementary References

Supplementary Materials and Methods

Primary culture

All mice were kept in a 12-hour light-dark–cycle room with water and standard mouse pellet chow. All animal experiments were performed in accordance with the Laboratory Animal Welfare Act, Guide for the Care and Use of Laboratory Animals evaluated and approved by the Institutional Animal Care and Use Committee of Kaohsiung Medical University. Liver tumor from a 22 month old *Gnmt-/-* mouse was separated and divided into several parts. One part was fixed in buffered formalin to perform hematoxylin and eosin (H&E) stain and immunohistochemical (IHC) staining (see below). Three parts were stored in liquid nitrogen for DNA, RNA, and protein analyses. The remnant tumor tissue was cut into pieces about 1 cm^3 in size. Each piece was minced in 0.5ml trypsin with scissors under sterile conditions. Minced tissue was filtered through a sterile iron mesh to remove large tissue clump. The resultant cell suspension was subjected to collagenase/trypsin treatment for 16–18h at 37ºC in a petri dish. The digested supernatant was collected and washed with DMEM supplemented with penicillin (100 U/ml) and streptomycin (100 μg/ml) (Thermo Fisher Scientific); and then subjected to additional collagenase treatment for 90 min at 37◦C. The digested supernatant was collected and washed as described above; then it was resuspended and subcultured in DMEM with 10% heat-inactivated fetal bovine

serum (HyClone), penicillin (100 U/ml), streptomycin (100 μg/ml), nonessential amino acids (0.1 mM), and L-glutamine (2 mM) (Life Technologies) in a humidified incubator with 5% CO_2.

RNA isolation, reverse transcription and real-time PCR

Total RNA was isolated from cells or tissues by using Trizol reagent (Life Technologies) according to the manufacturer's protocol. In brief, Complementary DNA was produced from cellular RNA (1 μg) using a SuperScript II RNase H-Reverse Transcriptase Kit (Life Technologies). Real-time PCR reactions were performed in 10-μl quantities of diluted cDNA sample, primers (100 nM), and a SYBR Green PCR Master Mix containing nucleotides, AmpliTaq Gold DNA polymerase, and optimized buffer components (Applied Biosystems). Reactions were assayed using an Applied Biosystems Step-One-plus real-time PCR system. The primer pairs used are shown in Table 1.

Western blotting

Cells and tissues were lysed in lysis buffer (50 mM Tris [pH 7.5], 150 mM NaCl, 1% Tx-100) supplemented with protease- and phosphatase-inhibitors (1 mM PMSF, 10 μg/ml Leupeptin, 50 μg/ml TLCK, 50 μg/ml TPCK, 1 μg/ml Aprotinin, 1 mM NaF, 5

mM NaPPi, and 10 mM Na_3VO_4). The cell lysates were cleared via two 20 min spins at 13,200 rpm, 4°C. Protein concentrations were determined by Bio-Rad Protein Assay (Bio-Rad). Samples corresponded to 20 μg of protein which were used for Western blotting as described previously [1]. The antibodies used were: Mouse-Anti-GNMT monoclonal antibody [2]; Goat-Anti-vimentin polyclonal antibody (Santa Cruz); Rabbit-Anti-N-cadherin polyclonal antibody (Abcam); Rat-Anti-E-cadherin monoclonal antibody (Abcam); Mouse-Anti-GAPDH (Millipore).

Knock down experiment

Lentivirus production, infection and stable cell selection were carried out according to the protocol published on the web site of RNAi Core (http://rnai.genmed.sinica.edu.tw/). For lentivirus preparation, HEK293T cells were cotransfected with a packaging plasmid-pCMV-ΔR8.91, a VSV-G envelope expressing plasmid-pMD.G and the lentiviral constructs by using TurboFect™ Reagent (Fermentas). All transfections were performed according to the manufacturers' instructions. Supernatant containing lentiviruses was harvested according to the protocol published on the web site (http://rnai.genmed.sinica.edu.tw/). Ymac-1 cells were infected with pseudotyped lentivirus in medium containing polybrene (8 μg/ml). Lentivirus-infected Ymac-1 cells were selected and grown in

complete DMEM supplemented with puromycin (1 μg/ml, Sigma-Aldrich).

Microarray analysis

Total RNA was extracted as described previously. The RNA quality was accessed using Agilent 2100 Bioanalyzer (Agilent Technologies). Fluorescent antisense RNA (aRNA) targets were prepared from 1 μg total RNA samples using OneArray® Amino Allyl aRNA Amplification Kit (Phalanx Biotech Group) and Cy5 dyes (Amersham Pharmacia). Fluorescent targets were hybridized to the Mouse Whole Genome OneArray® with Phalanx hybridization buffer using Phalanx Hybridization System. The detailed descriptions of the gene array list are available from http://www.phalanx.com.tw/attachment/download/MOA/MOA2_probeSequence.zip. After 16 hrs hybridization at 50°C, non-specific binding targets were washed away by three different washing steps (Wash I, 42°C 5 mins; Wash II, 42°C 5 mins, 25°C 5 mins; Wash III, rinse 20 times), and the slides were dried by centrifugation and scanned by an Axon 4000B scanner (Molecular Devices). The Cy5 fluorescent intensities of each spot were analyzed by GenePix 4.1 software (Molecular Devices). The signal intensity of each spot was loaded into Rosetta Resolver System® (Rosetta Biosoftware) to process data analysis. The error model of Rosetta Resolver System® could remove both systematic and random errors form the data. We filtered out spots

that the flag is less than 0. Spots that passed the criteria were normalized by 50% media scaling normalization method. The technical repeat data was tested by Pearson correlation coefficient calculation to check the reproducibility (R value >0.975). Normalized spot intensities were transformed to gene expression log2 ratios between the control and treatment groups. The microarray data are available at GSE88827. The spots with log2 ratio ≥2 or log2 ratio ≤-2 and *P* value <0.05 are tested for further analysis. Genes that progressively up-regulated or down-regulated from normal to HCC to SHC were defined as the difference of Ymac-1 to HCC and HCC to WT mice liver; both were higher than 4-folds. Differentially expressed genes were annotated and pathway enrichment analysis was performed by using DAVID Bioinformatics online tools (Database for Annotation, Visualization and Integrated Discovery; http://david.abcc.ncifcrf.gov/) [3].

Supplementary Fig 1

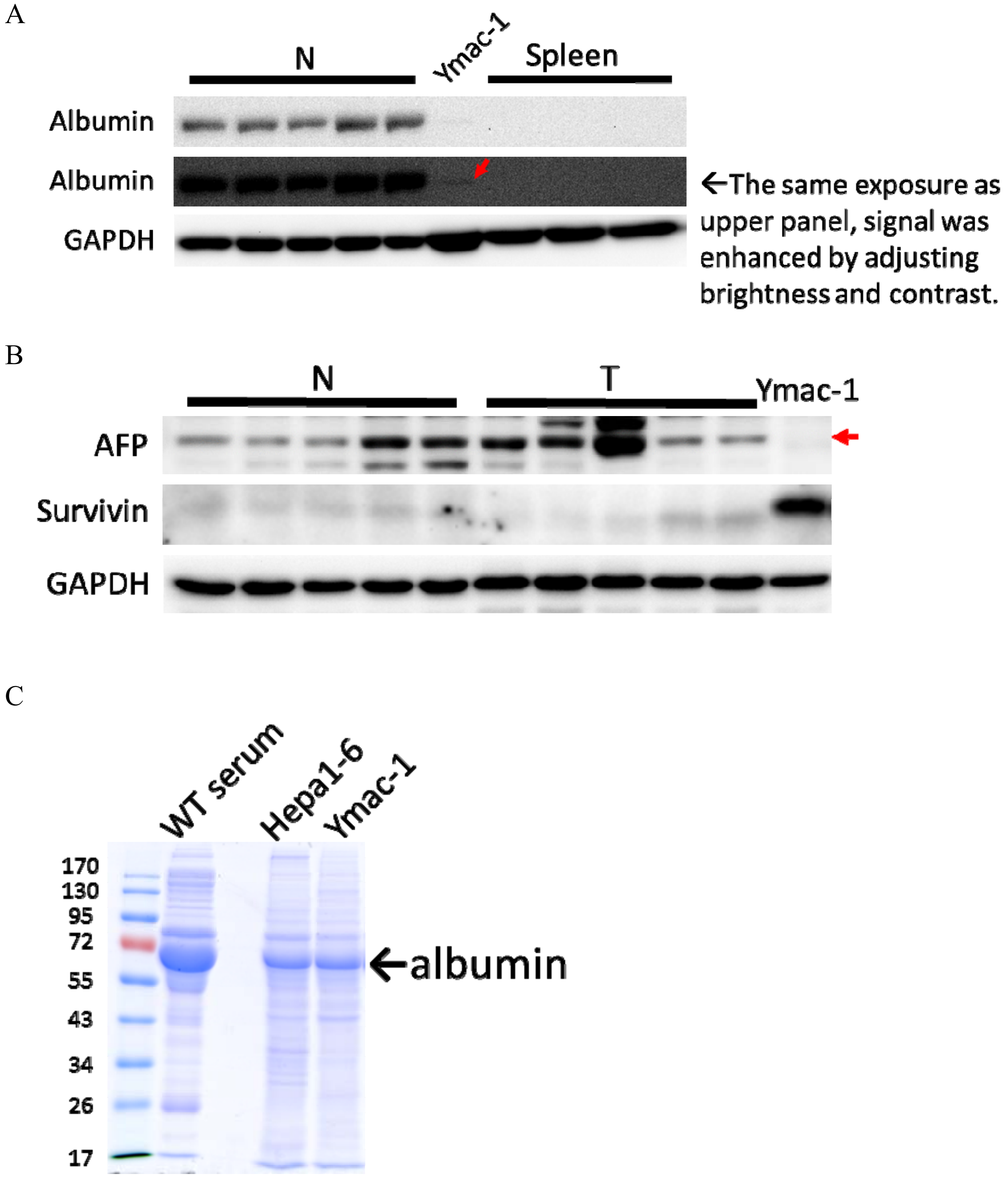

Supplementary Fig 1. SDS-PAGE analysis of secretory proteins from Ymac-1 cells cultured in serum free medium. (A) Western blot of analyses of Albumin in normal liver (N, n=5), Ymac-1 cell, and normal spleen (n=3). The protein amount is 20μg/lane for normal liver lysate and 200μg/lane for Ymac-1 and normal spleen

lysates. No Albumin signal was observed in spleen, while a vague band can still be seen in Ymac-1. We tried to enhance signal by adjust brightness and contrast (middle panel), but the signal was still weak (red arrow). Nevertheless, the result was consist with the qPCR data showed in Fig 1C. (B) Western blot of analyses of AFP and Survivin in normal liver (N, n=5), HCC tissues from Gnmt-/- mice (T, n=5) and Ymac-1 cells (correlated with Fig 1E and F). (C) Twenty uL of wild-type mouse serum and the culture supernatant from Hepa1-6 cells, an ordinary mouse HCC cell line, were also loaded next to Ymac-1 for comparison.

Supplementary Fig 2

A

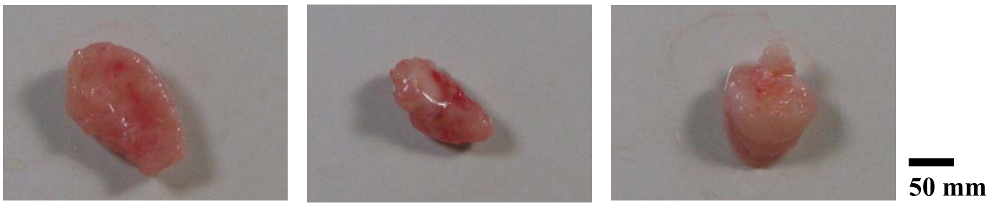

B

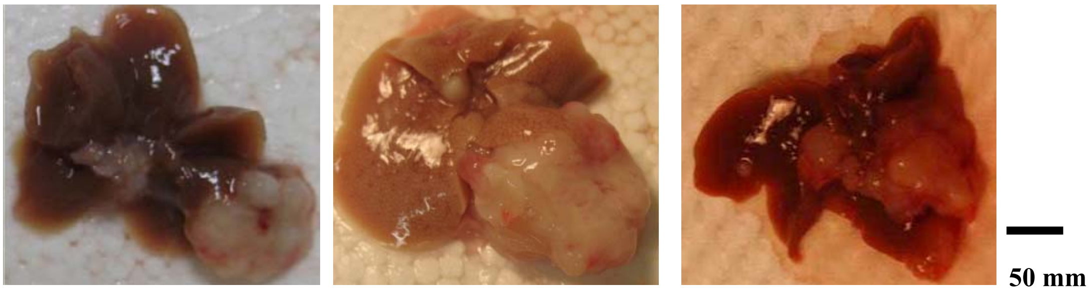

Supplementary Fig 2. Gross tumor pictures of Ymac-1 allograft tumors described in Fig 2. (A) Allograft tumors formed from subcutaneous inoculation. (B) Allograft tumors formed from intrahepatic inoculation. Bar: 50mm.

Supplementary Fig 3

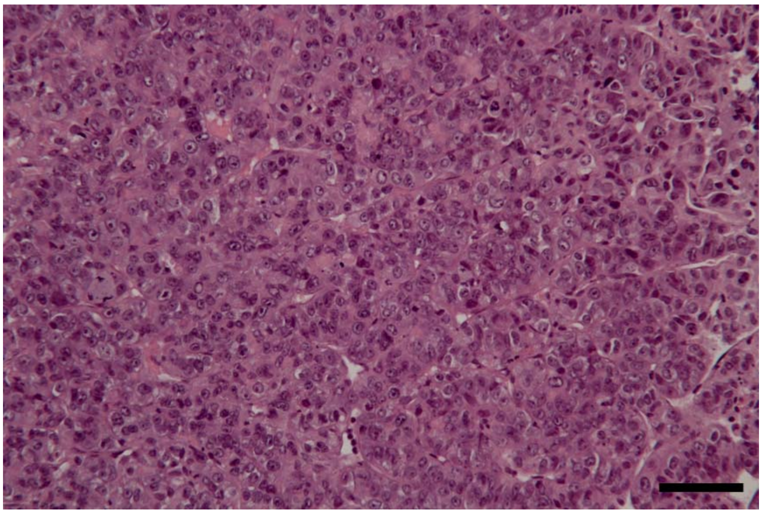

Supplementary Fig 3. H&E staining of allograft tumor formed from Hepa1-6 cell.

Hepa1-6 cells displayed typical HCC morphological features. Bar: 50μm.

Supplementary Fig 4

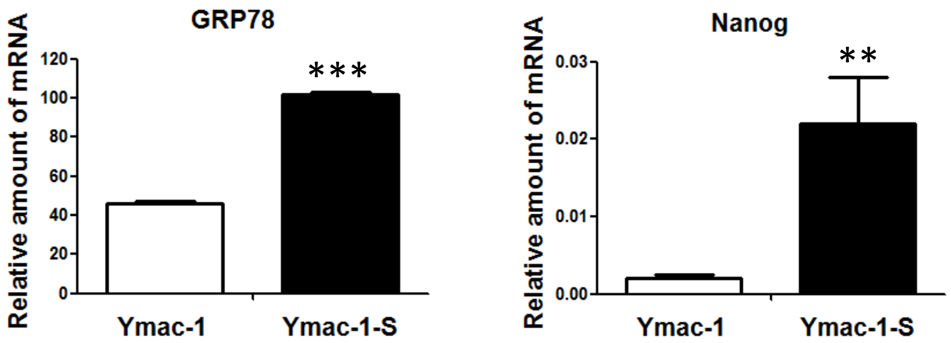

Supplementary Fig 4. The expression of Grp78 and Nanog in non-sphere (Ymac-1) and sphere (Ymac-1-S) cells.

Supplementary Fig 5

A

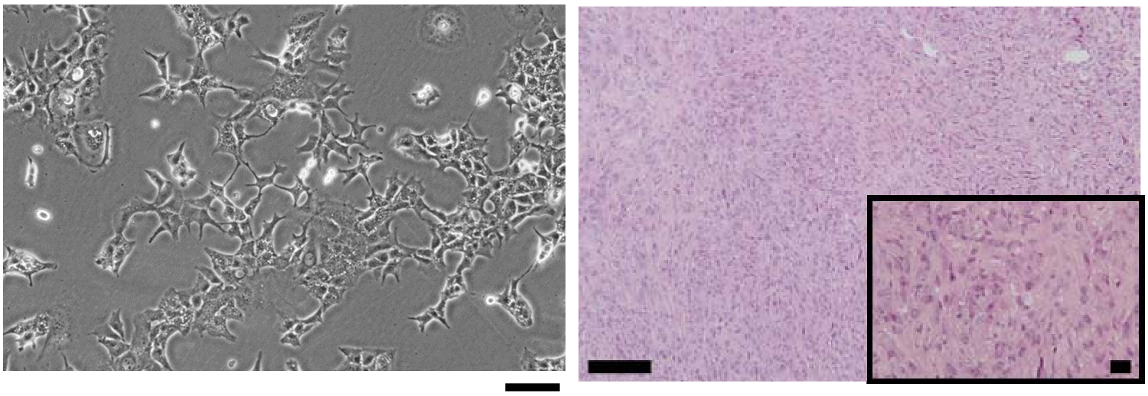

B

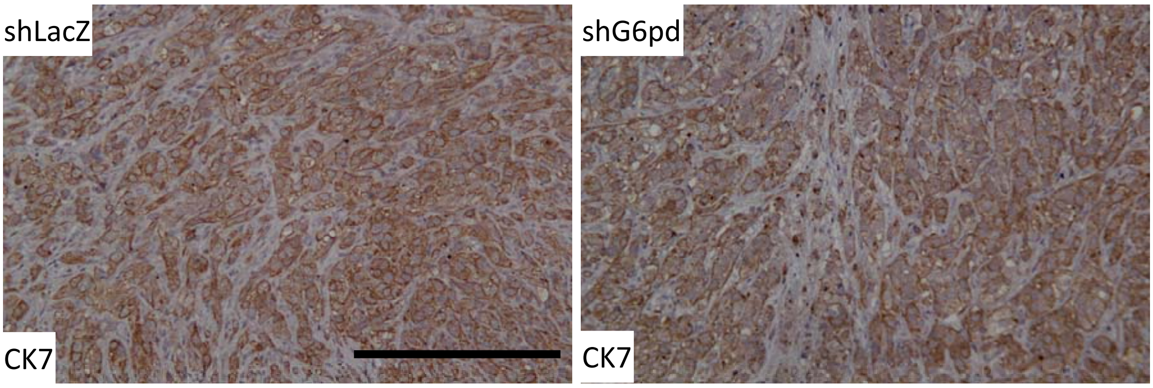

C

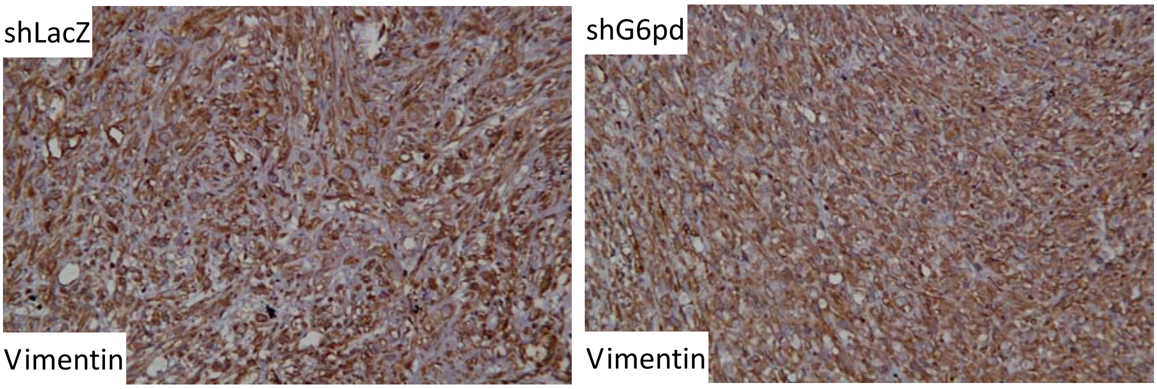

D

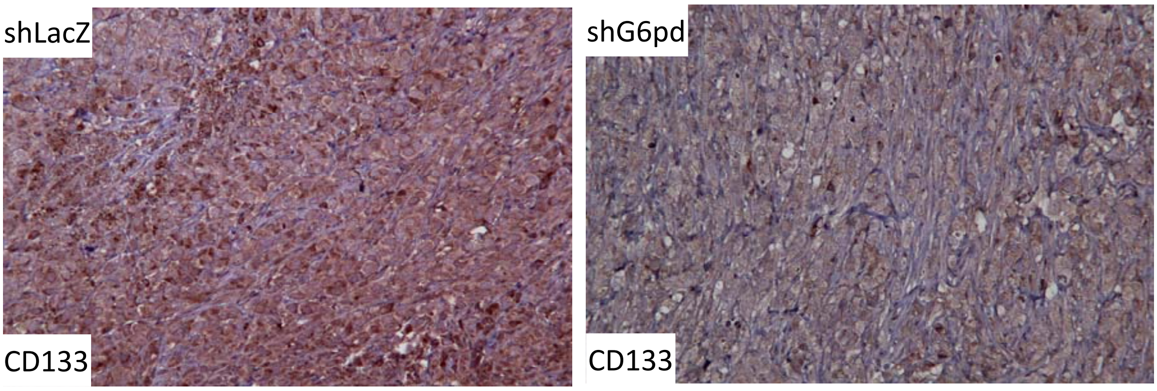

Supplementary Fig 5. Cell morphology and H&E staining of allograft tumor formed from G6pd-knock down Ymac-1 cell. (A) Left panel, an elongated, irregular fibroblastoid morphology was observed in G6pd knocked down Ymac-1 cell. Bar: 50μm. Right panel, a sarcomatoid appearance of allograft tumors formed from G6pd knock down Ymac-1 cells was observed. Bars: 100μm for lower magnification, 25μm for inset. (B-D) IHC staining of CK7 (B), and Vimentin (C) and CD133 (D) in Ymac-1 allograft tumor expressed of shG6pd (right panel) or control shRNA (shLacZ, left panel). Bars: 250μm.

Supplementary Fig 6

A

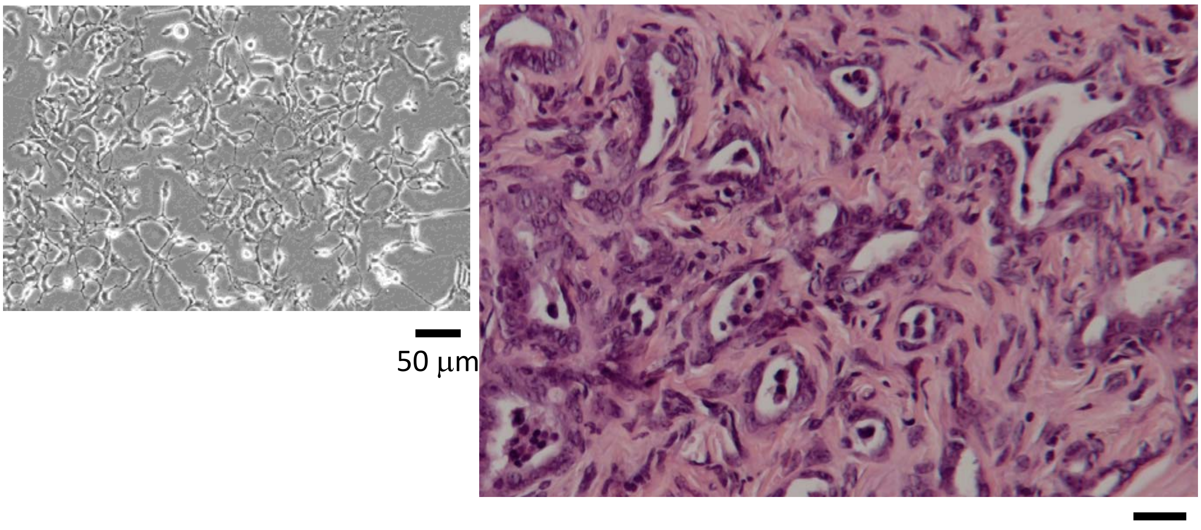

B

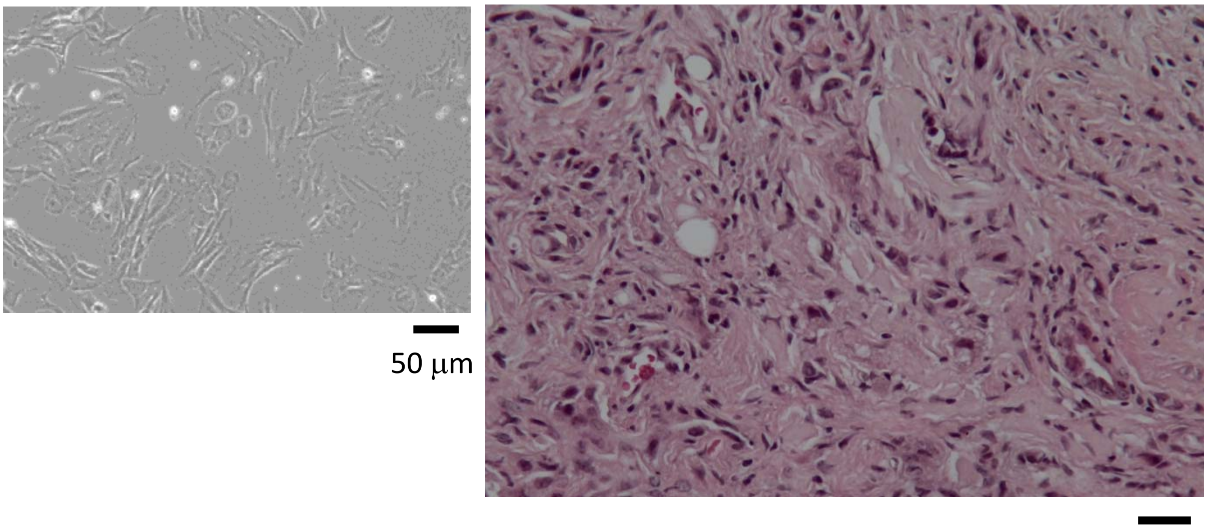

Supplementary Fig 6. Cell morphology and H&E staining of allograft tumors of Ymac-4 and Ymac-5 cells. (A) Cell morphology and pathology image of Ymac-4 cells. (B) Cell morphology and pathology image of Ymac-5 cells. Bars: 50μm for cell image, 25μm for H&E.

Supplementary Table1: List of genes up- or down-regulated in normal mice liver, Gnmt-/- mice liver tumor, and Ymac-1 cells.

				Normalize Value		
Group	Entrez Gene	Gene symbol	Description	Normal liver	Gnmt-/- HCC	Ymac-1
Pattern I -Up	16852	Lgals1	lectin, galactose binding,	1077.78381	9607.65625	64393.375
	20200	S100a6	S100 calcium binding pr	539.55249	2387.40283	53889.7773
	12306	Anxa2	annexin A2	1535.88647	13267.5	52868.0938
	22352	Vim	vimentin	811.868286	5942.52637	37270.2812
	18103|433968	Nme2|Gm5566	non-metastatic cells 2, pr	6896.01758	14253.5225	30957.0566
	27279	Tnfrsf12a	tumor necrosis factor rec	1926.28662	11701.293	29715.7305
	18746	Pkm2	pyruvate kinase, muscle	309.031097	2151.33154	29111.5449
	20250	Scd2	stearoyl-Coenzyme A de	335.545837	8834.62012	28634.9746
	16691	Krt8	keratin 8	1859.44617	10538.3818	28087.5781
	20296	Ccl2	chemokine (C-C motif) l	50	235.515152	27796.8398
	18858	Pmp22	peripheral myelin protein	155.541321	577.748108	26863.7969
	13014	Cstb	cystatin B	3541.21973	11011.9102	24834.2617
	70350	Basp1	brain abundant, membran	160.786591	1246.84314	24333.0762
	21346	Tagln2	transgelin 2	559.722534	5237.14258	20616.1777
	12389	Cav1	caveolin 1, caveolae prot	145.38504	566.33136	20466.0312
	66395	Ahnak	AHNAK nucleoprotein (	324.556244	1399.74292	18756.3184
	27053	Asns	asparagine synthetase	50	383.986938	18739.9297
	13730	Emp1	epithelial membrane prot	50	959.952515	18560.4023
	59069	Tpm3	tropomyosin 3, gamma	2706.69751	6296.23535	18463.0996
	22381	Wbp5	WW domain binding pro	965.945801	2672.04248	16818.7109
	11745	Anxa3	annexin A3	51.7541656	313.930115	16253.9043
	11674	Aldoa	aldolase A, fructose-bisp	1807.84082	6093.19141	15920.3828
	19240	Tmsb10	thymosin, beta 10	501.775726	2800.68018	15902.1641
	12484	Cd24a	CD24a antigen	69.8758087	895.444214	15842.7949
	20753	Sprr1a	small proline-rich protein	50	297.15155	15682.4629
	665433	Hist1h2ao	histone cluster 1, H2ao	604.507812	2907.31152	15506.041
	14115	Fbln2	fibulin 2	50	235.638229	13920.792
	16765|111186	Stmn1|Stmn1-rs1	stathmin 1|stathmin 1, re	594.555908	2115.67651	13792.0547
	59090	Midn	midnolin	2676.68335	6335.41895	13669.8711
	74107	Cep55	centrosomal protein 55	136.957092	1571.54492	13527.6875
	11974	Atp6v0e	ATPase, H+ transporting	1568.11377	3683.10449	13346.9824
	98238	Lrrc59	leucine rich repeat contai	1523.95898	3946.55469	13343.8711
	18787	Serpine1	serine (or cysteine) pepti	50	4672.50732	13070.4697
	26425	Nubp1	nucleotide binding protei	3014.36938	6348.03711	13026.9678
	192176	Flna	filamin, alpha	600.837219	2350.52808	12805.2734
	55989	Nop58	NOP58 ribonucleoprotein	1500.05981	3836.02295	12540.7256
	67511	Tmed9	transmembrane emp24 pr	1848.96045	5285.26562	12270.3467
	67951	Tubb6	tubulin, beta 6 class V	531.693359	1973.99817	12187.1494
	107435	Hat1	histone aminotransferase	735.891724	1537.66235	12117.2959
	74122	Tmem43	transmembrane protein 4	536.894226	2434.84668	11973.9238
	12827	Col4a2	collagen, type IV, alpha 2	524.735535	3776.44775	11919.1934
	23886	Gdf15	growth differentiation fac	973.188721	3298.41895	11716.8203
	13681	Eif4a1	eukaryotic translation ini	2403.36621	5564.9834	11705.7266
	227737	Fam129b	family with sequence sim	171.386475	1793.62158	11632.1641
	66286	Sec11c	SEC11 homolog C (S. ce	849.182495	2839.50977	11601.6895
	21807	Tsc22d1	TSC22 domain family, m	1028.87329	2232.67651	11503.3672
	69639	Exosc8	exosome component 8	1467.06091	4938.36816	11375.9375
	106957	Slc39a6	solute carrier family 39 (	562.672302	1294.2334	11248.0527
	12876	Cpe	carboxypeptidase E	152.079834	5403.36914	11184.418
	60527	Fads3	fatty acid desaturase 3	50	447.311127	11084.4326
	12615	Cenpa	centromere protein A	413.998474	1408.41663	10645.7695
	16573	Kif5b	kinesin family member 5	2261.63916	5192.20947	10636.0723
	353172	Gars	glycyl-tRNA synthetase	1990.03638	4482.01562	10486.8301
	52398	Sept11	septin 11	1302.13013	4382.03467	10142.5869
	83397	Akap12	A kinase (PRKA) anchor	115.453186	1187.66833	10120.6426
	15374	Hn1	hematological and neuro	486.694153	2303.95142	10013.3086
	12070	Ngfrap1	nerve growth factor recep	50	205.08316	9855.21191

17254	Slc3a2	solute carrier family 3 (a	1200.31836	2899.13721	9648.21094
13427	Dync1i2	dynein cytoplasmic 1 inte	1258.60571	2677.33228	9396.61328
16905	Lmna	lamin A	1113.07495	2698.05933	9317.54492
20195|100502897	S100a11|Gm12854	S100 calcium binding pr	50	448.609863	9197.0625
14225	Fkbp1a	FK506 binding protein 1a	1430.53723	3210.6936	9054.07812
224023	Klhl22	kelch-like 22 (Drosophila	639.280945	1305.61621	8950.11133
22142	Tuba1a	tubulin, alpha 1A	63.2205925	381.243805	8825.24219
70769	Nolc1	nucleolar and coiled-bod	573.360901	1220.60864	8783.75977
73690	Glipr1	GLI pathogenesis-related	153.982468	777.605286	8781.45703
21858	Timp2	tissue inhibitor of metallo	736.78833	2730.67969	8770.52344
97064	Wwtr1	WW domain containing t	919.044006	3573.53613	8683.56445
108143	Taf9	TAF9 RNA polymerase	1291.54443	3174.24756	8667.17969
67134	Nop56	NOP56 ribonucleoprotein	322.634613	871.321899	8645.73828
22156	Tuft1	tuftelin 1	150.226334	1582.65186	8582.07324
12476	Cd151	CD151 antigen	850.487793	1727.76807	8563.48828
229731	Slc25a24	solute carrier family 25 (	131.781525	1819.74304	8352.09375
11821	Aprt	adenine phosphoribosyl t	840.23645	2023.28687	8236.21094
22038	Plscr1	phospholipid scramblase	455.122681	1852.51636	8214.43262
56433	Vps29	vacuolar protein sorting 2	1771.74023	3796.46582	8177.14014
68219	Nudt21	nudix (nucleoside diphos	1032.67383	2280.45581	8034.29199
107272	Psat1	phosphoserine aminotran	50	194.445892	7965.07812
54673	Sh3glb1	SH3-domain GRB2-like	708.141296	1562.10449	7927.15576
67834	Idh3a	isocitrate dehydrogenase	941.628296	2098.50464	7908.69873
16669	Krt19	keratin 19	50	110.246124	7890.83154
14219	Ctgf	connective tissue growth	220.759598	728.07605	7814.63428
17698	Msn	moesin	1158.44324	3148.92285	7758.78125
15382|434858	Hnrnpa1|Gm5643	heterogeneous nuclear rib	1054.39478	2116.25635	7651.09961
13685	Eif4ebp1	eukaryotic translation ini	1064.6825	3469.55859	7622.96484
16779	Lamb2	laminin, beta 2	302.649963	1458.33069	7592.29199
53331	Stx7	syntaxin 7	913.241089	2385.06836	7469.47559
58523	Elp2	elongation protein 2 hom	1345.78906	3227.16943	7445.15039
11739	Slc25a4	solute carrier family 25 (	111.722397	1505.24268	7270.80078
67938	Myl12b	myosin, light chain 12B,	248.061646	1087.87415	7085.21973
16952	Anxa1	annexin A1	58.518692	496.560303	7077.87109
66054	Cndp2	CNDP dipeptidase 2 (me	909.611694	1966.39844	6953.17139
11544	Adprh	ADP-ribosylarginine hyd	759.36792	2136.56494	6914.64453
68816	Ppil1	peptidylprolyl isomerase	424.918365	896.979187	6906.57129
20641	Snrpd1	small nuclear ribonucleo	299.163147	629.432007	6874.32715
11632	Aip	aryl-hydrocarbon recepto	948.601929	1898.11694	6823.59277
67041	Oxct1	3-oxoacid CoA transfera	189.308502	922.973206	6722.25586
68480	1110007C09Rik	RIKEN cDNA 1110007C	310.624817	639.299683	6721.26807
98878	Ehd4	EH-domain containing 4	291.283325	845.097229	6600.0752
14609	Gja1	gap junction protein, alp	602.874512	2018.24353	6397.23486
67896	Ccdc80	coiled-coil domain conta	522.877258	2090.4917	6373.88086
66409	Rsl1d1	ribosomal L1 domain con	742.421753	1699.27698	6291.18652
14062	F2r	coagulation factor II (thr	335.90387	2485.33105	6177.80371
16573	Kif5b	kinesin family member 5	929.612183	2267.24072	6167.84863
15277	Hk2	hexokinase 2	161.165222	2946.37476	6076.60938
12527	Cd9	CD9 antigen	317.733093	2288.47314	6043.69971
21859	Timp3	tissue inhibitor of metallo	768.702026	1844.05212	6018.47363
16007	Cyr61	cysteine rich protein 61	114.486832	458.519958	6013.0708
67116	Cuedc2	CUE domain containing	839.997314	1755.56567	5982.81006
11983	Atpif1	ATPase inhibitory factor	553.899048	2361.66211	5845.13037
228359	Arhgap1	Rho GTPase activating p	735.1297	1775.08887	5838.66895
16400	Itga3	integrin alpha 3	88.6596451	432.78479	5826.73389
56449	Csda	cold shock domain protei	430.187408	1127.72253	5819.14648
20708	Serpinb6b	serine (or cysteine) pepti	50	989.135376	5805.64062
27214	Dbf4	DBF4 homolog (S. cerev	62.8023643	248.895554	5711.30078
227620	Uap1l1	UDP-N-acteylglucosamin	227.61441	2700.13037	5639.125
12798	Cnn2	calponin 2	190.922791	600.762756	5628.02637
19041	Ppl	periplakin	436.253296	2294.3667	5547.37939

12336	Capns1	calpain, small subunit 1	610.480225	1458.68579	5533.05518
121022	Mrps6	mitochondrial ribosomal	801.65332	1973.47192	5530.93848
73916	Ift57	intraflagellar transport 57	135.89093	542.729614	5503.9873
104458	Rars	arginyl-tRNA synthetase	412.474487	937.113281	5501.94824
16998	Ltbp3	latent transforming grow	108.305283	1172.04138	5493.8501
12334	Capn2	calpain 2	815.435852	2047.18713	5425.18945
330171	Kctd10	potassium channel tetram	499.784393	1269.69653	5282.41016
23789	Coro1b	coronin, actin binding pr	1107.66333	2288.16455	5250.55078
76453	Prss23	protease, serine, 23	288.602875	1322.79785	5178.68359
14156	Fen1	flap structure specific en	166.096832	344.570068	5175.69824
69051	Pycr2	pyrroline-5-carboxylate r	219.927887	715.452515	5169.22168
140559	Igsf8	immunoglobulin superfam	186.165466	759.520996	5120.37109
11848	Rhoa	ras homolog gene family	629.519165	1832.70886	5082.56396
66059	Krtcap2	keratinocyte associated p	1077.90259	2309.8125	5076.21924
326618	Tpm4	tropomyosin 4	236.012253	1126.36865	5057.08984
21873	Tjp2	tight junction protein 2	563.20459	1677.70679	5041.87305
76522	Naa38	N(alpha)-acetyltransferas	244.709503	701.131348	5011.31006
16881	Lig1	ligase I, DNA, ATP-depe	279.751953	669.433105	5001.83008
21335	Tacc3	transforming, acidic coile	50	242.692017	4993.16992
12315	Calm3	calmodulin 3	1065.02002	2218.927	4982.55127
51792	Ppp2r1a	protein phosphatase 2 (fo	733.401367	1919.34619	4979.83691
56434	Tspan3	tetraspanin 3	57.970562	265.330322	4934.81445
21857	Timp1	tissue inhibitor of metallo	50	802.012024	4932.34961
11746	Anxa4	annexin A4	624.657227	1260.4187	4931.77051
329384	Ptrh1	peptidyl-tRNA hydrolase	54.936821	515.306702	4907.58203
17191	Mbd2	methyl-CpG binding dom	678.139771	1522.83337	4867.20166
67203	Nde1	nuclear distribution gene	1072.01575	2171.18164	4863.49609
107373	Fam111a	family with sequence sim	87.3495178	194.689209	4828.21191
74202	Fblim1	filamin binding LIM prot	50	477.610138	4823.5957
51797	Ctps	cytidine 5'-triphosphate s	140.622925	757.008423	4735.09082
245446	Slitrk4	SLIT and NTRK-like fam	50	113.7136	4725.43994
94352	Loxl2	lysyl oxidase-like 2	246.965393	1832.06665	4721.87793
21973	Top2a	topoisomerase (DNA) II	50	147.809723	4649.19922
13197	Gadd45a	growth arrest and DNA-d	356.157837	1937.36121	4616.05762
18701	Pigf	phosphatidylinositol glyc	812.600159	1704.7915	4599.24219
15469	Prmt1	protein arginine N-methy	318.425415	955.852661	4568.04297
20719	Serpinb6a	serine (or cysteine) pepti	158.296234	1253.90967	4563.30176
13007	Csrp1	cysteine and glycine-rich	371.828735	919.89624	4539.37109
56405	Dusp14	dual specificity phosphat	50	176.699677	4501.55469
56200	Ddx21	DEAD (Asp-Glu-Ala-As	442.540985	914.316528	4494.58203
11853	Rhoc	ras homolog gene family	344.387238	859.844238	4490.48535
27357	Gyg	glycogenin	294.36142	1359.25098	4453.84863
76252	Atp6v0e2	ATPase, H+ transporting	176.901062	1621.92017	4434.22266
12585	Cdr2	cerebellar degeneration-r	97.5311584	859.821655	4432.04248
110829	Lims1	LIM and senescent cell a	400.11615	1039.55591	4420.16504
52009	Hn1l	hematological and neuro	308.831482	701.663208	4380.43359
12334	Capn2	calpain 2	242.29834	561.248291	4319.71289
69065	Chac1	ChaC, cation transport re	97.4313507	306.136444	4310.09521
73710	Tubb2b	tubulin, beta 2B class IIB	50	156.638977	4303.34473
213539	Bag2	BCL2-associated athanog	60.974205	263.557678	4283.22266
22319	Vamp3	vesicle-associated memb	425.455383	917.549194	4208.35205
94181	Nans	N-acetylneuraminic acid	240.051956	566.020142	4188.82373
72050	Kdelc1	KDEL (Lys-Asp-Glu-Le	295.481445	882.809326	4187.71436
12018	Bak1	BCL2-antagonist/killer 1	699.06543	1948.82178	4180.9668
22629	Ywhah	tyrosine 3-monooxygena	232.355927	622.754517	4165.93604
14594	Ggta1	glycoprotein galactosyltr	207.056274	586.943787	4124.30713
16582	Kifc3	kinesin family member C	399.941895	1310.24231	4119.15723
223921	Aaas	achalasia, adrenocortical	394.736206	1303.47424	4066.27148
104445	Cdc42ep1	CDC42 effector protein (	437.848602	1389.30371	4043.57495
67849	Cdca5	cell division cycle associ	50	121.48748	4025.72729
12444	Ccnd2	cyclin D2	166.694077	356.310822	3992.84863

233406	Prc1	protein regulator of cytol	50	292.76593	3921.84473
114774	Pawr	PRKC, apoptosis, WT1,	443.347351	1093.82019	3910.97949
17684	Cited2	Cbp/p300-interacting tra	646.623718	1754.91504	3906.08228
11886	Asah1	N-acylsphingosine amid	625.598267	1882.51685	3903.07031
27280	Phlda3	pleckstrin homology-like	50	166.922546	3902.19116
12406	Serpinh1	serine (or cysteine) pepti	75.1258392	351.019775	3891.22217
94242	Tinagl1	tubulointerstitial nephriti	169.174927	378.676086	3866.20654
20620	Plk2	polo-like kinase 2 (Dros	333.662231	1479.54443	3840.18896
13043	Cttn	cortactin	545.665894	1668.61157	3804.27197
226351	Tmem185b	transmembrane protein 1	701.307068	1466.85571	3785.64868
76205	Stard3nl	STARD3 N-terminal like	327.062439	743.271484	3764.7627
66578	2610039C10Rik	RIKEN cDNA 2610039C	130.232178	426.776489	3764.4292
67703	Kirrel3	kin of IRRE like 3 (Dros	237.610703	496.52774	3759.18384
99480	Dnttip2	deoxynucleotidyltransfer	623.446899	1524.00903	3695.8457
72621	Pdzd11	PDZ domain containing	259.089264	528.183472	3657.60986
56367	Scoc	short coiled-coil protein	719.602966	1490.68115	3637.29102
11799	Birc5	baculoviral IAP repeat-c	50	111.469856	3628.4043
66665	5730528L13Rik	RIKEN cDNA 5730528L	50	617.363037	3616.58252
19285	Ptrf	polymerase I and transcri	249.028015	507.041931	3601.07471
100678	Psph	phosphoserine phosphata	50	210.153503	3598.1499
76895	Bicd2	bicaudal D homolog 2 (D	781.694031	1606.16736	3586.08154
223593	E430025E21Rik	RIKEN cDNA E430025E	492.984985	1238.45532	3577.15723
81703	Jdp2	Jun dimerization protein	114.456726	606.783813	3571.44678
15937	Ier3	immediate early response	50	753.531006	3468.35107
68151	Wls	wntless homolog (Droso	70.7471161	262.629639	3441.99463
106878	2010002N04Rik	RIKEN cDNA 2010002N	59.2410889	380.052612	3436.33374
64143	Ralb	v-ral simian leukemia vi	342.573334	1023.80865	3427.34668
17219	Mcm6	minichromosome mainte	108.374992	225.208923	3423.85303
20405	Sh3gl1	SH3-domain GRB2-like	480.587097	1102.74414	3404.99512
77125	Il33	interleukin 33	50	367.441833	3401.31567
29856	Smtn	smoothelin	145.763641	525.038574	3319.87012
68682	Slc44a2	solute carrier family 44,	458.913696	1023.34888	3318.73779
108013	Celf4	CUGBP, Elav-like famil	83.7882385	236.463013	3310.00073
66128|170755	Mrps36|Sgk3	mitochondrial ribosomal	414.790588	1072.81873	3306.20801
12428	Ccna2	cyclin A2	50	211.244247	3305.70703
11799	Birc5	baculoviral IAP repeat-c	50	127.297729	3305.62158
68201	Ccdc34	coiled-coil domain conta	50	229.850616	3291.53198
72657	2700094K13Rik	RIKEN cDNA 2700094K	201.348419	559.199768	3285.77881
75416	Nop14	NOP14 nucleolar protein	700.320129	1489.89868	3249.87012
110033	Kif22	kinesin family member 2	65.4970856	272.958496	3232.24268
17427	Mns1	meiosis-specific nuclear	50	111.865982	3219.37451
237107	Gnl3l	guanine nucleotide bindi	459.640808	1103.89575	3202.1167
14793	Cdca3	cell division cycle associ	50	215.938293	3197.64697
20135	Rrm2	ribonucleotide reductase	50	447.213501	3187.52783
59003	Maea	macrophage erythroblast	496.357788	1390.72559	3139.84668
98053	Gtf2f1	general transcription fact	569.778992	1172.5238	3121.34521
12488	Cd2ap	CD2-associated protein	497.702759	1174.04468	3109.10254
66609	Cryzl1	crystallin, zeta (quinone	332.103363	982.450806	3092.72583
67306	Fam164a	family with sequence sin	76.4708252	365.964844	3085.34424
20677	Sox4	SRY-box containing gen	50.6182938	434.966309	3084.74268
223752	Gramd4	GRAM domain containin	649.717651	1307.49927	3070.08008
17873	Gadd45b	growth arrest and DNA-d	629.03595	1372.35266	3069.88916
101772	Ano1	anoctamin 1, calcium act	50	113.508469	3052.36719
107094	Rrp12	ribosomal RNA processi	433.52533	926.330322	3049.21851
70123	2210013O21Rik	RIKEN cDNA 2210013O	110.860596	690.301636	3048.49561
56150	Mad2l1	MAD2 mitotic arrest def	50	153.013062	3044.81128
14700	Gng10	guanine nucleotide bindi	654.135986	1337.09082	3010.39795
52033	Pbk	PDZ binding kinase	50	187.635437	3008.00879
56297	Arl6	ADP-ribosylation factor-	153.668793	362.83551	2989.49878
11854	Rhod	ras homolog gene family	347.809082	794.912964	2971.20752
15979	Ifngr1	interferon gamma recept	164.377991	705.697998	2956.89307

66964	Golt1b	golgi transport 1 homolo	195.754608	796.928955	2939.93945
66468	Ska1	spindle and kinetochore a	50	136.490555	2914.03809
13803	Enc1	ectodermal-neural cortex	422.36145	992.182617	2908.67773
100045148|14000	LOC100045148|D	ribonuclease 3-like|drosh	264.369385	903.145935	2889.31836
64540	Tspan4	tetraspanin 4	317.145386	719.35144	2884.73047
20775	Sqle	squalene epoxidase	582.262451	1287.65637	2873.75171
19255	Ptpn2	protein tyrosine phosphat	439.71637	951.117554	2840.28003
78372	Snrnp25	small nuclear ribonucleo	226.762115	567.178772	2808.1228
65106	Arl6ip5	ADP-ribosylation factor-	378.911682	1171.04114	2798.76758
72388	Ripk4	receptor-interacting serin	426.142944	1380.49438	2795.90186
226251	Ablim1	actin-binding LIM protei	230.627563	718.250854	2792.32959
98386	Lbr	lamin B receptor	622.589844	1258.36316	2789.94653
27279	Tnfrsf12a	tumor necrosis factor rec	132.747879	582.512878	2776.83667
22200	Uba3	ubiquitin-like modifier ac	482.00177	1111.48584	2771.92651
67678	Lsm3	LSM3 homolog, U6 sma	180.671448	424.44928	2715.02661
21929	Tnfaip3	tumor necrosis factor, alp	150.600204	936.3479	2710.80835
80286	Tusc3	tumor suppressor candida	93.9445343	222.543594	2710.54541
56772	Mllt11	myeloid/lymphoid or mix	85.660759	338.762665	2699.58984
67177	Cdt1	chromatin licensing and	97.1873856	324.587189	2669.0708
216829	Mmgt2	membrane magnesium tr	89.212532	447.694519	2668.47754
208092	Chmp6	charged multivesicular b	216.645416	458.773193	2632.43091
100608	Noc4l	nucleolar complex associ	304.996155	668.345215	2614.20361
69161	Manbal	mannosidase, beta A, lys	332.546936	769.346191	2594.66211
12400	Cbfb	core binding factor beta	456.204681	1271.23145	2575.91382
58859	Efemp2	epidermal growth factor-	64.2867584	200.464096	2566.09033
15200	Hbegf	heparin-binding EGF-like	50	210.043152	2549.2666
67249	Tbc1d19	TBC1 domain family, me	85.6259079	300.546875	2548.10815
67399	Pdlim7	PDZ and LIM domain 7	119.632309	530.144287	2543.67627
17755	Mtap1b	microtubule-associated p	50	103.565834	2534.07617
11603	Agrn	agrin	344.422089	833.995972	2530.54639
18105	Nqo2	NAD(P)H dehydrogenase	504.690674	1116.63953	2518.99609
74257	Tspan17	tetraspanin 17	78.4780121	273.36734	2518.60156
19108	Prkx	protein kinase, X-linked	378.224121	975.998291	2499.4707
16978	Lrrfip1	leucine rich repeat (in FL	341.86676	984.203613	2464.79932
14187	Akr1b8	aldo-keto reductase famil	151.431915	318.64679	2430.91016
11769	Ap1s1	adaptor protein complex	155.362305	370.518829	2430.81152
83554	Fstl3	follistatin-like 3	50	324.366516	2427.04346
21982	Tmem165	transmembrane protein 1	144.379059	413.724304	2410.80957
97165	Hmgb2	high mobility group box 2	61.3528214	176.689758	2397.87549
105348	Golm1	golgi membrane protein	50	425.948883	2394.99805
12286	Cacna1a	calcium channel, voltage	69.2579727	189.64151	2394.87646
20198	S100a4	S100 calcium binding pr	157.638794	394.734558	2380.04932
227700	Sh3glb2	SH3-domain GRB2-like	238.428162	598.292664	2344.8042
21985	Tpd52	tumor protein D52	167.376862	626.217773	2326.87939
74451	Pgs1	phosphatidylglycerophos	234.736969	567.850708	2323.58154
83396	Glis2	GLIS family zinc finger	50	274.415649	2314.58936
70235	Poc1a	POC1 centriolar protein	249.53653	541.031982	2312.78516
53605	Nap1l1	nucleosome assembly pro	216.331757	716.82196	2310.75098
52713	Ccdc59	coiled-coil domain conta	343.002655	815.535278	2306.7627
66441	Magohb	mago-nashi homolog B (	50	157.1129	2292.89844
226101	Myof	myoferlin	111.727158	249.34967	2289.16968
56233	Hdac7	histone deacetylase 7	223.31015	598.026672	2284.30737
29858	Pmm1	phosphomannomutase 1	280.239899	769.590942	2270.43164
12444	Ccnd2	cyclin D2	171.486267	382.045959	2235.73242
105298	Epdr1	ependymin related protei	50	171.840118	2211.43652
226419	Dyrk3	dual-specificity tyrosine-	100.400139	601.158875	2209.9165
19703	Renbp	renin binding protein	266.018555	1015.19727	2207.25439
20818	Srprb	signal recognition particl	381.889954	764.415894	2205.67188
79201	Tnfrsf23	tumor necrosis factor rec	75.2652512	164.046432	2203.53418
26417	Mapk3	mitogen-activated protein	218.169434	446.054871	2202.81592
65964	B230120H23Rik	RIKEN cDNA B230120I	184.776108	393.10199	2198.58594

27416	Abcc5	ATP-binding cassette, su	171.077545	923.670654	2182.6167
239273	Abcc4	ATP-binding cassette, su	122.596344	602.385376	2182.21094
22154	Tubb5	tubulin, beta 5 class I	161.822662	325.898621	2173.73975
67267	2900010M23Rik	RIKEN cDNA 2900010M	355.57959	772.924011	2159.6521
26362	Axl	AXL receptor tyrosine ki	206.359222	703.325562	2133.4436
216963	Git1	G protein-coupled recept	214.090118	688.912354	2128.66504
17345	Mki67	antigen identified by mo	50	226.961761	2120.0625
448850	Znhit3	zinc finger, HIT type 3	286.939453	644.921753	2116.87769
13872	Ercc3	excision repair cross-com	361.656586	929.465332	2083.23682
106795	Tcf19	transcription factor 19	50	126.07399	2071.31958
16477	Junb	Jun-B oncogene	263.563049	641.085022	2060.27856
67203	Nde1	nuclear distribution gene	220.849884	452.378662	2030.5387
20877	Aurkb	aurora kinase B	50	121.113998	2028.07544
73112	3110003A17Rik	RIKEN cDNA 3110003A	247.652939	608.315918	2025.92773
107449	Unc5b	unc-5 homolog B (C. ele	69.8457108	915.036621	2015.19214
17776	Mast2	microtubule associated se	290.252014	997.671753	2006.88379
19332	Rab20	RAB20, member RAS on	227.623932	863.781433	1994.03662
19376	Rab34	RAB34, member of RAS	63.0415802	397.259827	1992.23401
224105	Pak2	p21 protein (Cdc42/Rac)	377.805908	926.094116	1983.60193
16319	Incenp	inner centromere protein	50	182.961212	1980.02954
12927	Bcar1	breast cancer anti-estroge	138.929428	302.961823	1966.39697
17876	Myef2	myelin basic protein expr	50	106.178818	1947.35132
270685	Mthfd1l	methylenetetrahydrofolat	50	311.580261	1941.922
11845	Arf6	ADP-ribosylation factor	251.887482	635.96936	1937.34546
69692	Hddc2	HD domain containing 2	84.694397	402.349976	1933.70239
613262	BC029722	cDNA sequence BC0297	248.440277	629.10376	1904.08899
18767	Pkia	protein kinase inhibitor, a	50	126.437576	1902.52124
66120	Fkbp11	FK506 binding protein 1	145.559296	814.181396	1890.84253
12579	Cdkn2b	cyclin-dependent kinase	50	151.734146	1889.47205
18817	Plk1	polo-like kinase 1 (Droso	50	216.093903	1879.41528
67429	Nudcd1	NudC domain containing	200.934937	561.920288	1869.6394
67936	Wdr55	WD repeat domain 55	211.016769	438.303619	1865.34558
53886	Cdkl2	cyclin-dependent kinase-	251.827301	835.748779	1861.73535
66583	Exosc1	exosome component 1	213.019196	645.78186	1859.6073
218138	Gmds	GDP-mannose 4, 6-dehy	86.5526581	177.735229	1859.48071
66061	Tctex1d2	Tctex1 domain containin	142.241974	463.60022	1852.81885
100929	Tyw1	tRNA-yW synthesizing p	317.075684	773.352661	1837.05347
17215	Mcm3	minichromosome mainte	50	100.358665	1835.6123
192662	Arhgdia	Rho GDP dissociation in	209.73674	547.536804	1825.26953
56356	Gltp	glycolipid transfer protein	101.540756	417.077209	1816.50427
17876	Myef2	myelin basic protein expr	50	228.659424	1807.73584
12337	Capn5	calpain 5	153.115906	469.235016	1805.62085
72275	2200002D01Rik	RIKEN cDNA 2200002D	106.302856	692.184631	1802.82739
227522	Rpp38	ribonuclease P/MRP 38 s	202.519135	442.315765	1799.08069
20525	Slc2a1	solute carrier family 2 (fa	59.8034782	788.4505	1798.60583
16897	Llgl1	lethal giant larvae homol	319.905029	751.86731	1792.18066
227059	Slc39a10	solute carrier family 39 (	50	103.543198	1785.70947
104346	Gas8	growth arrest specific 8	107.373779	332.436035	1781.03101
18476	Pafah1b3	platelet-activating factor	50	381.286255	1778.95886
68475	Ssna1	Sjogren's syndrome nucle	231.329346	538.068115	1761.70776
15468	Prmt2	protein arginine N-methy	50	131.761154	1759.54529
18582	Pde6d	phosphodiesterase 6D, cG	145.763641	328.699768	1758.6792
53890	Sart3	squamous cell carcinoma	370.353821	781.675415	1755.58008
76273	Ndfip2	Nedd4 family interacting	307.412048	635.771362	1755.52905
319757	Smo	smoothened homolog (D	174.285553	696.239197	1754.4248
109711	Actn1	actinin, alpha 1	172.287872	585.797852	1726.31836
12380	Cast	calpastatin	191.445587	644.104004	1717.81934
208943	Myo5c	myosin VC	50	318.539276	1706.13574
103468	Nup107	nucleoporin 107	71.2255478	144.040909	1705.49316
13642	Efnb2	ephrin B2	77.8902664	184.83429	1700.30371
57373	D930014E17Rik	RIKEN cDNA D930014E	287.452759	608.030151	1685.88562

20018	Polr1d	polymerase (RNA) I poly	294.0224	591.890991	1678.42358
17869	Myc	myelocytomatosis oncog	50	578.578552	1663.73267
68948	1500011H22Rik	RIKEN cDNA 1500011H	50	138.749863	1656.44971
72729	Cdc42se2	CDC42 small effector 2	216.570969	491.808258	1656.41357
69787	Anxa13	annexin A13	50	151.315399	1647.5564
232566	Amn1	antagonist of mitotic exit	390.851746	789.423828	1646.68872
11987	Slc7a1	solute carrier family 7 (c	50	114.203094	1643.31348
110208	Pgd	phosphogluconate dehyd	117.625122	502.523315	1638.8667
19826	Rnps1	ribonucleic acid binding	50	131.475388	1626.06396
18034	Nfkb2	nuclear factor of kappa li	127.746567	537.837524	1618.03662
17089	Lyar	Ly1 antibody reactive clo	256.828613	540.95697	1616.25696
16452	Jak2	Janus kinase 2	149.942764	390.566803	1615.91357
21753	Tes	testis derived transcript	50	155.934448	1611.66406
77305	Wdr82	WD repeat domain conta	240.99295	508.827301	1607.13513
67936	Wdr55	WD repeat domain 55	273.340698	561.880615	1573.40869
105372	Utp15	UTP15, U3 small nucleo	284.753296	579.786743	1562.72095
68298	Ncapd2	non-SMC condensin I co	103.090103	308.574005	1559.80396
14825	Cxcl1	chemokine (C-X-C motif	158.15683	751.421692	1559.68237
56306	Fam60a	family with sequence sim	50	135.101303	1556.87402
12709	Ckb	creatine kinase, brain	58.6581039	161.037323	1549.03247
20617	Snca	synuclein, alpha	50	156.418274	1546.63818
93715|93702|9370	Pcdhga7|Pcdhgb5|P	protocadherin gamma sul	269.091888	641.669312	1535.505
66147	Necap2	NECAP endocytosis asso	253.10257	542.534363	1535.00537
232210	8430410A17Rik	RIKEN cDNA 8430410A	267.50769	603.687012	1534.2627
59015	Nup160	nucleoporin 160	98.123642	249.778336	1531.50525
19822	Rnf4	ring finger protein 4	178.11615	356.233002	1527.54175
384009	Glipr2	GLI pathogenesis-related	50	413.568695	1524.48853
107047	Psmg2	proteasome (prosome, ma	297.235168	663.44751	1524.41626
56070	Tcerg1	transcription elongation r	221.207916	495.940643	1523.3562
94352	Loxl2	lysyl oxidase-like 2	50	148.757568	1520.3031
12305	Ddr1	discoidin domain recepto	50	407.926819	1516.85718
78749	Filip1l	filamin A interacting pro	115.244072	356.551331	1507.43958
67429	Nudcd1	NudC domain containing	81.4467926	184.184937	1505.88672
106672	AI413582	expressed sequence AI41	169.344452	677.969604	1504.68872
101631	Pwwp2b	PWWP domain containin	80.8289566	199.782196	1499.52881
68262	Agpat4	1-acylglycerol-3-phospha	102.1586	211.695541	1494.06335
56693	Crtap	cartilage associated prote	161.025818	626.613892	1491.18433
67629	Spc24	SPC24, NDC80 kinetoch	109.271652	219.102997	1485.72705
23999	Twf2	twinfilin, actin-binding p	50	296.635193	1478.61169
19221	Ptgfrn	prostaglandin F2 recepto	85.0334167	535.422607	1470.36243
225348	Wdr36	WD repeat domain 36	220.20195	520.529846	1469.38635
214459	Fnbp1l	formin binding protein 1-	187.545288	487.3349	1469.25977
76594	Dnajc18	DnaJ (Hsp40) homolog,	150.490906	413.967651	1461.11414
320078	Olfml2b	olfactomedin-like 2B	50	229.045639	1444.77344
94280	Sfxn3	sideroflexin 3	83.553772	283.242065	1444.70435
105837	Mtbp	Mdm2, transformed 3T3	50	102.838669	1429.47949
18793	Plaur	plasminogen activator, u	50	174.914291	1423.4585
67877	Naa20	N(alpha)-acetyltransferas	153.294922	320.519897	1417.60864
16475	Jub	ajuba	67.6690216	441.280182	1415.66455
17974	Nck2	non-catalytic region of ty	117.455612	369.593628	1410.94019
20773	Sptlc2	serine palmitoyltransfera	107.647842	624.715332	1398.97058
20733	Spint2	serine protease inhibitor,	232.49057	631.694092	1388.86108
67824	Nmral1	NmrA-like family domai	174.21109	515.076111	1376.97375
16569	Kif3b	kinesin family member 3	251.996811	579.014282	1376.96387
51793	Ddah2	dimethylarginine dimethy	251.922348	567.597534	1371.97327
20556	Slfn2	schlafen 2	104.679062	407.287354	1363.57935
70472	Atad2	ATPase family, AAA do	50	140.447525	1353.47998
18432	Mybbp1a	MYB binding protein (P	163.341919	328.015045	1352.45288
13555	E2f1	E2F transcription factor	50	181.240906	1348.18701
102545	Cmtm7	CKLF-like MARVEL tra	95.5683289	261.330933	1343.60217
72662	Dis3	DIS3 mitotic control hon	205.492676	458.055908	1333.59631

50766	Crim1	cysteine rich transmembr	92.5947876	330.362061	1332.68921
217333	Trim47	tripartite motif-containin	82.6523666	258.727844	1323.65625
320394	Cenpt	centromere protein T	75.7832794	350.490662	1318.28589
72425	2410042D21Rik	RIKEN cDNA 2410042D	114.521683	329.790527	1311.18701
107566	Arl2bp	ADP-ribosylation factor-	89.4564972	643.389587	1302.01587
15958	Ifit2	interferon-induced protei	90.5923691	594.212585	1287.89697
114641	Rpl31	ribosomal protein L31	124.220139	344.755402	1280.95251
57444	Isg20	interferon-stimulated pro	63.6293182	570.561401	1280.43481
211323	Nrg1	neuregulin 1	50	315.085938	1280.01587
20362	Sept8	septin 8	65.6317444	195.922852	1271.3147
243983	Zdhhc13	zinc finger, DHHC doma	151.636261	421.397766	1266.44238
216766	Gemin5	gem (nuclear organelle) a	181.363739	450.034485	1264.7926
217125	Samd14	sterile alpha motif domai	50	364.134216	1255.05457
54135	Lsr	lipolysis stimulated lipop	204.32196	476.087891	1244.89917
12390	Cav2	caveolin 2	50	105.902939	1241.79663
110749	Chaf1b	chromatin assembly facto	50	109.298264	1235.03125
76477	Pcolce2	procollagen C-endopeptid	52.5811195	128.631805	1227.94727
67619	Nob1	NIN1/RPN12 binding pro	143.865784	461.073486	1226.34827
71242	Spata24	spermatogenesis associat	80.5802307	608.371094	1224.23828
19088	Prkar2b	protein kinase, cAMP de	55.0667267	331.387756	1223.16858
223626	4930572J05Rik	RIKEN cDNA 4930572J	50	162.689728	1212.49048
27388	Ptdss2	phosphatidylserine synth	221.53743	507.854004	1211.4668
16449	Jag1	jagged 1	55.3455429	128.498825	1206.25928
67458	Ergic1	endoplasmic reticulum-g	242.467865	485.968323	1202.14453
68815	Btbd10	BTB (POZ) domain cont	87.3146591	196.838165	1198.27625
67588	Rnf41	ring finger protein 41	123.149223	259.740784	1197.82275
76630	Stambpl1	STAM binding protein li	50	210.351562	1196.15479
19250	Ptpn14	protein tyrosine phospha	56.9344978	353.873291	1194.73657
67920	Mak16	MAK16 homolog (S. cer	125.80909	328.268311	1192.3999
224619	Traf7	TNF receptor-associated	172.143707	396.231323	1192.37036
66569	Gdpd1	glycerophosphodiester ph	58.075119	518.536499	1191.18396
63913	Fam129a	family with sequence sim	50	170.373047	1181.85999
53886	Cdkl2	cyclin-dependent kinase-	207.599655	525.560608	1180.02783
108116	Slco3a1	solute carrier organic ani	50	180.536377	1177.72876
17035	Lxn	latexin	50	390.446564	1169.08362
12048	Bcl2l1	BCL2-like 1	137.445023	359.901367	1161.48511
75739	Mpp7	membrane protein, palmi	243.399353	494.463684	1160.93787
26944	Tinag	tubulointerstitial nephriti	50	287.309387	1156.95129
230145	Galnt12	UDP-N-acetyl-alpha-D-g	50	203.430756	1151.69604
67416	Armcx2	armadillo repeat containi	104.579254	278.778625	1150.76282
58801	Pmaip1	phorbol-12-myristate-13-	50	119.890259	1146.61511
67843	Slc35a4	solute carrier family 35,	107.40863	235.141663	1146.59705
70233	Cd2bp2	CD2 antigen (cytoplasmi	207.081619	430.879181	1144.37695
69940	Exoc1	exocyst complex compor	238.014679	522.240234	1143.47656
21923	Tnc	tenascin C	50	119.637024	1139.91882
66934	Dsn1	DSN1, MIND kinetochor	50	167.363953	1137.63306
226977	Actr1b	ARP1 actin-related prote	182.53447	407.575958	1136.90015
68549	Sgol2	shugoshin-like 2 (S. pom	50	101.108467	1133.34399
19981	Rpl37a	ribosomal protein L37a	168.831146	459.590881	1132.84949
105844	Card10	caspase recruitment dom	165.20018	342.135376	1130.22192
106869	Tnfaip8	tumor necrosis factor, alp	50	157.366135	1129.37891
74838	Naa15	N(alpha)-acetyltransferas	234.667267	560.238159	1126.61157
108927	Lhfp	lipoma HMGIC fusion pa	86.452858	355.989685	1125.08984
230972	Arhgef16	Rho guanine nucleotide e	50	234.314056	1103.26733
19716	Bex1	brain expressed gene 1	50	364.523254	1086.96606
13732	Emp3	epithelial membrane prot	50	164.939117	1085.54785
11717	Ampd3	adenosine monophosphat	56.9043961	331.423096	1083.95068
320982	Arl4c	ADP-ribosylation factor-	50	111.083641	1082.51941
22375	Wars	tryptophanyl-tRNA synth	109.650269	223.78714	1081.47266
97487	Cmtm4	CKLF-like MARVEL tra	173.837219	348.238434	1075.60779
56213	Htra1	HtrA serine peptidase 1	50	293.337494	1074.27661

72018	Fundc1	FUN14 domain containi	125.565125	281.589661	1066.07349
105722	Ano6	anoctamin 6	71.1257401	267.183594	1065.69055
69719	Cad	carbamoyl-phosphate syn	74.40345	163.053299	1057.67969
19069	Nup88	nucleoporin 88	147.3526	372.524902	1057.5564
74044	Ttf2	transcription termination	156.602722	478.168945	1048.00574
67861	Akr1b10	aldo-keto reductase fami	86.9360352	285.920135	1038.18396
19075	Prim1	DNA primase, p49 subun	50	100.877869	1024.69434
11443	Chrnb1	cholinergic receptor, nic	50	143.015228	1018.37103
13017	Ctbp2	C-terminal binding prote	66.7723694	179.124496	1014.77881
20088	Rps24	ribosomal protein S24	151.322601	410.007874	1012.15613
12589	Ift81	intraflagellar transport 8	89.6260071	228.351013	1009.88843
72391	Cdkn3	cyclin-dependent kinase	50	156.09996	1008.49988
19889	Rp2h	retinitis pigmentosa 2 ho	78.0692825	174.154602	1008.46533
13025	Ctla2b	cytotoxic T lymphocyte-	139.377747	323.451172	1007.72095
18475	Pafah1b2	platelet-activating factor	222.787354	471.601837	1006.85498
20621	Snn	stannin	68.3613129	203.969757	1001.18732
28040	D6Wsu163e	DNA segment, Chr 6, W	146.316528	313.180328	994.768677
66912	Bzw2	basic leucine zipper and	122.491776	368.798553	994.147583
22403	Wisp2	WNT1 inducible signalin	50	303.698883	993.891235
67313	5730559C18Rik	RIKEN cDNA 5730559C	50	125.467072	993.449158
329540	8430427H17Rik	RIKEN cDNA 8430427H	50	163.539963	981.671875
56613	Rps6ka4	ribosomal protein S6 kin	104.60936	270.842072	977.539001
22051	Trip6	thyroid hormone recepto	188.202728	462.632507	971.480225
98170	Tmem132a	transmembrane protein 1	50.1303635	128.300751	971.376709
26356	Ing1	inhibitor of growth famil	156.019745	416.99939	969.818909
94249	Slc24a3	solute carrier family 24 (	71.8481369	241.325394	966.400879
66681	Pgm1	phosphoglucomutase 1	62.3239403	302.091766	966.087036
72440	5930416I19Rik	RIKEN cDNA 5930416I	89.5214539	284.751587	959.541931
432486	Gnptab	N-acetylglucosamine-1-p	121.216499	329.501923	958.408081
56449	Csda	cold shock domain prote	70.2940369	193.134445	956.477173
15547	Trmt2a	TRM2 tRNA methyltrans	134.541199	322.506165	955.683533
227753	Gsn	gelsolin	118.21286	305.993561	950.687988
52690	Setd3	SET domain containing 3	50	266.015045	949.503174
18983	Cnot7	CCR4-NOT transcription	183.271118	424.341766	946.926514
23790	Coro1c	coronin, actin binding pr	142.416229	321.535645	946.752319
226154	Lzts2	leucine zipper, putative t	177.110168	390.621979	945.613525
21427	Vps72	vacuolar protein sorting 7	218.369034	469.861755	944.706421
14063	F2rl1	coagulation factor II (thr	50	111.304337	944.246338
97031	Tprn	taperin	50	302.46524	943.027039
170643	Kirrel	kin of IRRE like (Drosop	52.2024956	173.106293	939.334595
57915l100862515	Tbc1d1lLOC10086	TBC1 domain family, me	167.033081	345.329773	937.398804
16776	Lama5	laminin, alpha 5	50	363.660278	930.260498
51812	Mcrs1	microspherule protein 1	120.912338	273.036285	928.370728
102693	Phldb1	pleckstrin homology-like	50	173.216644	927.039673
68501	Nsmce2	non-SMC element 2 hom	91.4240723	190.235687	926.359375
72843	Prdm4	PR domain containing 4	90.8363342	204.61911	921.894592
57278	Bcam	basal cell adhesion mole	62.7025604	257.140503	921.115662
81601	Kat5	K(lysine) acetyltransfera	79.0958405	176.887817	920.499451
69941	2810408I11Rik	RIKEN cDNA 2810408I	103.090103	266.719574	914.309265
494448	Cbx6	chromobox homolog 6	114.242859	312.771484	910.343994
71709	Syde1	synapse defective 1, Rho	79.8879395	188.119263	901.212402
329165	Abi2	abl-interactor 2	90.7666321	256.370911	900.729248
68401	G6pc3	glucose 6 phosphatase, c	134.301971	305.143311	900.165649
19296	Pvt1	plasmacytoma variant tra	50	185.827423	898.083618
225870	Rin1	Ras and Rab interactor 1	114.730797	249.327026	895.852051
118445	Klf16	Kruppel-like factor 16	122.14801	329.23877	893.549805
13078	Cyp1b1	cytochrome P450, family	50	288.134155	890.498291
66617	Mettl11a	methyltransferase like 11	121.525421	352.198242	883.371338
19183	Psmc3ip	proteasome (prosome, m	50	198.159515	881.389587
15499	Hsf1	heat shock factor 1	204.282349	430.048737	881.087219
19038	Ppic	peptidylprolyl isomerase	50	214.72728	879.353577

69178	Snx5	sorting nexin 5	196.068268	403.142212	878.336426
76448	Ppp1r18	protein phosphatase 1, re	92.8039093	368.720734	874.139465
16600	Klf4	Kruppel-like factor 4 (gu	50	166.581604	868.611572
54219	Cd320	CD320 antigen	79.3746643	224.96843	866.421021
68099	Fam92a	family with sequence sin	54.5486908	169.954285	861.594727
21825	Thbs1	thrombospondin 1	50	328.235748	855.04303
15275	Hk1	hexokinase 1	66.8420715	227.380524	846.795471
232023	Vopp1	vesicular, overexpressed	57.0042	176.709564	844.521179
14660	Gls	glutaminase	50	336.513245	842.097351
214230	Pak6	p21 protein (Cdc42/Rac)	50	199.415787	837.760742
104015	Synj1	synaptojanin 1	126.845154	277.785522	836.569397
72309	Tmem158	transmembrane protein 1	173.423752	372.457001	833.231934
94192	C1galt1	core 1 synthase, glycopro	134.162567	302.332275	820.890991
232286	Tmf1	TATA element modulato	55.6243629	150.86409	820.176147
109019	Obfc2a	oligonucleotide/oligosacc	68.0824966	185.661896	817.79834
319520	Dusp4	dual specificity phosphat	50	338.827759	816.769653
67285	Cwc27	CWC27 spliceosome-ass	145.285217	315.692871	810.300049
19094	Mapk11	mitogen-activated protei	55.6243629	126.58046	809.537598
108067	Eif2b3	eukaryotic translation ini	180.532043	364.786377	809.498169
70799	Cep192	centrosomal protein 192	82.9311829	263.554871	805.950317
72515	Wdr43	WD repeat domain 43	91.9072571	218.706879	803.771362
52055	Rab11fip5	RAB11 family interactin	50	243.120667	801.416504
12530	Cdc25a	cell division cycle 25 ho	84.2761688	175.144897	800.614624
68554	1110001A16Rik	RIKEN cDNA 1110001A	154.744476	342.962952	793.737549
104732	4930427A07Rik	RIKEN cDNA 4930427A	50	105.010254	791.011353
108909	Aida	axin interactor, dorsaliza	87.6235809	322.395813	790.853638
72572	Spats2	spermatogenesis associat	57.487381	137.847275	790.704041
80294	Pofut2	protein O-fucosyltransfer	89.2822418	235.261902	786.564697
100217453	Snord16a	small nucleolar RNA, C/	50	109.915085	784.651917
67966	Zcchc10	zinc finger, CCHC doma	70.5380096	144.040909	782.9823
545085	Wdr70	WD repeat domain 70	50	149.07872	780.570007
66111	Tmed3	transmembrane emp24 do	169.897324	382.81842	779.533081
69241	Polr2d	polymerase (RNA) II (D	67.7038727	182.685333	776.246582
11783	Apaf1	apoptotic peptidase activ	121.629974	317.776733	773.264038
76464	Casc5	cancer susceptibility canc	50	116.098816	771.781799
50918	Myadm	myeloid-associated differ	56.7950859	134.517029	770.863159
99526	Usp53	ubiquitin specific peptida	99.3244629	274.214752	768.758179
73739	Cby1	chibby homolog 1 (Dros	55.1063309	146.378021	762.492371
73668	Ttc21b	tetratricopeptide repeat d	90.9060364	221.670715	754.497864
12449	Ccnf	cyclin F	50	186.168365	750.051208
213109	Phf3	PHD finger protein 3	160.886414	331.322662	748.319214
225651	Mppe1	metallophosphoesterase	167.367371	342.300873	739.238464
240055	Neurl1b	neuralized homolog 1b (I	50	214.08783	738.738892
102626	Mapkapk3	mitogen-activated protei	50	118.887222	737.430908
240263	Fem1c	fem-1 homolog c (C.eleg	54.3490829	111.723091	733.565918
107029	Me2	malic enzyme 2, NAD(+	50	142.077271	732.489563
23917	Impdh1	inosine 5'-phosphate deh	76.7543945	206.086182	730.072266
27373	Csnk1e	casein kinase 1, epsilon	70.7772217	244.266586	729.955627
15463	Agfg1	ArfGAP with FG repeats	79.2352524	283.936707	729.852112
69860	Eif1ad	eukaryotic translation ini	51.2804947	147.777191	729.176758
18141	Nup50	nucleoporin 50	105.441055	230.545242	724.927246
75788	Smurf1	SMAD specific E3 ubiqu	93.1825333	213.802063	724.723511
16001	Igf1r	insulin-like growth facto	97.9145355	256.215302	720.439453
14766	Gpr56	G protein-coupled recept	50	254.760971	718.833984
64602	Ireb2	iron responsive element l	104.95787	229.928421	718.061646
259300	Ehd2	EH-domain containing 2	50	137.879807	716.050293
72061	2010111I01Rik	RIKEN cDNA 2010111I	50	136.688614	710.109863
226551	AI848100	expressed sequence AI84	140.622925	340.60321	707.365601
69698	2310046K01Rik	RIKEN cDNA 2310046K	50	129.501846	703.720825
26921	Map4k4	mitogen-activated protei	95.2546692	274.029419	694.983521
75472	1700009P17Rik	RIKEN cDNA 1700009P	50	136.91922	692.594238

319622	Itpripl2	inositol 1,4,5-triphosphat	68.5656738	153.431808	691.547485
56212	Rhog	ras homolog gene family	89.1776733	270.676544	690.099731
100515	Zfp518b	zinc finger protein 518B	104.574493	212.412811	689.623169
67268	Myl12a	myosin, light chain 12A,	50	202.249466	685.78125
76411	1700019E19Rik	RIKEN cDNA 1700019E	94.0791855	244.133606	685.641541
109082	Fbxw17	F-box and WD-40 domai	69.8805618	236.255035	683.403442
18516	Pbx3	pre B cell leukemia homo	50	142.23291	683.37384
233806	Tmem159	transmembrane protein 1	67.3252487	202.635681	678.253418
19164	Psen1	presenilin 1	139.308044	296.381958	676.079346
66822	Fbxo25	F-box protein 25	117.729675	241.533356	675.694824
320183	Msrb3	methionine sulfoxide red	116.937576	263.986328	666.499146
237500	Tmtc3	transmembrane and tetra	90.8014832	211.487579	658.779053
17685	Msh2	mutS homolog 2 (E. coli	88.211319	252.180527	649.810059
102774	Bbs4	Bardet-Biedl syndrome 4	93.2522354	296.161255	646.972107
103268	Cep57l1	centrosomal protein 57-li	80.1018066	170.902161	646.676331
83924|664862	Gpr137b|Gpr137b-	G protein-coupled recept	50	156.892212	639.187927
19893	Rpgr	retinitis pigmentosa GTP	80.8242035	170.538574	635.176758
14537	Gcnt1	glucosaminyl (N-acetyl)	86.1740417	261.285645	635.130676
270669	Mbtps2	membrane-bound transcr	93.9445343	269.696136	635.066589
22379	Fmnl3	formin-like 3	92.7041016	301.153809	630.910767
23999	Twf2	twinfilin, actin-binding p	50	197.201752	630.427612
69399	1700025G04Rik	RIKEN cDNA 1700025G	50	133.757324	629.78186
231123	Haus3	HAUS augmin-like comp	71.2603989	171.002594	623.843018
21824	Thbd	thrombomodulin	50	266.729492	620.441528
68283	9530077C05Rik	RIKEN cDNA 9530077C	50	136.214691	620.121033
226122	Ubtd1	ubiquitin domain contain	93.5263062	188.625732	614.778809
66208	Nenf	neuron derived neurotrop	64.5956726	299.137817	612.865967
68732	Lrrc16a	leucine rich repeat contai	50	121.730804	611.459351
665563	Mthfd2l	methylenetetrahydrofolat	50	105.70488	611.441284
16763	Lad1	ladinin	50	128.531357	607.977295
75458	Cklf	chemokine-like factor	72.7796478	149.572449	602.884766
218699	Pxk	PX domain containing se	64.0919037	255.234894	602.768127
70240	Ufsp1	UFM1-specific peptidase	50	117.874283	600.786377
22393	Wfs1	Wolfram syndrome 1 ho	87.628334	221.946594	598.037109
20617	Snca	synuclein, alpha	50	123.087524	596.467773
239027	Arhgap22	Rho GTPase activating p	50	230.454712	592.607788
237436	Gas2l3	growth arrest-specific 2 l	50	273.653107	591.799316
27886	Dgcr14	DiGeorge syndrome criti	86.7966309	190.995392	585.469421
16579	Kifap3	kinesin-associated protei	121.634727	248.103302	584.09729
171212	Galnt10	UDP-N-acetyl-alpha-D-g	90.6620712	236.595993	583.561584
14911	Thumpd3	THUMP domain contain	133.923355	288.124268	581.394104
432572	Specc1	sperm antigen with calpo	50	129.965881	578.464111
72512	Tmem173	transmembrane protein 1	57.2133141	200.376373	576.842224
233280	Nipa1	non imprinted in Prader-W	133.335617	269.287292	562.268066
69101	Ydjc	YdjC homolog (bacterial	50	226.608093	559.855713
24100	Tpra1	transmembrane protein, a	95.1849518	243.704956	559.170532
211329	Ncoa7	nuclear receptor coactiva	50	163.043396	555.542114
11833	Aqp8	aquaporin 8	50	111.336876	554.414917
55991	Panx1	pannexin 1	50	169.360107	553.700073
193385	Fam65b	family with sequence sim	50	100.491653	550.433228
18011	Neurl1a	neuralized homolog 1A (	50	115.933296	544.509277
67695	Ost4	oligosaccharyltransferase	109.78968	264.164612	544.229858
69568	Vkorc1l1	vitamin K epoxide reduc	74.0945282	170.869629	543.911133
380711	Rap1gap2	RAP1 GTPase activating	50	186.720123	533.749146
68294	Mfsd10	major facilitator superfam	50	199.925079	532.929138
73379	Dcbld2	discoidin, CUB and LCC	50	109.684486	531.348267
237253	Lrp11	low density lipoprotein r	50	241.854492	527.971375
106707	Rpusd1	RNA pseudouridylate sy	50	151.568634	526.075073
67296	Socs4	suppressor of cytokine si	118.905159	247.694458	522.093384
68481	Mpzl1	myelin protein zero-like	55.1015778	203.573639	520.036011
14013	Mecom	MDS1 and EVI1 comple	87.9372559	213.315399	518.849609

104884	Tdp1	tyrosyl-DNA phosphodie	88.4251862	249.612823	518.180786
19877	Rock1	Rho-associated coiled-co	79.0958405	162.647278	512.135193
68259	Ift80	intraflagellar transport 80	71.0861359	164.21196	512.036621
224008	2310008H04Rik	RIKEN cDNA 2310008H	72.296463	192.173859	512.01355
18011	Neurl1a	neuralized homolog 1A (	50	107.909012	511.136078
66854	Trim35	tripartite motif-containin	80.2412186	241.348022	504.061768
67980	Gnpda2	glucosamine-6-phosphate	74.8866272	197.146576	503.363403
434215	Lrrc32	leucine rich repeat contai	50	174.066879	501.049683
56016	Hebp2	heme binding protein 2	50	110.664886	500.224762
22321	Vars	valyl-tRNA synthetase	73.262825	215.191315	498.561768
50492	Thop1	thimet oligopeptidase 1	65.9105606	164.818878	495.59729
69428	1700016C15Rik	RIKEN cDNA 1700016C	65.5271912	157.75235	495.480621
13025	Ctla2b	cytotoxic T lymphocyte-a	53.5126266	107.545425	493.654968
217325	Llgl2	lethal giant larvae homol	61.3179703	134.328873	487.987305
229906	Gtf2b	general transcription fact	50	150.510406	482.122498
67115	Rpl14	ribosomal protein L14	50	110.389015	481.895691
195208	Dcdc2a	doublecortin domain con	72.84935	153.762848	476.890289
67973	Mphosph10	M-phase phosphoprotein	91.319519	201.996231	474.448395
106582	Nrm	nurim (nuclear envelope	76.7940063	180.646729	473.809143
71820	Wdr34	WD repeat domain 34	66.7026596	135.409714	473.541321
217370	BC017643	cDNA sequence BC0176	50	135.013596	470.954803
319996	Casc4	cancer susceptibility can	67.1858368	203.109619	470.803619
330817	Dhps	deoxyhypusine synthase	103.408531	232.044846	469.472595
11352	Abl2	v-abl Abelson murine leu	87.3495178	216.876236	462.927429
380752	Tssc1	tumor suppressing subtra	90.3182983	195.020264	462.270142
74895	4930455F23Rik	RIKEN cDNA 4930455F	94.2185974	199.76947	458.927704
21853	Timeless	timeless homolog (Droso	50	135.211655	457.021515
382985	Rrm2b	ribonucleotide reductase	81.3770905	208.423309	454.213165
14381	G6pdx	glucose-6-phosphate deh	50	193.773895	454.020905
235283	Gramd1b	GRAM domain containin	50	195.757324	453.0448
74360	Cep57	centrosomal protein 57	50	114.12529	452.352966
68146	Arl13b	ADP-ribosylation factor-	50	162.98822	445.82428
66089	Rmnd5b	required for meiotic nucl	93.2173843	190.664337	445.574493
80287	Apobec3	apolipoprotein B mRNA	71.3301086	146.608612	445.365784
17250	Abcc1	ATP-binding cassette, su	50	126.570557	444.830078
60364	Donson	downstream neighbor of	75.0957413	201.379425	443.185181
20514	Slc1a5	solute carrier family 1 (n	65.1881638	163.57251	441.2034
18286	Odf2	outer dense fiber of sperm	62.1496773	145.683395	439.075378
382252	A830080D01Rik	RIKEN cDNA A830080D	54.6881027	112.229568	433.959839
67629	Spc24	SPC24, NDC80 kinetoch	50	142.034836	429.634766
224022	Slc7a4	solute carrier family 7 (c	50	132.796722	428.658661
57265	Fzd2	frizzled homolog 2 (Dros	56.2073479	133.448914	423.199707
225849	Ppp2r5b	protein phosphatase 2, re	76.6450882	190.180511	422.723175
22240	Dpysl3	dihydropyrimidinase-like	50	188.680908	417.729279
58193	Extl2	exostoses (multiple)-like	80.8242035	180.348236	414.143646
191578	Helq	helicase, POLQ-like	50	143.544342	413.323669
11785	Apbb1	amyloid beta (A4) precur	50	111.667923	412.950623
15064	Mr1	major histocompatibility	50	150.344894	408.364258
67326	1700037H04Rik	RIKEN cDNA 1700037H	50	158.161209	406.039062
226352	Epb4.1l5	erythrocyte protein band	50	174.638428	396.0578
16196	Il7	interleukin 7	50	137.415787	395.150726
16210	Impact	imprinted and ancient	52.7949867	120.584885	395.075134
17161	Maoa	monoamine oxidase A	50	106.51976	392.947113
18861	Pms2	postmeiotic segregation i	50	156.363098	389.128143
73738|100048005	Haus7|LOC100048	HAUS augmin-like comp	58.5281982	128.917572	386.710876
98415	Nucks1	nuclear casein kinase and	51.9632835	136.74379	380.321838
252903	Ap1s3	adaptor-related protein co	50	160.926971	375.65332
56321	Aatf	apoptosis antagonizing tr	69.9803696	161.676788	375.525146
68964	1500010J02Rik	RIKEN cDNA 1500010J	72.644989	156.077332	374.752808
27392	Pign	phosphatidylinositol glyc	59.524662	180.182709	370.125366
77579	Myh10	myosin, heavy polypepti	50	182.519806	370.038269

	75568	Capsl	calcyphosine-like	50	142.927521	369.206787
	73914	Irak3	interleukin-1 receptor-ass	50	133.426285	368.579041
	78255	Ralgps2	Ral GEF with PH domai	50	117.014137	367.451752
	60363	Cldn15	claudin 15	61.0740051	179.98465	367.381104
	75570	Nhej1	nonhomologous end-join	64.8744965	170.869629	365.852844
	78394	Ddx52	DEAD (Asp-Glu-Ala-As	66.9418793	142.982697	364.5448
	207175	Cetn4	centrin 4	50	108.074539	364.184937
	239393	Lrp12	low density lipoprotein-r	62.7326622	143.820221	362.102905
	212123	Dcaf15	DDB1 and CUL4 associa	63.3203964	152.451401	360.900024
	216892	Spns2	spinster homolog 2 (Dros	50	106.961151	360.783356
	216618	Ccdc104	coiled-coil domain conta	64.3168564	135.322006	356.801697
	76453	Prss23	protease, serine, 23	50	101.108467	354.458405
	327900	Ubtd2	ubiquitin domain contain	67.3252487	149.299408	352.930176
	66871	Cpne8	copine VIII	62.9370232	153.597321	352.412537
	69159	Rhebl1	Ras homolog enriched in	50	108.184883	352.336945
	170835	Inpp5j	inositol polyphosphate 5-	50	101.890808	348.628082
	171531	Mlph	melanophilin	59.071579	127.550964	344.861725
	16196	Il7	interleukin 7	50	121.643097	343.936554
	18844	Plxna1	plexin A1	50	115.040604	339.448792
	227399	Ppip5k2	diphosphoinositol pentak	50	152.097717	337.100525
	16440	Itpr3	inositol 1,4,5-triphosphat	50	108.967224	336.385742
	210148	Slc30a6	solute carrier family 30 (	60.3167572	156.60643	330.159363
	20723	Serpinb9	serine (or cysteine) pepti	50	151.536087	326.40448
	66196	Myo19	myosin XIX	59.5199051	160.011658	321.689941
	16563	Kif2a	kinesin family member 2	60.4213142	138.970566	321.643921
	217578|10004855	Baz1a|LOC100048	bromodomain adjacent to	58.3586922	155.879272	320.585693
	72462	Rrp1b	ribosomal RNA processi	56.695282	133.712067	320.184723
	66595	Aste1	asteroid homolog 1 (Dros	50	100.248322	320.143646
	77300	Raph1	Ras association (RalGDS	57.487381	142.343246	316.49884
	59053	Fam203a	family with sequence sim	50	129.612198	309.644775
	11641	Akap2	A kinase (PRKA) anchor	50	131.751251	308.570068
	74166	Tmem38a	transmembrane protein 3	51.3058395	147.85498	305.0354
	109929	Zbtb25	zinc finger and BTB dom	50	128.797318	297.152649
	244329	Mcph1	microcephaly, primary au	52.4021072	112.758667	294.483948
	217578	Baz1a	bromodomain adjacent to	50	101.835632	293.908813
	71566	9030425E11Rik	RIKEN cDNA 9030425E	50	131.673447	291.519501
	103841	Cuedc1	CUE domain containing	54.7578049	112.13195	291.507996
	56312	Nupr1	nuclear protein 1	50	130.108765	288.770325
	380753	Atxn7l1	ataxin 7-like 1	50	107.909012	287.572388
	67157	2610301B20Rik	RIKEN cDNA 2610301B	50	100.556725	287.182922
	216835	Usp43	ubiquitin specific peptida	50	122.944633	286.479614
	57258	Xpo4	exportin 4	50	128.488922	285.897888
	21808	Tgfb2	transforming growth fact	50	127.849457	282.555481
	75316	Taf1d	TATA box binding prote	50	107.214386	279.201538
	53618	Fut8	fucosyltransferase 8	50.3093796	106.123642	278.282959
	18452	P4ha2	procollagen-proline, 2-ox	59.4597092	133.283401	277.939514
	212391	Lcor	ligand dependent nuclear	50	109.165283	277.103088
	54384	Mtmr7	myotubularin related pro	50	133.900208	270.929321
	73047	Camk2n2	calcium/calmodulin-depe	50	132.841995	266.093201
	224523	4732491K20Rik	RIKEN cDNA 4732491K	50	127.297729	264.267517
	72355	2210021J22Rik	RIKEN cDNA 2210021J	50	100.887772	263.348938
	74133	1200011M11Rik	RIKEN cDNA 1200011M	50	103.91951	258.774048
	72149	Strada	STE20-related kinase ada	50	106.01329	243.171204
	268396	Sh3pxd2b	SH3 and PX domains 2B	50	100.998116	241.061249
	19326	Rab11b	RAB11B, member RAS	50	110.025436	227.533829
	12830	Col4a5	collagen, type IV, alpha 5	50	103.169708	227.38266
	13488	Drd1a	dopamine receptor D1A	50	100.722244	203.246292
Pattern I -Down	216454	BC089597	cDNA sequence BC0895	18599.9023	1750.01294	50
	21936	Tnfrsf18	tumor necrosis factor rec	346.733459	104.39344	50
	75104	Mmd2	monocyte to macrophage	889.570007	110.3013	50
	54150	Rdh7	retinol dehydrogenase 7	12174.1455	5574.52002	50

53412	Ppp1r3c	protein phosphatase 1, re	2828.66016	686.556885	50
94179	Krt23	keratin 23	379.394836	120.948471	50
71773	Ugt2b1	UDP glucuronosyltransfe	8916.39648	2163.38086	50
13124	Cyp8b1	cytochrome P450, family	11715.2129	5670.45801	50
20211	Saa4	serum amyloid A 4	5370.1084	2197.74707	50
71911	Bdh1	3-hydroxybutyrate dehyd	3162.01367	1541.63782	50
16325	Inhbc	inhibin beta-C	1349.71924	412.094574	50
23945	Mgll	monoglyceride lipase	11059.4941	5360.46484	50
207742	Rnf43	ring finger protein 43	300.085144	137.603943	50
12355	Nr1i3	nuclear receptor subfami	2003.02368	820.783875	50
15450	Lipc	lipase, hepatic	994.119141	455.95224	50
11835	Ar	androgen receptor	436.921844	113.508469	50
75178	4930528F23Rik	RIKEN cDNA 4930528F	601.977844	127.924438	50
17167	Marco	macrophage receptor wit	693.113586	228.682068	50
20520	Slc22a5	solute carrier family 22 (	315.451874	121.984039	50
171281	Acot3	acyl-CoA thioesterase 3	5349.12256	343.170929	50
384198	Fam47e	family with sequence sim	1168.22095	150.588226	50
64385	Cyp4f14	cytochrome P450, family	3376.84326	939.09668	50
272428	Acsm5	acyl-CoA synthetase med	3076.46826	1213.6001	50
68444	Cyp2d13	cytochrome P450, family	943.217285	200.91539	50
100038948	Mup9	major urinary protein 9	1597.84595	107.710953	50
23957	Nr0b2	nuclear receptor subfami	395.967163	113.144882	50
56636|100862558	Fgf21|LOC100862	fibroblast growth factor 2	1517.55408	368.65564	50
13482	Dpp4	dipeptidylpeptidase 4	403.289307	195.205597	50
320571	Atp8b5	ATPase, class I, type 8B,	302.54541	132.653839	50
20532	Slc3a1	solute carrier family 3, m	1075.50098	244.92868	50
217082	Hlf	hepatic leukemia factor	932.139038	401.314423	50
116852	Akr1c20	aldo-keto reductase fami	415.712585	189.927277	50
14451	Gas1	growth arrest specific 1	230.901627	114.115379	50
13095	Cyp2c29	cytochrome P450, family	18297.0352	6966.94043	50
64697	Keg1	kidney expressed gene 1	499.201416	247.992966	50
18559	Pctp	phosphatidylcholine trans	1848.89062	794.250854	50
226781	Slc30a10	solute carrier family 30,	469.464417	107.522797	50
83702	Akr1c6	aldo-keto reductase fami	654.519348	135.354553	50
13897	Ces1e	carboxylesterase 1E	1811.78247	595.07843	50
66438	Hamp2	hepcidin antimicrobial pe	3872.55176	189.794296	50
54357	Epb4.1l4b	erythrocyte protein band	1146.2688	484.082489	50
109254	9530008L14Rik	RIKEN cDNA 9530008L	2816.74072	1112.53406	50
171282	Acot4	acyl-CoA thioesterase 4	872.484375	173.437332	50
12350	Car3	carbonic anhydrase 3	6733.21875	1547.89648	50
19266	Ptprd	protein tyrosine phospha	695.957214	323.087585	50
234564	Ces1f	carboxylesterase 1F	1952.41016	367.010345	50
13094	Cyp2b9	cytochrome P450, family	2839.35352	258.045959	50
13089	Cyp2b13	cytochrome P450, family	2866.3562	208.98494	50
13109	Cyp2j5	cytochrome P450, family	3842.21582	536.659058	50
75552	Paqr9	progestin and adipoQ rec	343.420868	115.547073	50
337924|53973|100	Cyp3a44|Cyp3a41a	cytochrome P450, family	17973.1445	2851.57861	50
107141|404195|13	Cyp2c50|Cyp2c54|	cytochrome P450, family	16145.7305	2608.37891	50
72948	Tppp	tubulin polymerization pr	384.187073	191.579666	50.6094513
57742	Abhd1	abhydrolase domain cont	2649.22266	878.618958	53.3882217
69638	Enho	energy homeostasis assoc	1208.32007	396.364319	53.539402
75698	Fam35a	family with sequence sim	422.77652	183.445038	54.7718582
215929	AI317395	expressed sequence AI31	883.399536	378.585571	60.7418594
18628	Per3	period homolog 3 (Droso	541.656372	244.828232	64.6249084
67307	Pbld2	phenazine biosynthesis-li	10919.0947	5423.56396	73.9669037
66116	Cml1	camello-like 1	1219.1814	576.088623	79.5359497
15439	Hp	haptoglobin	20623.6348	7430.90039	87.1919403
100216530	Snord118	small nucleolar RNA, C/	2870.16943	378.455383	90.662529
68616	Gdpd3	glycerophosphodiester ph	7153.09277	3260.56104	98.568306
192136	5033411D12Rik	RIKEN cDNA 5033411D	1141.25317	346.874664	103.747894
232370	Clstn3	calsyntenin 3	1265.73462	434.615479	109.113174

	15483	Hsd11b1	hydroxysteroid 11-beta d	49274.5078	16211.248	111.415398
	16841	Lect2	leukocyte cell-derived ch	3501.32031	1611.0127	120.292343
	75735	Pank1	pantothenate kinase 1	1435.99792	624.617737	153.502838
	12401	Serpina6	serine (or cysteine) pepti	13168.4355	4677.94678	168.588074
	14373	G0s2	G0/G1 switch gene 2	7635.54346	3459.38672	174.459473
	100217422	Snord13	small nucleolar RNA, C/	1318.35864	564.611023	195.991257
	69772	Bdh2	3-hydroxybutyrate dehyd	1298.11401	528.291016	262.90686
	13170	Dbp	D site albumin promoter	4214.5752	2055.66284	307.15686
	67475	Ero1lb	ERO1-like beta (S. cerev	2370.48315	1182.3999	338.204834
	19363	Rad51l1	RAD51-like 1 (S. cerevis	6146.80273	924.372314	357.353882
	20322	Sord	sorbitol dehydrogenase	11552.6602	3874.11816	562.780762
	22361	Vnn1	vanin 1	16013.2354	6664.88281	1327.27136
	67442	Retsat	retinol saturase (all trans	33140.9688	13459.6035	1890.65186
Pattern II -Up	12468	Cct7	chaperonin containing To	22120.9316	26603.1016	65430.1641
	23980	Pebp1	phosphatidylethanolamin	18629.1348	16641.0859	41907.8086
	15387l100504131	HnrnpklGm20077	heterogeneous nuclear ri	13175.6221	16883.875	40963.3594
	14873	Gsto1	glutathione S-transferase	12182.5176	11097.4551	36592.375
	276770	Eif5a	eukaryotic translation ini	13489.2832	15265.4873	31305.8555
	67332	Snrpd3	small nuclear ribonucleo	6598.4165	7614.50098	30101.7324
	22608	Ybx1	Y box protein 1	8007.22461	6471.17773	28225.5742
	12443	Ccnd1	cyclin D1	3996.84155	3391.0376	27519.8984
	71994	Cnn3	calponin 3, acidic	7428.07324	10756.7734	26488.2852
	20610	Sumo3	SMT3 suppressor of mif	8176.70068	10975.0273	23373.2207
	66271	Tmem126a	transmembrane protein 1	7341.04883	8264.38086	22297.5195
	382010	BC088983	cDNA sequence BC0889	1285.35327	1179.61157	22196.2617
	12466	Cct6a	chaperonin containing To	4182.33203	3883.92236	21924.4355
	19166	Psma2	proteasome (prosome, ma	5701.87402	7244.95801	20830.1445
	276770	Eif5a	eukaryotic translation ini	3384.99878	3632.77466	20795.0195
	15516	Hsp90ab1	heat shock protein 90 alp	5374.80225	6741.94824	18712.1562
	19181	Psmc2	proteasome (prosome, ma	6621.05176	8063.58301	18198.9336
	15526	Hspa9	heat shock protein 9	7001.83398	6777.4292	17563.4336
	276770	Eif5a	eukaryotic translation ini	2293.07129	2518.85132	16365.1865
	17149	Magoh	mago-nashi homolog, pro	4202.8584	5278.90527	15518.4189
	20867	Stip1	stress-induced phosphopr	2027.77832	2278.90381	15122.7988
	56350	Arl3	ADP-ribosylation factor-	1357.6261	1874.4657	14574.8359
	68544	2310036O22Rik	RIKEN cDNA 2310036O	3608.21533	5110.13037	14312.5029
	14791	Emg1	EMG1 nucleolar protein	6120.88672	6792.13672	14301.0195
	67089	Psmc6	proteasome (prosome, ma	5540.29883	5484.31934	13878.416
	54198	Snx3	sorting nexin 3	2198.53735	2811.09814	13570.4609
	12464	Cct4	chaperonin containing To	4911.35303	6582.43164	13349.7012
	22384	Eif4h	eukaryotic translation ini	5210.65869	6531.26123	13241.4619
	107765	Ankrd1	ankyrin repeat domain 1	50	50	13167.7012
	20973	Syngr2	synaptogyrin 2	4124.64648	6144.07812	12632.1045
	19045	Ppp1ca	protein phosphatase 1, ca	1945.30652	2572.08569	12187.7822
	67068	Dynlrb1	dynein light chain roadbl	4247.64844	5204.32666	12114.0469
	68041	Mid1ip1	Mid1 interacting protein	1535.63135	1723.24658	12092.3477
	15901	Id1	inhibitor of DNA binding	1845.78406	2262.46338	11904.3936
	100504986l66882	LOC100504986lBz	uncharacterized LOC100	4248.42334	5341.37012	11707.8945
	100041585l11702	Amd2lAmd1	S-adenosylmethionine de	4038.729	5242.40527	11446.8105
	12465	Cct5	chaperonin containing To	3155.99829	3756.53979	11416.1924
	72787	Tmem48	transmembrane protein 4	385.527283	486.121094	11256.7891
	211556	Ap1ar	adaptor-related protein co	1875.97412	2748.76709	11050.5918
	26441	Psma4	proteasome (prosome, ma	5135.77832	5323.04541	10869.667
	12785	Cnbp	cellular nucleic acid bind	3690.21826	4833.28857	10833.7051
	66647	Ndnl2	necdin-like 2	3408.4165	3470.94092	10794.834
	171283	Havcr1	hepatitis A virus cellular	50	50	10719.5156
	76959	Chmp5	charged multivesicular bo	2882.07617	3190.71338	9963.78125
	26987	Eif4e2	eukaryotic translation ini	2124.25586	2632.46582	9803.52637
	12261	C1qbp	complement component	1061.28918	1387.14771	9708.45996
	56332	Amotl2	angiomotin-like 2	371.245728	391.900879	9651.38281
	12387	Ctnnb1	catenin (cadherin associa	2771.69092	3812.00952	9645.94629

66235	Eif1ax	eukaryotic translation ini	1077.65393	1549.8418	9595.85938
59054	Mrps30	mitochondrial ribosomal	3410.21289	3945.1499	9363.15039
19170	Psmb1	proteasome (prosome, m	4340.03906	3790.41528	9017.45703
20643	Snrpe	small nuclear ribonucleo	1380.94543	2047.98364	8985.25293
225363	Etf1	eukaryotic translation ter	1953.89136	2256.64893	8968.59863
66181	Nop10	NOP10 ribonucleoprotei	2204.15015	2776.12476	8900.60156
110750	Cse1l	chromosome segregation	1342.80591	1783.60962	8887.26465
68095	Ociad1	OCIA domain containing	3117.73975	4281.75391	8872.15332
66256	Ssr2	signal sequence receptor,	2845.47656	3975.69092	8855.36523
13200	Ddost	dolichyl-di-phosphooligo	3602.77856	4128.38867	8842.70898
12301	Cacybp	calcyclin binding protein	1963.64844	2127.99023	8622.75195
209318	Gps1	G protein pathway suppr	2973.06934	3746.01416	8479.18359
12343	Capza2	capping protein (actin fil	1569.09424	2158.05713	8466.10352
66448	Mrpl20	mitochondrial ribosomal	2982.30664	3055.38867	8465.88672
14870	Gstp1	glutathione S-transferase	2667.90039	2870.19092	8398.41406
12462	Cct3	chaperonin containing Tc	1377.58862	1343.88855	8292.02734
19072	Prep	prolyl endopeptidase	912.51239	1107.68726	8229.77734
94066	Mrpl36	mitochondrial ribosomal	2456.34814	3322.05591	8078.10986
23874	Farsb	phenylalanyl-tRNA synth	2509.54883	2875.4126	7891.64795
20810	Srm	spermidine synthase	804.56665	933.604858	7861.46094
68212	Tmbim4	transmembrane BAX inh	2576.2356	3263.3916	7734.40332
407819	BC031181	cDNA sequence BC0311	3060.83228	3451.46021	7681.81543
74287	Kcmf1	potassium channel modu	2406.35889	3425.92725	7652.60156
26443	Psma6	proteasome (prosome, m	3182.66016	3081.6062	7647.95947
218335	Clptm1l	CLPTM1-like	2835.33105	3084.89111	7623.14551
26446	Psmb3	proteasome (prosome, m	2785.88354	2930.64014	7610.43848
56207	Uchl5	ubiquitin carboxyl-termi	1086.66321	1315.89697	7556.14941
66357	Ostc	oligosaccharyltransferase	2142.09229	2677.99707	7554.79102
23837	Cfdp1	craniofacial development	2924.4502	3728.7251	7540.76855
15460	Hr	hairless	3207.15186	2744.0249	7533.09277
26445	Psmb2	proteasome (prosome, m	2382.60229	2454.70093	7499.27783
20382	Srsf2	serine/arginine-rich splic	1056.01062	1261.67224	7476.21973
68275	Rpa1	replication protein A1	1261.96265	1835.33032	7256.2168
98711	Rdh10	retinol dehydrogenase 10	548.425659	441.413177	7185.57568
52502	Carhsp1	calcium regulated heat st	2120.77075	2919.21631	7168.78613
67054	Paics	phosphoribosylaminoimi	2456.68115	2252.2959	7147.48926
110052	Dek	DEK oncogene (DNA bi	1283.85315	1913.50195	7135.75
12793	Cnih	cornichon homolog (Dro	2160.93652	2889.28662	7104.38184
20637	Snrnp70	small nuclear ribonucleo	1557.57886	2196.20068	7031.97461
16911	Lmo4	LIM domain only 4	1506.56604	1881.24084	7000.53369
14621	Gjb4	gap junction protein, beta	50	50	6988.9043
24030	Mrps12	mitochondrial ribosomal	2066.14917	2499.57153	6939.71484
67804	Snx2	sorting nexin 2	1886.30151	2580.98145	6873.80469
15191	Hdgf	hepatoma-derived growth	1936.59656	2798.01465	6855.32275
12345	Capzb	capping protein (actin fil	2122.44214	3108.91895	6836.71582
66143	Eef1e1	eukaryotic translation elo	391.135345	499.070007	6652.18945
14852	Gspt1	G1 to S phase transition	1151.6438	1415.28223	6584.82422
22192	Ube2m	ubiquitin-conjugating enz	704.570557	1051.27112	6564.25342
66276	1810009A15Rik	RIKEN cDNA 1810009A	758.759583	955.022217	6526.91992
230126	Shb	src homology 2 domain-c	3114.85034	3176.39233	6428.20898
107071	Wdr74	WD repeat domain 74	1165.54199	1441.71765	6372.16992
68202	Ndufa5	NADH dehydrogenase (u	3130.4895	3080.62598	6340.30371
26891	Cops4	COP9 (constitutive photo	1890.14478	2126.6123	6328.4834
12558	Cdh2	cadherin 2	793.741821	866.336426	6256.91406
52615	Suz12	suppressor of zeste 12 ho	1740.27173	2322.65405	6228.87842
56047	Msln	mesothelin	50	50	6204.8667
23897	Hax1	HCLS1 associated X-1	1253.82312	1425.96191	6142.81055
268656	Sptlc1	serine palmitoyltransfera	1394.61865	1116.16431	6080.32031
52502	Carhsp1	calcium regulated heat st	1753.00867	2399.65283	6025.22754
218121	Mboat1	membrane bound O-acyl	50	50	5967.36328
74493	Tnks2	tankyrase, TRF1-interact	1931.34338	2615.27686	5963.47217

68441	Rraga	Ras-related GTP binding	2105.27734	2910.85962	5879.90332
52858	Cdipt	CDP-diacylglycerol--ino	2423.22412	2577.42773	5688.26562
235036	Ppan	peter pan homolog (Dros	1682.99341	2515.81396	5636.04541
225995	D030056L22Rik	RIKEN cDNA D030056	637.429077	803.960083	5635.1123
26356	Ing1	inhibitor of growth famil	605.947876	901.27002	5630.49414
21915	Dtymk	deoxythymidylate kinase	784.417236	1153.40527	5599.87402
50927	Nasp	nuclear autoantigenic sp	504.944122	540.17749	5531.27393
20901	Strap	serine/threonine kinase r	1529.17896	2124.5144	5416.19238
13204	Dhx15	DEAH (Asp-Glu-Ala-His	1718.51587	2084.04492	5411.64209
75062	Sf3a3	splicing factor 3a, subun	1292.39197	1383.73413	5293.65332
230075	Ndufb6	NADH dehydrogenase (u	2193.62158	2162.42871	5272.61133
26754	Cops5	COP9 (constitutive photo	1820.12476	2331.00098	5222.40625
67180	Yipf5	Yip1 domain family, mem	1757.02783	2477.68018	5202
66868	Mfsd1	major facilitator superfam	2480.6167	2485.00977	5201.33105
22370	Vtn	vitronectin	1819.39771	2497.65039	5196.41943
27979	Eif3b	eukaryotic translation ini	1050.45959	1294.08911	5170.88965
226016	Fam108b	family with sequence sim	1210.18799	1651.8811	5072.93115
66890	Lman2	lectin, mannose-binding 2	1981.67334	2013.45752	5060.84961
18813	Pa2g4	proliferation-associated 2	2154.70581	2448.55664	5035.33154
68051l100043462l	Nutf2lNutf2-ps1lG	nuclear transport factor 2	1241.45996	1536.38916	5029.72266
211652	Wwc1	WW, C2 and coiled-coil	968.250732	1162.26001	4961.27051
53893	Nudt5	nudix (nucleoside diphos	566.810242	596.386963	4956.22607
97820	4833439L19Rik	RIKEN cDNA 4833439L	1501.7627	2146.50317	4948.20703
216443	Mars	methionine-tRNA synthe	1324.65723	1955.45544	4917.68799
70396	Asnsd1	asparagine synthetase do	1760.08374	1778.32007	4909.80664
75616	2810008M24Rik	RIKEN cDNA 2810008M	895.726196	996.926147	4905.63965
19823	Rnf7	ring finger protein 7	1593.05859	2347.98291	4898.93164
76936	Hnrnpm	heterogeneous nuclear rib	1635.4231	2038.84473	4858.19189
105083	Pelo	pelota homolog (Drosoph	1726.88843	2022.0929	4832.93311
29864	Rnf11	ring finger protein 11	2117.70532	1500.91943	4809.07129
14976	H2-Ke2	H2-K region expressed g	1635.21094	2321.09912	4781.37012
20311	Cxcl5	chemokine (C-X-C motif	50	59.2016945	4778.77588
70025	Acot7	acyl-CoA thioesterase 7	369.292419	464.197205	4771.59961
97130	C77080	expressed sequence C770	1871.82983	2217.97607	4715.80371
266781	Snx17	sorting nexin 17	1236.02942	1787.88501	4667.7417
19352	Rabggtb	RAB geranylgeranyl tran	1649.12646	1859.43579	4647.63281
72692	Hnrpll	heterogeneous nuclear rib	866.386841	1288.99316	4632.82227
381038	Parl	presenilin associated, rho	997.922852	1303.46436	4626.88477
19718	Rfc2	replication factor C (acti	955.380737	1042.25366	4617.36865
22234	Ugcg	UDP-glucose ceramide g	1318.46313	1519.5625	4568.82324
56736	Rnf14	ring finger protein 14	1314.74658	1912.50464	4558.83057
66521	Rwdd1	RWD domain containing	400.236572	561.157715	4525.69043
67781	Ilf2	interleukin enhancer bind	468.612152	647.564453	4519.09668
76561	Snx7	sorting nexin 7	417.116211	526.917297	4480.96289
225288	Fhod3	formin homology 2 doma	89.1428223	73.6954803	4479.43066
66997	Psmd12	proteasome (prosome, ma	1685.58362	1828.65857	4421.28906
17846	Commd1	COMM domain containi	839.244751	1068.35815	4413.90723
19720	Trim27	tripartite motif-containin	838.956421	1122.22351	4391.89893
19139	Prps1	phosphoribosyl pyrophos	1144.14587	1337.57605	4374.07617
22122	Tsta3	tissue specific transplanta	1636.94714	2038.23486	4301.2085
69790	Med30	mediator complex subuni	540.081665	655.445801	4290.92676
12540	Cdc42	cell division cycle 42 ho	1526.6853	1869.87073	4287.59229
66366	Ergic3	ERGIC and golgi 3	717.77002	811.279785	4268.83936
67205	Utp11l	UTP11-like, U3 small nu	916.891113	1295.01147	4234.64941
433702	Ncbp1	nuclear cap binding prote	954.972046	960.7547	4214.5
15115	Hars	histidyl-tRNA synthetase	904.572388	1102.03967	4207.73096
64657	Mrps10	mitochondrial ribosomal	1815.8855	1710.22546	4171.5625
70356	St13	suppression of tumorigen	993.441101	1295.23926	4149.62793
226830	Smyd2	SET and MYND domain	1237.62146	1694.39758	4118.03467
71974	Prmt3	protein arginine N-methy	1257.03589	1841.68237	4105.13672
109054	Pfdn4	prefoldin 4	462.435333	615.480042	4084.16357

56445	Dnaja2	DnaJ (Hsp40) homolog,	1011.54846	1030.87231	4059.19897
12977	Csf1	colony stimulating factor	142.42099	204.742203	4047.95752
66917	Chordc1	cysteine and histidine-ric	1684.104	1128.63501	4039.72461
77862	Thyn1	thymocyte nuclear protei	1097.8667	1574.05322	4039.14795
68090	Yif1a	Yip1 interacting factor h	1977.93469	1919.79468	4002.56543
14620	Gjb3	gap junction protein, beta	50	50	3979.72559
66126	Elof1	elongation factor 1 homo	1227.58081	1399.76282	3961.93213
72612	2700029M09Rik	RIKEN cDNA 2700029M	791.207153	820.946594	3928.8894
70160	Vps36	vacuolar protein sorting	1308.17212	1562.60815	3925.64062
11933	Atp1b3	ATPase, Na+/K+ transpo	1319.28223	1903.14209	3919.88916
66958	Tmx2	thioredoxin-related trans	1443.34546	1910.698	3917.0625
12539	Cdc37	cell division cycle 37 ho	844.151001	1200.82666	3898.97681
229877	Rap1gds1	RAP1, GTP-GDP dissoc	1000.1803	1292.52148	3884.86938
67166	Arl8b	ADP-ribosylation factor-	1205.25952	1798.49963	3883.82422
60411	Cenpk	centromere protein K	114.27771	139.752899	3855.39233
12181	Bop1	block of proliferation 1	424.340149	558.625366	3840.55371
54709	Eif3i	eukaryotic translation ini	604.642456	454.452637	3804.72876
66537	Pomp	proteasome maturation p	881.918335	1136.54468	3795.82715
67618	Aasdhppt	aminoadipate-semialdehy	972.868652	729.306885	3789.91797
229877	Rap1gds1	RAP1, GTP-GDP dissoc	804.959595	949.234619	3772.97754
27407	Abcf2	ATP-binding cassette, su	245.924591	286.812805	3760.19287
66928	3110001D03Rik	RIKEN cDNA 3110001I	1198.66284	1244.88232	3747.14697
22680	Zfp207	zinc finger protein 207	707.084656	934.633301	3715.23291
68079	Pdcd2l	programmed cell death 2	1082.67432	1568.45093	3685.54053
13511	Dsg2	desmoglein 2	1288.88135	1692.77637	3683.83154
59050	Nsa2	NSA2 ribosome biogenes	810.848022	1013.46704	3677.84863
83703	Dbr1	debranching enzyme hom	511.176392	500.150818	3639.82471
17220	Mcm7	minichromosome mainte	231.389557	345.462769	3595.54858
195040	Tmem199	transmembrane protein 1	672.062744	932.569214	3587.08398
68565	Mrps18a	mitochondrial ribosomal	1020.40576	1427.77002	3532.04932
12757	Clta	clathrin, light polypeptide	911.57135	1146.03601	3512.05566
20479	Vps4b	vacuolar protein sorting 4	1332.49756	1669.70801	3511.63477
66399	Tsfm	Ts translation elongation	651.53949	948.617798	3507.27539
70358	Steap1	six transmembrane epithe	50	50	3463.28516
66310	Dpy30	dpy-30 homolog (C. eleg	820.56073	1180.83386	3434.83496
14227	Fkbp2	FK506 binding protein 2	1136.47998	1501.11035	3434.62646
69885	2610002D18Rik	RIKEN cDNA 2610002I	50	69.6281662	3406.7959
20224	Sar1a	SAR1 gene homolog A (	517.766602	640.488037	3380.73682
72084	Pigx	phosphatidylinositol glyc	1296.04041	1420.65674	3377.97607
107732	Mrpl10	mitochondrial ribosomal	782.145508	1002.09277	3376.08496
100972	Rab28	RAB28, member RAS or	968.784668	1125.86206	3364.24658
17218	Mcm5	minichromosome mainte	50	50	3345.2998
28106	D17Wsu104e	DNA segment, Chr 17, V	807.700195	826.205078	3316.40283
209354	Eif2b1	eukaryotic translation ini	751.263184	892.515747	3305.13672
223691	Eif3l	eukaryotic translation ini	707.995544	837.494507	3291.25757
13722	Aimp1	aminoacyl tRNA synthet	703.160583	1033.18823	3284.62207
13726	Emd	emerin	289.703888	359.196838	3278.76855
15182	Hdac2	histone deacetylase 2	521.825317	691.840881	3248.5144
68184	Denr	density-regulated protein	1131.56885	1383.76233	3243.24951
66495	Ndufb3	NADH dehydrogenase (u	1116.51111	1078.54688	3241.67017
109778	Blvra	biliverdin reductase A	1023.84668	1492.30518	3241.62915
60409	Trappc4	trafficking protein particl	222.448334	227.558777	3239.53223
68115	9430016H08Rik	RIKEN cDNA 9430016H	1085.08545	1512.93604	3237.91357
67767	Jagn1	jagunal homolog 1 (Dros	964.222168	1280.57983	3231.3457
66998	Psmd5	proteasome (prosome, m	825.473328	1116.61694	3229.44922
66844	Ormdl2	ORM1-like 2 (S. cerevisi	1223.52222	1274.14294	3207.68896
66671	Ccnh	cyclin H	1103.78369	1166.25659	3204.23486
66407	Mrps15	mitochondrial ribosomal	978.39917	1130.6311	3163.04639
68794	Flnc	filamin C, gamma	50	50	3161.68896
72635	Lins	lines homolog (Drosophi	997.33667	1049.99512	3043.66748
74168	Zdhhc16	zinc finger, DHHC doma	268.743347	380.52655	3034.64941

268996	Ss18	synovial sarcoma translo	970.324524	1322.2688	3019.0022
28015	Polr2m	polymerase (RNA) II (D	802.953979	967.582092	3017.88965
12928	Crk	v-crk sarcoma virus CT1	558.168457	766.895874	2989.63184
74137	Nuak2	NUAK family, SNF1-lik	496.332458	539.908691	2964.01831
105148	Iars	isoleucine-tRNA synthet	409.914429	581.587646	2956.49854
58184	Rqcd1	rcd1 (required for cell di	532.933716	774.533936	2953.26953
100505064l56397	LOC100505064lM	mortality factor 4-like pr	231.319855	249.437378	2950.27246
20638	Snrpb	small nuclear ribonucleo	551.513245	772.339722	2948.97266
109075	Exosc4	exosome component 4	811.046021	949.289795	2929.7561
66618	Snrnp27	small nuclear ribonucleo	958.962646	1312.57666	2928.02393
17274	Rab8a	RAB8A, member RAS o	1253.5332	1434.15186	2921.8667
56258	Hnrnph2	heterogeneous nuclear ri	726.491089	1057.01917	2916.00537
68572	Ict1	immature colon carcinom	1049.85278	1405.53625	2912.12695
66799	Ube2w	ubiquitin-conjugating en	682.10022	986.778442	2911.15283
22402	Wisp1	WNT1 inducible signalin	50	67.9531403	2909.91699
66073	Txndc12	thioredoxin domain conta	1032.11621	1142.88831	2894.1958
56390	Sssca1	Sjogren's syndrome/scler	353.886108	429.76297	2891.61255
11772	Ap2a2	adaptor protein complex	898.151611	1138.4021	2870.61816
15528	Hspe1	heat shock protein 1 (cha	498.773682	632.538696	2857.49487
68026	2810417H13Rik	RIKEN cDNA 2810417H	50	50	2851.17822
11732	Ank	progressive ankylosis	676.810608	591.118652	2837.18262
20492	Slbp	stem-loop binding protein	346.250244	507.791748	2828.97754
83435	Plekha3	pleckstrin homology dom	480.243286	651.985474	2817.98096
94067	Mrpl43	mitochondrial ribosomal	961.543335	1097.55371	2790.34082
53356	Eif3g	eukaryotic translation ini	1032.56921	1354.38574	2782.29883
110959	Nudt19	nudix (nucleoside diphos	780.128845	931.215332	2774.33228
380601	Fastkd5	FAST kinase domains 5	1007.21252	1188.00928	2759.72192
242960	Fbxl5	F-box and leucine-rich re	540.680481	544.87854	2758.37109
27756	Lsm2	LSM2 homolog, U6 sma	524.157288	723.928101	2745.51074
67841	Atg3	autophagy-related 3 (yea	1111.28638	1019.61829	2745.11328
66201	Vta1	Vps20-associated 1 hom	249.645844	325.775574	2744.073
66206	1110059E24Rik	RIKEN cDNA 1110059E	431.806458	456.127655	2741.82031
70396	Asnsd1	asparagine synthetase do	1179.54639	1309.40198	2722.58081
18120	Mrpl49	mitochondrial ribosomal	1197.3147	922.200806	2720.72217
72519	Tmem55a	transmembrane protein 5	544.978394	559.242188	2706.80518
19944	Rpl29	ribosomal protein L29	135.372894	198.039276	2701.66699
11538	Adnp	activity-dependent neuro	1033.45166	1315.81201	2694.91626
19177	Psmb7	proteasome (prosome, m	622.585083	829.758911	2688.69482
75734	Mff	mitochondrial fission fac	510.010376	729.264404	2675.43042
245474	Dkc1	dyskeratosis congenita 1,	504.516388	567.441895	2646.88672
13800	Enah	enabled homolog (Droso	50	50	2642.60278
26440	Psma1	proteasome (prosome, m	900.592896	1130.13452	2624.33618
108062	Cstf2	cleavage stimulation fact	805.50769	932.867737	2622.53174
67665	Dctn4	dynactin 4	805.85144	1175.06738	2609.17041
268996	Ss18	synovial sarcoma translo	772.904907	1094.36487	2605.06396
77809	Lrrc42	leucine rich repeat contai	550.906494	593.628296	2603.05762
109145	Gins4	GINS complex subunit 4	711.403198	1007.03711	2600.37891
66230	Mrps28	mitochondrial ribosomal	919.197632	1062.41919	2594.59277
100504952l23827	LOC100504952lB	uncharacterized LOC100	799.974121	1021.80963	2586.97461
20523	Slc25a14	solute carrier family 25 (	402.985168	565.383484	2586.49658
16906	Lmnb1	lamin B1	50	50.1319389	2535.09033
69082	Zc3h15	zinc finger CCCH-type c	618.654724	833.083496	2523.89795
17826	Fam89b	family with sequence sin	406.601868	607.929688	2514.64941
12856	Cox17	cytochrome c oxidase, su	782.324585	1067.44849	2507.72632
14168	Fgf13	fibroblast growth factor 1	50	50	2503.14014
68153	Gtf2e2	general transcription fact	558.233398	633.84021	2498.57007
80914	Uck2	uridine-cytidine kinase 2	50	68.5147858	2496.08887
68499	Mrpl53	mitochondrial ribosomal	664.805542	918.594604	2480.97217
381493	S100a7a	S100 calcium binding pr	50	50	2477.35693
101739	Psip1	PC4 and SFRS1 interacti	336.491577	309.421417	2465.69971
67383	2410127L17Rik	RIKEN cDNA 2410127L	495.022278	635.177124	2443.87891

26914	H2afy	H2A histone family, mer	556.340271	817.528625	2412.44458
22409	Wnt10a	wingless related MMTV	50	50	2394.02515
269704	Zfp664	zinc finger protein 664	478.743103	632.466553	2390.78125
103733	Tubg1	tubulin, gamma 1	482.324951	648.5802	2389.56055
20930	Surf1	surfeit gene 1	950.284363	720.951538	2364.97217
18769	Pkig	protein kinase inhibitor,	909.399414	1173.32593	2363.24512
53598	Dctn3	dynactin 3	451.686584	570.960266	2356.42725
64656	Mrps23	mitochondrial ribosomal	926.247314	1037.79028	2348.60205
69535	2310004N24Rik	RIKEN cDNA 2310004N	356.341614	414.519409	2344.23584
12042	Bcl10	B cell leukemia/lymphon	682.527954	983.119934	2335.99951
52392	D1Ertd622e	DNA segment, Chr 1, EI	350.115723	497.693481	2335.17139
14181	Fgfbp1	fibroblast growth factor b	50	71.6894073	2327.40039
16329	Inpp1	inositol polyphosphate-1-	427.014282	389.937256	2320.66797
101214	Tra2a	transformer 2 alpha homo	658.970947	860.564331	2306.82007
320438	Alg6	asparagine-linked glycos	990.07312	895.638	2305.45605
99167	Ssx2ip	synovial sarcoma, X brea	630.873657	794.283447	2303.50391
68861	1190002N15Rik	RIKEN cDNA 1190002N	810.892395	912.338806	2303.14746
67283	Slc25a19	solute carrier family 25 (	548.938904	623.589233	2273.75098
56420	Ppp4c	protein phosphatase 4, ca	452.000244	654.35791	2257.94922
74108	Parn	poly(A)-specific ribonucl	547.205811	726.583496	2257.92285
13626	Eed	embryonic ectoderm deve	657.602234	821.375244	2257.17676
20187	Ryk	receptor-like tyrosine kin	627.600708	781.720703	2256.6543
66229	Rpl7l1	ribosomal protein L7-like	870.059021	770.596802	2240.54541
17184	Matr3	matrin 3	215.574509	251.729218	2239.74854
26891	Cops4	COP9 (constitutive photo	237.835663	325.866089	2236.71973
56327	Arl2	ADP-ribosylation factor-	89.1824341	105.594536	2225.7002
100048603	LOC100048603	39S ribosomal protein L4	367.758911	274.016693	2216.33984
13006	Smc3	structural maintenance of	240.913757	247.187988	2207.92139
56443	Arpc1a	actin related protein 2/3 c	953.810791	854.234863	2207.23145
52276	Cdca8	cell division cycle associ	50	50	2191.11084
433653	Gm5549	predicted gene 5549	86.3831558	82.0281677	2188.83154
68240	Rpa3	replication protein A3	256.345428	207.783844	2185.30347
26426	Nubp2	nucleotide binding protei	691.22522	976.871094	2174.51855
28030	Gfm1	G elongation factor, mito	972.564575	810.269714	2169.61377
52575	Rg9mtd1	RNA (guanine-9-) methy	861.943176	694.634949	2168.33496
68612	Ube2c	ubiquitin-conjugating enz	50	73.2993622	2155.59326
59001	Pole3	polymerase (DNA directe	238.602417	256.348267	2148.60938
66596	Gtf3a	general transcription fact	375.494568	499.7547	2140.84326
67949	Mki67ip	Mki67 (FHA domain) int	635.535889	831.493286	2139.80469
17766	Nudt1	nudix (nucleoside diphos	680.291077	884.371216	2133.39746
69577	Fastkd3	FAST kinase domains 3	300.956451	331.83905	2116.69043
68098	Rchy1	ring finger and CHY zinc	768.18396	1035.83936	2115.31152
66421	2410004B18Rik	RIKEN cDNA 2410004B	351.082062	515.956055	2113.32495
245841|665137	Polr2h|Gm7511	polymerase (RNA) II (D	358.439087	488.367645	2108.52832
69048	Slc30a5	solute carrier family 30 (	733.466309	863.888916	2104.02246
17192	Mbd3	methyl-CpG binding dom	591.582336	611.088745	2101.83203
66310	Dpy30	dpy-30 homolog (C. eleg	565.834351	724.256287	2097.61694
67864	Yipf4	Yip1 domain family, mem	850.223267	852.108582	2096.72949
76561	Snx7	sorting nexin 7	496.048859	605.94342	2089.94775
19272	Ptprk	protein tyrosine phospha	525.367615	614.775513	2088.6958
66818	9130011J15Rik	RIKEN cDNA 9130011J	640.950684	859.538696	2083.80518
100102	Pcsk9	proprotein convertase sul	482.94278	508.892395	2079.65601
19946	Rpl30	ribosomal protein L30	206.842407	280.453644	2078.24609
18674	Slc25a3	solute carrier family 25 (	839.091064	959.014587	2076.42383
56418	Ykt6	YKT6 homolog (S. Cere	772.687866	787.495605	2069.98877
20496	Slc12a2	solute carrier family 12,	283.58255	280.563995	2069.51538
65961	Utp3	UTP3, small subunit (SS	550.607056	803.213135	2051.073
216363	Rab3ip	RAB3A interacting prote	544.042114	717.410461	2050.2793
19361	Rad51	RAD51 homolog (S. cere	50	50	2039.64233
14467	Gbas	glioblastoma amplified se	712.594482	1004.2771	2029.29297
59052	Mettl9	methyltransferase like 9	304.652405	393.676331	2028.64404

231440	Parm1	prostate androgen-regula	50	74.0816956	2018.59863
14976	H2-Ke2	H2-K region expressed g	656.550293	802.320435	2018.14014
109077	Ints5	integrator complex subur	249.715561	348.549683	2012.85376
67282	Ccdc53	coiled-coil domain conta	619.844482	583.392822	2010.68298
52443	Mrpl48	mitochondrial ribosomal	890.441284	991.086243	2010.51208
68250	Fam96a	family with sequence sin	518.064453	615.304626	2009.61816
50884	Nckap1	NCK-associated protein	400.390228	526.518372	1999.69775
22225	Usp5	ubiquitin specific peptida	758.873657	668.33252	1999.37573
70285	Rpf1	ribosome production fact	392.515167	574.661194	1988.78809
56452	Orc6	origin recognition compl	114.173157	165.909607	1985.49988
53861	Zranb2	zinc finger, RAN-binding	682.627808	984.158325	1978.08545
102209	Snapc2	small nuclear RNA activ	252.280365	365.526306	1965.46021
380863	Tmem171	transmembrane protein 1	50	55.82901	1961.8269
228410	Cstf3	cleavage stimulation fact	359.753967	341.638794	1958.18701
66194	Pycrl	pyrroline-5-carboxylate r	421.963837	470.491272	1953.68286
69617	Pitrm1	pitrilysin metallepetidase	460.219055	383.701172	1950.29773
66070	Cwc15	CWC15 homolog (S. cer	623.774841	785.775269	1913.75806
14056	Ezh2	enhancer of zeste homolo	94.5623627	121.312057	1904.24023
26886	Cenph	centromere protein H	50	50	1902.96826
66815	Ccdc109b	coiled-coil domain conta	50	50	1901.91162
230484	Usp1	ubiquitin specific peptida	97.8448334	86.7901077	1901.2478
224045	Eif2b5	eukaryotic translation ini	361.681946	479.099854	1901.00952
15975	Ifnar1	interferon (alpha and beta	733.071777	910.570374	1899.31201
54638	Ccdc22	coiled-coil domain conta	757.045532	869.919861	1893.85474
66789	Alg14	asparagine-linked glycos	559.090454	820.369385	1881.42664
60315	Myg1	melanocyte proliferating	395.309692	566.085205	1878.80884
69860	Eif1ad	eukaryotic translation ini	431.483276	604.697083	1858.20227
69085	Zcchc9	zinc finger, CCHC doma	233.113159	305.870483	1857.34778
110119	Mpi	mannose phosphate isom	355.539978	434.878601	1855.78345
12370	Casp8	caspase 8	365.865784	525.327148	1851.93652
238673	Zfp367	zinc finger protein 367	194.583893	160.122009	1847.44702
75624	Metap1	methionyl aminopeptidas	254.522003	335.75354	1845.03638
225908	Gm98	predicted gene 98	303.023834	446.155334	1843.099
329934	Foxo6	forkhead box O6	50	50	1841.65283
108664	Atp6v1h	ATPase, H+ transporting	605.151001	838.160889	1838.85107
94062	Mrpl3	mitochondrial ribosomal	323.650085	387.680786	1838.5127
226971	Plekhb2	pleckstrin homology dom	368.660339	490.662323	1832.08252
12235	Bub1	budding uninhibited by b	50	50	1825.79199
14479	Usp15	ubiquitin specific peptida	577.356201	718.098022	1821.68713
56844	Tssc4	tumor-suppressing subch	734.983948	793.571777	1816.23645
28135	Cep63	centrosomal protein 63	336.212769	481.745361	1810.28943
100972	Rab28	RAB28, member RAS on	258.626678	243.042862	1808.70703
26572	Cops3	COP9 (constitutive photo	365.73114	459.315002	1797.35693
73274	Gpbp1	GC-rich promoter bindin	532.455322	697.320068	1795.42932
108143	Taf9	TAF9 RNA polymerase	225.726044	252.050369	1791.73853
17083	Tmed1	transmembrane emp24 d	603.13269	594.052734	1780.50842
66231	Thoc7	THO complex 7 homolog	505.965942	733.522705	1779.13794
66442	Spc25	SPC25, NDC80 kinetoch	201.69693	207.011414	1770.70142
67711	Nsmce1	non-SMC element 1 hom	370.179565	536.110107	1770.55029
104303	Arl1	ADP-ribosylation factor-	413.097076	409.163269	1770.47949
11837	Rplp0	ribosomal protein, large,	537.382141	793.518066	1767.20618
98710	Rabif	RAB interacting factor	260.419983	274.22467	1759.90503
85305l631033	KarslLOC631033	lysyl-tRNA synthetasellly	417.919373	549.221741	1759.37598
21974	Top2b	topoisomerase (DNA) II	551.608276	670.689453	1757.82642
20833	Ssrp1	structure specific recogni	351.943848	495.963257	1754.69263
230393	BC057079	cDNA sequence BC0570	648.779785	662.590149	1753.89575
68087	Dcakd	dephospho-CoA kinase d	737.963867	771.265991	1746.76221
14590	Ggh	gamma-glutamyl hydrola	662.787292	755.298035	1736.12378
20530	Slc31a2	solute carrier family 31,	385.257965	563.916382	1735.38586
52551	Sgta	small glutamine-rich tetr	654.414795	814.542175	1717.03223
241576	Ldlrad3	low density lipoprotein r	364.032898	540.801331	1700.2561

21454	Tcp1	t-complex protein 1	503.580139	410.87085	1697.31458
76563	Qrsl1	glutaminyl-tRNA syntha	377.706085	534.40686	1696.18567
70083	Metrn	meteorin, glial cell differ	276.230286	300.95575	1695.01245
13445	Cdk2ap1	CDK2 (cyclin-dependent	90.0046234	112.693588	1694.72485
80751	Rnf34	ring finger protein 34	382.747009	495.632233	1687.24133
11545	Parp1	poly (ADP-ribose) polym	617.439636	790.557007	1685.03772
226162	Dpcd	deleted in primary ciliary	335.480896	360.430481	1676.16736
18567	Pdcd2	programmed cell death 2	502.628021	686.75354	1669.89502
78894	Aacs	acetoacetyl-CoA syntheta	743.053833	720.237061	1659.73462
230734	Yrdc	yrdC domain containing	261.869537	241.708771	1659.02966
50786	Hs6st2	heparan sulfate 6-O-sulfo	50	50	1658.57129
381801	Tatdn2	TatD DNase domain con	590.585876	816.106812	1641.75232
215387	Ncaph	non-SMC condensin I co	50	55.2447319	1633.90735
69109l732482	Fam58blGm9731	family with sequence sin	467.57608	542.905029	1633.00354
76459	Car12	carbonic anyhydrase 12	50	50	1628.50427
72043	Sulf2	sulfatase 2	516.291748	596.179077	1624.63269
54141	Spag5	sperm associated antigen	50	68.8783646	1613.10022
19891	Rpa2	replication protein A2	240.226227	235.989075	1612.28345
22201	Uba1	ubiquitin-like modifier ac	207.769165	213.448364	1607.80396
266692	Cpne1	copine I	145.489578	204.047577	1606.90503
216011	Lrrc20	leucine rich repeat contai	262.59668	368.013367	1604.30383
98766	Ubac1	ubiquitin associated dom	560.380005	707.174988	1599.86377
72373	Psca	prostate stem cell antigen	50	50	1598.32556
236792	Mmgt1	membrane magnesium tr	455.247864	537.860168	1592.97681
67082	1700011H14Rik	RIKEN cDNA 1700011H	50	50	1591.02295
12345	Capzb	capping protein (actin fil	183.540436	249.184158	1587.35181
72745	Tmem161b	transmembrane protein 1	319.451965	431.437988	1586.02734
231329	Polr2b	polymerase (RNA) II (D	411.398804	513.333191	1576.7561
109212	Fam64a	family with sequence sin	50	50	1563.96313
56705	Ranbp9	RAN binding protein 9	582.655396	737.847473	1553.77661
24128	Xrn2	5'-3' exoribonuclease 2	352.048401	472.263916	1551.10791
269514	Fbxl4	F-box and leucine-rich re	566.656555	679.450806	1550.79395
67724	Pop1	processing of precursor 1	139.033966	133.481461	1545.9397
17295	Met	met proto-oncogene	748.733215	755.343262	1542.11426
11938	Atp2a2	ATPase, Ca++ transporti	312.791992	400.947998	1541.50293
29805	Znhit2-ps	zinc finger, HIT domain	739.213745	655.102051	1533.47717
18221	Nudc	nuclear distribution gene	274.750641	244.908875	1530.88403
217127	Myst2	MYST histone acetyltran	304.238892	427.438599	1530.82007
72098	Tmem68	transmembrane protein 6	299.441956	430.811279	1528.11353
67967	Pold3	polymerase (DNA-direct	518.363892	688.3479	1527.80298
17349	Mlf1	myeloid leukemia factor	50	50	1520.2981
225215	Rsl24d1	ribosomal L24 domain co	511.415588	608.832275	1519.47314
66498	Dda1	DET1 and DDB1 associa	528.724548	747.394043	1503.9707
60530	Fignl1	fidgetin-like 1	50	52.8199043	1503.25415
66241	Tmem9	transmembrane protein 9	338.997803	285.225494	1497.81982
109905	Rap1a	RAS-related protein-1a	301.997253	409.93573	1495.20544
27632	Rdbp	RD RNA-binding protein	363.310486	515.936279	1494.49561
73296	Rhobtb3	Rho-related BTB domain	109.824539	154.577728	1493.70349
17344	Pias2	protein inhibitor of activa	274.680939	377.329285	1484.12317
13838	Epha4	Eph receptor A4	50	50	1472.2522
229593	Golph3l	golgi phosphoprotein 3-l	253.894684	369.59079	1462.9397
66072	Sdhaf2	succinate dehydrogenase	254.656677	379.698944	1461.77307
74112	Usp16	ubiquitin specific peptida	710.409851	717.67926	1458.05762
268697	Ccnb1	cyclin B1	50	61.1525917	1457.69287
104662	Tsr1	TSR1, 20S rRNA accum	523.843628	687.179382	1454.36523
18481	Pak3	p21 protein (Cdc42/Rac)	50	50	1449.32532
52715	Ccdc43	coiled-coil domain conta	328.128601	491.454559	1447.9696
67628	Anp32b	acidic (leucine-rich) nucl	451.152679	584.704224	1446.19324
240641	Kif20b	kinesin family member 2	50	50	1440.98413
20174	Ruvbl2	RuvB-like protein 2	292.976837	415.762939	1440.146
66492	Zmat2	zinc finger, matrin type 2	659.012146	595.250977	1438.70483

66406	Sac3d1	SAC3 domain containing	143.800827	202.216934	1428.32422
433693	Akirin2	akirin 2	448.413605	560.927124	1422.93933
231642	Alkbh2	alkB, alkylation repair ho	404.773682	589.677002	1407.60425
74493	Tnks2	tankyrase, TRF1-interact	75.1258392	111.590111	1405.33655
74051	Steap2	six transmembrane epithe	91.9769592	117.939362	1403.32031
77577	Spns3	spinster homolog 3 (Dros	50	50	1401.52246
99349	Dnajc24	DnaJ (Hsp40) homolog, s	149.877808	199.185181	1395.98804
212627	Prpsap2	phosphoribosyl pyrophos	621.124512	626.019653	1392.38428
14113|100044829	Fbl|LOC10004482	fibrillarin|rRNA 2'-O-me	111.622597	164.400116	1387.50879
69556	Bod1	biorientation of chromos	421.803833	536.824585	1381.72607
68355	2010204K13Rik	RIKEN cDNA 2010204K	50	50	1374.95752
67149	Nkain1	Na+/K+ transporting AT	50	50	1371.32251
68597	1110021J02Rik	RIKEN cDNA 1110021J	410.706543	407.683472	1371.21411
78832	2700078E11Rik	RIKEN cDNA 2700078E	655.838989	653.903809	1366.39929
72068	Cnot2	CCR4-NOT transcription	357.756287	437.17041	1364.26807
14265	Fmr1	fragile X mental retardat	258.078552	338.454254	1363.18506
73804	Kif2c	kinesin family member 2	50	50	1363.17847
72661	Serp2	stress-associated endopla	50	50	1355.89551
66506	Psmg3	proteasome (prosome, ma	647.395264	634.352417	1355.85449
242960	Fbxl5	F-box and leucine-rich re	612.129395	543.680298	1355.74597
209416	Gpkow	G patch domain and KOW	283.691833	330.748291	1353.02966
15364	Hmga2	high mobility group AT-	50	50	1352.11108
69136	Tusc1	tumor suppressor candida	438.810181	318.390747	1351.8136
98402	Sh3bp4	SH3-domain binding pro	50	60.3702507	1350.72083
14375	Xrcc6	X-ray repair complement	285.52478	297.560425	1350.35596
67149	Nkain1	Na+/K+ transporting AT	50	50	1330.88
64655	Mrps22	mitochondrial ribosomal	371.280579	401.522369	1322.49927
18969	Pola2	polymerase (DNA directe	174.355255	220.690308	1320.66211
21770	Ppp2r5d	protein phosphatase 2, re	309.244965	453.926331	1315.93604
18187	Nrp2	neuropilin 2	430.745056	393.500916	1312.26172
68276	Toe1	target of EGR1, member	322.290833	396.948608	1308.60217
20306	Ccl7	chemokine (C-C motif) l	50	50	1307.13965
65105	Arl6ip4	ADP-ribosylation factor-	426.03363	558.041077	1306.71069
106021	Topors	topoisomerase I binding,	263.044983	308.671631	1306.40186
110854	Ppp2r4	protein phosphatase 2A,	598.142456	584.207703	1305.15295
67755	Ddx47	DEAD (Asp-Glu-Ala-As	252.171082	271.283447	1295.36401
445007	Nup85	nucleoporin 85	168.273529	241.864395	1286.86499
18645	Pfn2	profilin 2	486.215759	503.601318	1264.89612
17216	Mcm2	minichromosome mainte	50	50	1259.85132
77975	Tmem50b	transmembrane protein 5	280.140076	367.354126	1257.54248
56412	Noa1	nitric oxide associated 1	240.853546	314.712463	1254.88525
231506	Lin54	lin-54 homolog (C. elega	242.228638	305.032959	1253.13037
11767	Ap1m1	adaptor-related protein co	419.452881	566.020142	1250.15918
20955	Vamp7	vesicle-associated memb	414.272552	589.852417	1247.6073
107271	Yars	tyrosyl-tRNA synthetase	574.531616	479.197449	1237.06738
78294	Rps27a	ribosomal protein S27A	136.239441	188.460205	1225.50696
76789	Mzt1	mitotic spindle organizin	282.351624	341.463379	1224.9137
108115	Slco4a1	solute carrier organic ani	50	50	1218.63477
20321	Frrs1	ferric-chelate reductase 1	141.898209	184.194839	1217.87891
13175	Dclk1	doublecortin-like kinase 1	50	50	1217.43188
78523	Mrpl9	mitochondrial ribosomal	517.836304	572.577271	1213.78052
67141	Fbxo5	F-box protein 5	50	67.7876205	1210.66492
667705	Gm8773	predicted gene 8773	50	50	1198.23022
69752	Zfp511	zinc finger protein 511	344.935364	484.137665	1197.21313
83815	Cenpq	centromere protein Q	70.0152206	95.1128922	1193.95776
70088	Meaf6	MYST/Esa1-associated f	240.031372	286.484619	1191.61121
13486	Dr1	down-regulator of transc	294.351929	351.945007	1189.26123
71835	Lancl2	LanC (bacterial lantibioti	88.2810211	132.345428	1187.55725
13605	Ect2	ect2 oncogene	50	61.0196075	1174.76758
66471	Anp32e	acidic (leucine-rich) nucl	407.881897	541.827026	1164.79297
19225	Ptgs2	prostaglandin-endoperox	50	50	1162.63696

68618	1110012L19Rik	RIKEN cDNA 1110012I	232.420868	296.479584	1160.99707
66895	1300014I06Rik	RIKEN cDNA 1300014I	423.03949	456.218201	1156.44678
69305	Dcps	decapping enzyme, scave	254.452316	306.57782	1154.87085
74268	Aven	apoptosis, caspase activa	449.95343	495.677521	1150.64941
12566	Cdk2	cyclin-dependent kinase	164.856415	189.365631	1143.97607
100503199	5430416N02Rik	RIKEN cDNA 5430416N	174.803589	206.316772	1142.45264
55988	Snx12	sorting nexin 12	254.935501	211.111267	1140.76672
14758	Gpm6b	glycoprotein m6b	50	50	1140.48242
26413	Mapk1	mitogen-activated protein	76.3757706	99.7743835	1133.19946
216527	Ccm2	cerebral cavernous malfo	357.377655	499.757568	1129.65991
16560	Kif1a	kinesin family member 1	50	50	1128.4668
52443	Mrpl48	mitochondrial ribosomal	342.245422	341.242676	1127.70435
69612	2310037I24Rik	RIKEN cDNA 2310037I	273.535553	367.672424	1127.42004
75565	Ccdc101	coiled-coil domain conta	238.5327	180.007278	1125.12939
67581	Tbc1d23	TBC1 domain family, me	65.0788574	97.3721924	1120.53149
67010	Rbm7	RNA binding motif prote	221.795654	317.864441	1116.4939
18719	Pip5k1b	phosphatidylinositol-4-ph	50	50	1114.28711
71878	Fam83d	family with sequence sim	50	54.0889053	1113.23535
319572	C730027H18Rik	RIKEN cDNA C730027I	150.913879	198.204788	1111.91577
216001	Micu1	mitochondrial calcium up	513.506714	519.77301	1108.29565
19047	Ppp1cc	protein phosphatase 1, ca	331.924377	468.056549	1107.54468
67136	Kbtbd4	kelch repeat and BTB (P	221.038406	320.896179	1106.35498
216616	Efemp1	epidermal growth factor-	58.0703659	64.437561	1105.30164
18176	Nras	neuroblastoma ras oncog	393.999573	507.152283	1102.3667
15376	Foxa2	forkhead box A2	376.22168	317.708832	1101.67493
237175	Gpr64	G protein-coupled recept	367.758911	278.314606	1100.36523
67057	Yaf2	YY1 associated factor 2	316.896637	424.507324	1095.64575
12442	Ccnb2	cyclin B2	50	50	1092.6156
66714	4921524J17Rik	RIKEN cDNA 4921524J	206.254669	261.318176	1092.42993
100859931|64113	Gm20604|Moap1	predicted gene 20604|mo	213.432678	303.147156	1092.20312
69028	Mitd1	MIT, microtubule interac	232.46048	332.533661	1089.87305
67884	1810043G02Rik	RIKEN cDNA 1810043C	121.355911	166.338272	1089.82031
74167	Nudt9	nudix (nucleoside diphos	444.204407	541.343201	1083.92603
78733	Troap	trophinin associated prote	50	65.9470673	1083.08301
70120	Yars2	tyrosyl-tRNA synthetase	287.866211	347.877686	1079.16541
19155	Npepps	aminopeptidase puromyc	386.268677	346.255005	1078.29944
108096	Slco1a5	solute carrier organic ani	50	50	1077.13599
66821	Bcs1l	BCS1-like (yeast)	316.174255	393.91687	1075.40405
59048	C1galt1c1	C1GALT1-specific chape	232.664825	279.1521	1071.78223
71701|100505160	Pnpt1|LOC100505	polyribonucleotide nucle	348.227356	455.962128	1069.36987
66929	Asf1b	ASF1 anti-silencing func	50	50	1062.59131
16434	Itpa	inosine triphosphatase (n	149.942764	208.796783	1060.82983
12028	Bax	BCL2-associated X prote	234.488251	295.278503	1059.10437
15223	Foxj1	forkhead box J1	109.925919	115.573959	1056.54578
74326	Hnrnpr	heterogeneous nuclear rib	291.08847	431.064514	1054.58215
15288	Hmbs	hydroxymethylbilane syn	412.618652	369.184753	1051.00635
24061	Smc1a	structural maintenance of	191.854309	244.311859	1050.28992
15939	Ier5	immediate early response	50	62.8502541	1050.08936
216825	Usp22	ubiquitin specific peptida	347.834473	515.757996	1048.66309
19170	Psmb1	proteasome (prosome, ma	328.576965	384.98999	1047.97119
69716	Trip13	thyroid hormone receptor	50	50	1046.15869
140494	Atp6v0a4	ATPase, H+ transporting	50	58.3641815	1040.80322
66902	Mtap	methylthioadenosine pho	208.152542	252.378586	1035.27527
59045	Stard3	START domain containi	258.143494	353.697815	1033.78162
54352	Irx5	Iroquois related homeobo	50	54.9363213	1025.93335
67453	Slc25a46	solute carrier family 25,	454.032776	500.040497	1022.21301
28010	Miip	migration and invasion in	349.428162	484.523865	1021.71832
72297	B3gnt3	UDP-GlcNAc:betaGal be	50	50	1020.46289
234549	Heatr3	HEAT repeat containing	359.235931	333.835205	1018.78345
16571	Kif4	kinesin family member 4	50	74.0590668	1015.31287
12236	Bub1b	budding uninhibited by b	50	51.2877655	1011.43146

230596	Prpf38a	PRP38 pre-mRNA proce	308.13446	307.62616	1010.97632
67204	Eif2s2	eukaryotic translation ini	234.732208	178.871262	1010.70837
16661	Krt10	keratin 10	277.510315	211.916229	1008.84827
22137	Ttk	Ttk protein kinase	50	50	1003.43689
76131	Depdc1a	DEP domain containing	50	50	994.129456
381633	Gm1673	predicted gene 1673	50	50	993.805725
67513	2610002J02Rik	RIKEN cDNA 2610002J	370.184326	472.198853	991.153503
24059	Slco2a1	solute carrier organic ani	435.009705	450.430603	988.491455
68842	Tulp4	tubby like protein 4	464.189026	414.860352	987.740417
68014	Zwilch	Zwilch, kinetochore asso	50	50	985.46283
67728	Dph2	DPH2 homolog (S. cerev	170.066833	226.078979	985.084839
70454	Cenpl	centromere protein L	122.87516	179.90683	978.957153
18642	Pfkm	phosphofructokinase, mu	407.821716	399.207886	976.85376
66398	Commd5	COMM domain containi	397.247192	376.99823	975.85791
218210	Nup153	nucleoporin 153	250.955994	261.473816	973.085754
114663	Impa2	inositol (myo)-1(or 4)-m	170.454956	146.741608	968.453369
242362	Manea	mannosidase, endo-alpha	289.559723	335.389984	966.905396
12181	Bop1	block of proliferation 1	165.792664	231.77887	966.070679
102580	Alg9	asparagine-linked glycos	264.773376	322.17511	960.215637
20873	Plk4	polo-like kinase 4 (Drosc	50	50	957.116455
14200	Fhl2	four and a half LIM dom	50	50	950.80957
75678	Ippk	inositol 1,3,4,5,6-pentaki	247.339264	241.611176	941.14386
69871	Ppp1r35	protein phosphatase 1, re	351.943848	437.466125	937.584534
12544	Cdc45	cell division cycle 45 ho	50	52.5114975	935.939575
83701	Srrt	serrate RNA effector mo	127.293488	190.014984	934.79425
80509	Med8	mediator of RNA polyme	299.601959	252.41394	931.987549
229937	Znhit6	zinc finger, HIT type 6	352.018311	450.385315	929.098694
72486	Rnf219	ring finger protein 219	227.828278	153.298828	926.51709
217578	Baz1a	bromodomain adjacent to	234.28389	345.121826	919.779663
234388	Ccdc124	coiled-coil domain conta	340.665955	438.21875	911.484497
56454	Aldh18a1	aldehyde dehydrogenase	50	50	910.797607
216705	Clint1	clathrin interactor 1	375.683075	380.682159	907.946472
192174	Rwdd4a	RWD domain containing	234.423294	243.130569	903.356812
16351	Ipp	IAP promoted placental g	298.127075	413.925232	902.717651
22218	Sumo1	SMT3 suppressor of mif	137.788803	197.146576	899.578979
67466	Pdcl	phosducin-like	209.39772	200.266037	898.68335
109115	Supt3h	suppressor of Ty 3 homo	245.720215	343.755188	895.782959
74098	0610037L13Rik	RIKEN cDNA 0610037L	372.207336	426.101685	893.212891
107368	Pdzd8	PDZ domain containing 8	200.695709	288.269958	891.144043
217198	Plekhh3	pleckstrin homology dom	215.130936	291.113556	889.213196
68080	Gpn3	GPN-loop GTPase 3	101.98909	122.315094	881.908875
11752	Anxa8	annexin A8	50	50	875.680908
67770	5830433M19Rik	RIKEN cDNA 5830433M	281.375763	257.559265	875.47052
75533	Nme5	non-metastatic cells 5, pr	50	50	871.291687
20297	Ccl20	chemokine (C-C motif) l	50	50	869.29187
236900	Pdk3	pyruvate dehydrogenase	50	50	868.494873
319801	9630033F20Rik	RIKEN cDNA 9630033F	212.949493	233.87265	867.02417
67242	Gemin6	gem (nuclear organelle) a	52.4813156	59.0913467	864.217407
102323	Dcun1d2	DCN1, defective in culli	114.900299	135.046127	859.981079
12054	Bcl7b	B cell CLL/lymphoma 7I	272.060669	403.375671	856.845703
232983	Cxcl17	chemokine (C-X-C motif	50	50	855.205688
58246	Slc35b4	solute carrier family 35,	319.112946	332.80954	854.096558
72151	Rfc5	replication factor C (acti	136.722626	172.512115	853.062866
72201	Otud6b	OTU domain containing	367.345428	313.732056	850.67688
269582	Clspn	claspin homolog (Xenop	50	50	848.096924
68436	Rpl34	ribosomal protein L34	210.498749	193.420212	847.911255
16341	Eif3e	eukaryotic translation ini	280.997131	349.763519	845.510437
217038	Mrm1	mitochondrial rRNA met	257.869446	340.768738	840.639771
54369	Nme6	non-metastatic cells 6, pr	152.288971	162.040375	839.800049
22110	Tspyl1	testis-specific protein, Y-	221.0289	247.067734	833.302551
66067	Gtpbp8	GTP-binding protein 8 (p	137.858505	201.311523	830.191895

107371	Exoc6	exocyst complex compor	118.940002	163.241455	829.227295
74035	Nol9	nucleolar protein 9	191.545395	211.71817	823.250732
11692	Gfer	growth factor, erv1 (S. ce	381.49707	317.026947	821.117676
320685	Dctd	dCMP deaminase	50	50	818.897644
75533	Nme5	non-metastatic cells 5, pr	50	50	818.212402
75939	4930579G24Rik	RIKEN cDNA 4930579G	50	50	815.851013
70427	Mier2	mesoderm induction earl	289.181091	383.899231	814.137085
13144	Dapk3	death-associated protein	149.459579	211.520111	813.612915
109093	Rars2	arginyl-tRNA synthetase	303.058655	312.716309	811.415833
20706	Serpinb9b	serine (or cysteine) pepti	50	50	807.950195
12581	Cdkn2d	cyclin-dependent kinase	50	50	807.741455
67695	Ost4	oligosaccharyltransferase	115.278931	129.050552	807.590332
106522	Pkdcc	protein kinase domain co	268.573853	228.406189	805.330811
27221	Chaf1a	chromatin assembly facto	50	50	803.00885
73162	Otud3	OTU domain containing	264.529388	318.260559	801.741943
66336	Cenpp	centromere protein P	50	69.540451	800.930176
22230	Ufd1l	ubiquitin fusion degradat	263.711975	327.265259	798.871094
19358	Rad23a	RAD23a homolog (S. ce	265.739746	379.179749	797.883545
22213	Ube2g2	ubiquitin-conjugating enz	74.4383011	88.8513489	795.684814
381802	Tsen2	tRNA splicing endonucle	160.503021	229.354065	794.085938
110173	Manba	mannosidase, beta A, lys	210.250015	306.113831	792.75647
76890	Memo1	mediator of cell motility	175.630539	237.985229	790.779663
77134	Hnrnpa0	heterogeneous nuclear rib	50	50	786.16864
14235	Foxm1	forkhead box M1	50	50	783.733276
68550	1110002N22Rik	RIKEN cDNA 1110002N	361.308044	341.077148	775.106079
19076	Prim2	DNA primase, p58 subun	50	73.5851288	772.251831
107753	Lgals2	lectin, galactose-binding,	50	50	770.84021
80385	Tusc2	tumor suppressor candida	308.587524	251.664154	770.212463
107829	Thoc5	THO complex 5	196.4263	240.189377	769.049072
13367	Diap1	diaphanous homolog 1 (D	319.974731	347.137787	766.787903
66401	Nudt2	nudix (nucleoside diphos	108.992828	119.591751	763.963135
234729	Vac14	Vac14 homolog (S. cerev	189.089874	247.947693	762.697815
78634	Spaca7	sperm acrosome associat	50	50	761.434143
103710	Slc35e4	solute carrier family 35,	50	50	760.824463
15205	Hes1	hairy and enhancer of sp	194.857956	270.546387	759.136841
268882	Fbxo45	F-box protein 45	287.80603	336.194946	753.52832
17345	Mki67	antigen identified by mon	50	69.9040375	753.21936
77116	Mtmr2	myotubularin related pro	97.9794846	119.977974	751.301697
52552	Parp8	poly (ADP-ribose) polym	50	61.7694092	749.522095
56368	Cyb561d2	cytochrome b-561 domai	188.163132	268.196533	748.103882
66855	Tcf25	transcription factor 25 (b	295.810974	270.413391	747.831116
73569	Vgll3	vestigial like 3 (Drosoph	50	50	745.813171
13684	Eif4e	eukaryotic translation ini	130.536346	173.515137	745.098389
227358	Fam132b	family with sequence sim	50	50	744.482178
69902	Mrto4	MRT4, mRNA turnover	281.280701	293.713806	738.780029
74442	Sgms2	sphingomyelin synthase 2	287.013916	307.304993	736.420288
234344|100039062	Naf1|Gm2027	nuclear assembly factor 1	182.843384	253.261368	736.250977
80385	Tusc2	tumor suppressor candida	329.936188	243.143311	734.670166
74253	Klrg2	killer cell lectin-like rece	50	50	733.286499
83945	Dnaja3	DnaJ (Hsp40) homolog,	302.819458	230.522614	732.931641
237459	Cdk17	cyclin-dependent kinase	272.478882	362.5271	732.862549
18005	Nek2	NIMA (never in mitosis	50	50	729.811035
73212	3110082I17Rik	RIKEN cDNA 3110082I	50	50	727.705994
231440	Parm1	prostate androgen-regula	50	50	727.089722
59002	Wdr8	WD repeat domain 8	219.409851	201.931152	726.711792
55988	Snx12	sorting nexin 12	179.839752	232.08728	726.439026
243958	Siglecg	sialic acid binding Ig-like	50	50	723.183716
66225	Llph	LLP homolog, long-term	252.375427	360.771423	723.078552
69549	2310009B15Rik	RIKEN cDNA 2310009B	299.367493	294.275452	721.113159
67531	5730408K05Rik	RIKEN cDNA 5730408K	179.077759	202.116486	719.857788
237211	Fancb	Fanconi anemia, complem	50	50	716.886719

69263	Rfc3	replication factor C (acti	63.1112823	87.7931442	714.213135
20133	Rrm1	ribonucleotide reductase	50	50	714.132568
68187	Fam135a	family with sequence sin	174.83844	231.061615	711.823792
15944	Irgm1	immunity-related GTPas	324.532471	338.795197	706.760864
13590	Lefty1	left right determination f	50	72.5495605	705.551392
74340	Ahcyl2	S-adenosylhomocysteine	264.355133	347.74472	705.208008
71735	Lrwd1	leucine-rich repeats and	98.5371246	133.614441	705.122559
71517	9030624J02Rik	RIKEN cDNA 9030624J	203.559952	290.36377	704.696899
20348	Sema3c	sema domain, immunogl	50	50	704.272949
72170	Chchd4	coiled-coil-helix-coiled-c	259.806915	332.501129	704.222046
116748	Lsm10	U7 snRNP-specific Sm-l	138.102463	124.035385	700.186157
71514	Sfpq	splicing factor proline/gl	168.861267	225.595154	699.721069
229927	Clca4	chloride channel calcium	50	50	699.088379
24105	Rbck1	RanBP-type and C3HC4-	111.826958	161.092499	697.478027
76843	Dtl	denticleless homolog (Dr	50	50	697.157593
98386	Lbr	lamin B receptor	50	50	697.123047
226245	9930023K05Rik	RIKEN cDNA 9930023K	50	50	693.414185
213556	Plekhh2	pleckstrin homology don	117.903946	139.356781	693.146362
69216	Ccdc23	coiled-coil domain conta	272.260284	343.511902	692.571228
214791	Sertad4	SERTA domain containi	50	50	691.274658
114863	Prosc	proline synthetase co-tra	153.325027	155.162003	690.9953
228421	Kif18a	kinesin family member 1	50	50	687.565796
56031	Ppie	peptidylprolyl isomerase	129.196106	159.61554	687.565796
27784	Commd8	COMM domain containi	179.461121	185.386032	684.136353
208084	Pif1	PIF1 5'-to-3' DNA helica	50	50	684.065674
72065	Rap2c	RAP2C, member of RAS	260.838196	310.830475	682.450317
17967	Ncam1	neural cell adhesion mol	50	50	681.096313
26909	Exo1	exonuclease 1	50	50	679.380676
67832	Brix1	BRX1, biogenesis of rib	134.058014	156.165039	677.167236
22722	Zfp64	zinc finger protein 64	113.311356	148.780212	675.172241
65964	B230120H23Rik	RIKEN cDNA B230120I	138.17218	203.275146	675.090088
24058	Sigirr	single immunoglobulin a	184.01886	138.860214	674.207642
77022	2700099C18Rik	NDC80 homolog, kineto	50	65.2199097	671.944885
67674	Trmt112	tRNA methyltransferase	150.809326	200.210861	671.563599
66980	Zdhhc6	zinc finger, DHHC doma	186.63913	232.054749	671.241577
68642	Tmem216	transmembrane protein 2	228.455627	188.603104	670.888306
73998	Herc3	hect domain and RLD 3	228.102341	314.819977	668.439819
66355	Gmpr	guanosine monophospha	50	50	664.911743
320213	Senp5	SUMO/sentrin specific p	294.461243	301.053345	663.342346
108121	U2af1	U2 small nuclear ribonuc	115.423088	168.30191	663.273315
98376	Gorab	golgin, RAB6-interacting	109.78968	118.997574	661.842041
105351	AW209491	expressed sequence AW2	251.752838	325.502502	658.261353
71707	Ubiad1	UbiA prenyltransferase d	158.544952	200.554657	657.00592
66278	1810013D10Rik	RIKEN cDNA 1810013D	323.909912	277.123413	656.476807
16497	Kcnab1	potassium voltage-gated	50	50	655.732422
67674	Trmt112	tRNA methyltransferase	183.226776	174.638428	651.808228
20729	Spin1	spindlin 1	50	50	646.681274
100336	Ppp1r8	protein phosphatase 1, re	206.59845	199.626587	645.414307
215999	Ccdc109a	coiled-coil domain conta	50	51.5636368	643.095581
19881	Rom1	rod outer segment memb	201.278702	161.2453	642.275635
108961	E2f8	E2F transcription factor 8	135.407745	169.954285	641.753052
241846	Lsm14b	LSM14 homolog B (SCI	80.1018066	96.148468	641.613403
68051	Nutf2	nuclear transport factor 2	139.412598	187.457184	638.699829
229782	Slc35a3	solute carrier family 35 (	314.585327	306.434937	638.560181
57434	Xrcc2	X-ray repair complement	92.7342072	111.226532	638.11322
233726	Ipo7	importin 7	156.707291	168.675385	636.356567
107746	Rapgef1	Rap guanine nucleotide e	212.431458	312.39801	636.008179
100087	Kti12	KTI12 homolog, chroma	253.520813	203.285034	635.840576
237211	Fancb	Fanconi anemia, comple	50	50	631.195068
231876	Lmtk2	lemur tyrosine kinase 2	178.220703	244.256683	630.695496
328801	Zfp414	zinc finger protein 414	230.453308	284.686493	630.370117

22158	Tulp3	tubby-like protein 3	50	71.2154846	628.631592
19012	Ppap2a	phosphatidic acid phosph	50	50	626.615234
68449	Tbc1d10b	TBC1 domain family, me	222.752502	283.881531	621.463623
230376	Haus6	HAUS augmin-like comp	57.1737061	51.9922943	619.935364
15247	Hiat1	hippocampus abundant g	170.171387	242.922607	619.626465
234686	Fhod1	formin homology 2 doma	194.33992	262.376404	617.697266
107823	Whsc1	Wolf-Hirschhorn syndro	212.440964	300.669983	611.211243
14048	Eya1	eyes absent 1 homolog (I	50	50	610.249939
330122	Cxcl3	chemokine (C-X-C motif	50	50	607.482666
56217	Mpp5	membrane protein, palmi	50	50	606.495056
67052	Ndc80	NDC80 homolog, kineto	50	50	606.19928
214580	Pstk	phosphoseryl-tRNA kina	120.658859	173.690567	605.656982
23825	Banf1	barrier to autointegration	50	50	603.519104
67573	Loxl4	lysyl oxidase-like 4	50	50	600.543091
433813	Pusl1	pseudouridylate synthase	125.08194	178.916534	600.472473
212307	Mapre2	microtubule-associated p	192.551361	236.297485	598.949158
78895	Pus7l	pseudouridylate synthase	128.503815	175.586288	598.24585
272551	Gins2	GINS complex subunit 2	50	50	598.124268
69770	1600002K03Rik	RIKEN cDNA 1600002K	106.990402	160.066833	597.979614
109065	1110034A24Rik	RIKEN cDNA 1110034A	78.5429535	75.3705063	595.1828
76832	Hyls1	hydrolethalus syndrome	99.8076477	140.028763	594.165649
66190	Acer3	alkaline ceramidase 3	204.252243	274.974457	594.025879
70454	Cenpl	centromere protein L	128.264603	164.156784	592.87561
20346	Sema3a	sema domain, immunoglo	50	50	590.944763
66664	Tmem41a	transmembrane protein 4	205.278809	255.234894	590.729492
12545	Cdc7	cell division cycle 7 (S. c	50	50	590.660522
432508	Cpsf6	cleavage and polyadenyla	63.2807922	73.2993622	589.166748
218581	Depdc1b	DEP domain containing	50	50	588.805298
18617	Rhox5	reproductive homeobox 5	50	50	587.235962
66973	Mrps18b	mitochondrial ribosomal	88.24617	100.491653	586.672241
229672	Bcl2l15	BCLl2-like 15	50	50	584.835083
56470	Rgs19	regulator of G-protein si	50	50	584.835083
22422	Wnt7b	wingless-related MMTV	50.9620705	52.9627914	584.411133
105785	Kdelr3	KDEL (Lys-Asp-Glu-Le	50	53.4692612	583.242798
212377	Mms22l	MMS22-like, DNA repai	75.574173	110.554535	581.504211
20419	Shcbp1	Shc SH2-domain binding	50	50	578.487183
11781	Ap4m1	adaptor-related protein co	116.210434	160.122009	577.783813
70784	Rasl12	RAS-like, family 12	50	50	576.569458
544717|100503277	1190007I07Rik|Gn	RIKEN cDNA 1190007I	207.769165	220.833206	575.813599
108899	2700081O15Rik	RIKEN cDNA 2700081O	121.216499	168.532501	575.701782
272396	Tarsl2	threonyl-tRNA synthetas	263.358673	249.778336	575.121704
50760	Fbxo17	F-box protein 17	50	55.5305023	574.807861
381318	Nsl1	NSL1, MIND kinetochor	50	50	574.516968
108689	Obfc1	oligonucleotide/oligosacc	50	50	573.83667
70355	Gprc5c	G protein-coupled recept	56.585968	70.5434875	571.76123
16571	Kif4	kinesin family member 4	50	50	571.628174
72614	Pih1d2	PIH1 domain containing	50	50	569.749878
225997	Trpm6	transient receptor potenti	127.811516	140.480072	568.668579
22036	Traip	TRAF-interacting protein	50	68.9561768	567.942261
329369	5730588L14Rik	RIKEN cDNA 5730588L	50	53.9983635	567.855225
12224	Klf5	Kruppel-like factor 5	50	50	567.727051
71971	Zswim1	zinc finger, SWIM doma	80.1319122	115.592346	566.675293
23797	Akt3	thymoma viral proto-onc	50	67.7324448	564.709961
237459	Cdk17	cyclin-dependent kinase	157.952469	190.080063	564.59491
223828	Pphln1	periphilin 1	50	50	563.896545
26885	Casp8ap2	caspase 8 associated prot	140.000336	156.385742	561.66333
226043	Cbwd1	COBW domain containin	50	67.4565735	561.23938
13481	Dpm2	dolichol-phosphate (beta-	138.959518	201.720367	560.320801
241035	Pkhd1	polycystic kidney and he	168.378082	229.45166	559.757141
27966	Rrp9	RRP9, small subunit (SS	50	50	559.292114
50883	Chek2	checkpoint kinase 2	50	66.586525	556.181396

21956	Tnnt2	troponin T2, cardiac	50	50	555.961182
67884	1810043G02Rik	RIKEN cDNA 1810043G	94.7017822	132.929718	551.75769
114715	Spred1	sprouty protein with EVI	103.364174	121.510117	551.49646
80905	Polh	polymerase (DNA directe	85.2773895	99.245285	550.955811
114863	Prosc	proline synthetase co-tra	104.644211	154.775787	550.921265
13836	Epha2	Eph receptor A2	185.777328	174.97937	549.613281
18393	Orc2	origin recognition compl	50	50	548.600952
235169	Foxred1	FAD-dependent oxidored	167.307159	205.787674	546.962646
69962	2810422O20Rik	RIKEN cDNA 2810422O	216.680267	203.814148	546.177124
66549	Aggf1	angiogenic factor with G	195.524902	225.221649	538.225342
69257	Elf2	E74-like factor 2	232.211761	258.552429	536.527893
66580	Esf1	ESF1, nucleolar pre-rRN	110.581772	133.744598	536.108826
56401	Lepre1	leprecan 1	246.955887	238.777496	535.783447
100088	Rcc1	regulator of chromosome	99.0155487	137.38324	535.405518
52679	E2f7	E2F transcription factor 7	50	50	534.876404
12563	Cdh6	cadherin 6	50	50	534.505005
30938	Fgd3	FYVE, RhoGEF and PH	50	50	534.085938
51812	Mcrs1	microspherule protein 1	50	51.6612473	532.638245
66748	4933404M02Rik	RIKEN cDNA 4933404M	50	50	532.493652
15476	Hs3st1	heparan sulfate (glucosar	50	50	531.934937
208624	Alg3	asparagine-linked glycos	176.562042	264.207031	530.679443
215707	Ccdc92	coiled-coil domain conta	50	50	524.586243
108013	Celf4	CUGBP, Elav-like family	50	50	517.960571
20017	Polr1b	polymerase (RNA) I poly	187.505692	226.753799	517.715698
110012	Gm16517	predicted gene, Gm1651	175.491119	246.726776	516.309082
233875	Ino80e	INO80 complex subunit	61.0042992	88.3448792	514.996094
22137	Ttk	Ttk protein kinase	50	50	514.91394
70466	Ckap2l	cytoskeleton associated p	50	50	514.739746
11920	Atm	ataxia telangiectasia muta	208.531158	254.572815	514.251709
68588	Cthrc1	collagen triple helix repe	50	50	509.798431
66129	1110018J18Rik	RIKEN cDNA 1110018J	235.354797	238.767578	509.001465
17760	Mtap6	microtubule-associated p	50	50	508.845337
72454	Ccdc71	coiled-coil domain conta	153.808197	200.73996	508.276794
76932	Arfip2	ADP-ribosylation factor	134.854858	196.409515	507.979309
68193	Rpl24	ribosomal protein L24	50	58.925827	507.938263
234814	Mthfsd	methenyltetrahydrofolate	193.478119	224.504395	507.560303
30953///100505386	Schip1///Iqcj-schip1	schwannomin interacting	50	50	506.7995
269623	C030048B08Rik	RIKEN cDNA C030048B	112.066177	160.375244	505.798737
225895	Taf6l	TAF6-like RNA polymer	63.9730911	79.1520462	502.996948
207521	Dtx4	deltex 4 homolog (Droso	177.010376	239.053345	501.340515
71116	Stx18	syntaxin 18	102.367706	84.9170227	500.591187
22375	Wars	tryptophanyl-tRNA synth	50	50	500.270752
22268	Upk1b	uroplakin 1B	53.2433167	64.7558746	497.881439
83767	Wasf1	WASP family 1	96.3255768	113.839508	497.643188
66464	Taf12	TAF12 RNA polymerase	197.034637	197.234283	497.2948
65962	Slc9a3r2	solute carrier family 9 (s	74.1246338	99.8295593	495.59729
216274	Cep290	centrosomal protein 290	50	73.5525894	495.178284
66949	Trim59	tripartite motif-containin	50	50	492.312408
22720	Zfp62	zinc finger protein 62	172.691849	213.448364	492.172729
74246	Gale	galactose-4-epimerase, U	111.483185	161.874847	489.923096
17760	Mtap6	microtubule-associated p	50	50	488.795776
67196	Ube2t	ubiquitin-conjugating en	50	50	486.371948
13732	Emp3	epithelial membrane prot	50	51.4108467	484.947266
30937	Lmcd1	LIM and cysteine-rich do	50	57.8902512	483.384521
77552	Shisa4	shisa homolog 4 (Xenopu	50	50	479.756165
218343	Ttc37	tetratricopeptide repeat d	193.034546	216.934235	477.541016
54200	Sult2b1	sulfotransferase family, c	50	50	477.483521
26379	Esrra	estrogen related receptor,	183.675095	231.801514	477.227173
57895	Ccdc126	coiled-coil domain conta	118.940002	145.495239	474.021149
232946	Bloc1s3	biogenesis of lysosome-r	154.191589	176.534149	473.582397
70967	4931408A02Rik	RIKEN cDNA 4931408A	50	50	473.529816

218973	Wdhd1	WD repeat and HMG-bo	50	70.4883118	471.421509
94353	Hmgn3	high mobility group nucl	50	50	470.164368
20355	Sema4f	sema domain, immunogl	50	50	469.786438
68327	0610007P22Rik	RIKEN cDNA 0610007P	158.300995	168.973907	469.48407
232187	Smyd5	SET and MYND domain	160.921265	206.306885	468.315735
70218	Kif18b	kinesin family member 1	50	50	466.659302
234086	Erich1	glutamate-rich 1	50	69.2971191	465.472839
210530	Leprel1	leprecan-like 1	50	50	465.362762
14391	Gabpb1	GA repeat binding prote	69.0140076	85.9851379	464.50824
66440	Cdc26	cell division cycle 26	148.841751	109.551498	464.258484
100039781	Hrct1	histidine rich carboxyl te	50	60.7564697	464.031708
105988	Espl1	extra spindle poles-like 1	50	50	461.589813
67706	Tmem179b	transmembrane protein 1	135.925781	163.241455	459.589935
209003	Rbmx2	RNA binding motif prote	50	50	458.648346
100206	Adprhl2	ADP-ribosylhydrolase lik	126.740601	125.093597	457.793854
67203	Nde1	nuclear distribution gene	50	50	456.67807
18760	Prkd1	protein kinase D1	50	50	455.52121
13017	Ctbp2	C-terminal binding prote	50	50	455.230347
12448	Ccne2	cyclin E2	50	50	454.323242
208117	Aph1b	anterior pharynx defectiv	81.9949265	118.027077	454.183594
225651	Mppe1	metallophosphoesterase	114.865448	172.025436	453.207458
26951	Zw10	ZW10 homolog (Drosop	136.443817	148.05304	452.568237
54722	Dfna5	deafness, autosomal dom	106.75119	149.793152	452.527161
20349	Sema3e	sema domain, immunogl	50	50	451.143524
68092	Ncbp2	nuclear cap binding prote	76.4407272	109.463791	450.23645
75317	4930547N16Rik	RIKEN cDNA 4930547N	50	50	449.748413
232313	Gxylt2	glucoside xylosyltransfer	50	50	449.446045
78906	9130017N09Rik	RIKEN cDNA 9130017N	50	50	447.71405
69185	Dtwd1	DTW domain containing	160.403229	171.707123	446.504578
624086	A230045G11Rik	RIKEN cDNA A230045	50	60.8767242	444.568817
66374	2310011J03Rik	RIKEN cDNA 2310011J	125.774246	170.626282	443.045502
623474	Rad54b	RAD54 homolog B (S. c	50	70.1799088	442.587036
241627	Wdr76	WD repeat domain 76	63.0067291	91.9156342	442.02832
75572	Acyp2	acylphosphatase 2, musc	148.184296	105.120605	441.837708
215707	Ccdc92	coiled-coil domain conta	50	50	441.278992
57785	Rangrf	RAN guanine nucleotide	50	68.3167191	440.546082
72672	Zfp518a	zinc finger protein 518A	152.672333	156.55127	437.389343
102920	Cenpi	centromere protein I	50	50	437.366333
218271	B4galt7	xylosylprotein beta1,4-ga	50	72.6047287	429.007019
67564	Tmem35	transmembrane protein 3	50	52.345974	428.541992
13593	Ebf3	early B cell factor 3	50	50	428.408875
67870	Enoph1	enolase-phosphatase 1	50	50	427.989868
19362	Rad51ap1	RAD51 associated prote	50	50	427.380188
26934	Racgap1	Rac GTPase-activating p	50	50	426.693298
228482	Arhgap11a	Rho GTPase activating p	50	50	425.815796
219072	Haus4	HAUS augmin-like comp	154.156738	148.747681	425.391846
236899	Pcyt1b	phosphate cytidylyltransf	50	52.9076157	424.001648
212898	Dse	dermatan sulfate epimera	110.238007	149.838425	422.553925
232157	Mob1a	MOB kinase activator 1A	50	50	422.129944
241308	Ralgps1	Ral GEF with PH domai	50	50	421.827576
240756	Klhl12	kelch-like 12 (Drosophila	196.869873	209.228271	421.479218
232164	Paip2b	poly(A) binding protein i	209.985458	195.283401	421.101257
58186	Rad18	RAD18 homolog (S. cere	81.0285645	119.935532	421.071655
74201	Cep97	centrosomal protein 97	50	50	419.729126
100647	Upk3b	uroplakin 3B	50	50	419.531921
75623	1700029F09Rik	RIKEN cDNA 1700029F	50	67.566925	418.665924
215708	Fam73a	family with sequence sin	162.510223	180.435944	413.282593
18760	Prkd1	protein kinase D1	50	50	412.410004
18783	Pla2g4a	phospholipase A2, group	50	50	412.224304
207704	Gtpbp10	GTP-binding protein 10 (	122.531387	142.531403	411.282715
67049	Pus3	pseudouridine synthase 3	184.36264	199.38324	410.91626

226841	Vash2	vasohibin 2	50	50	409.323944
69776	1600002D24Rik	RIKEN cDNA 1600002I	50	50	408.865479
226154	Lzts2	leucine zipper, putative t	50	50	406.612549
71874	2310007B03Rik	RIKEN cDNA 2310007I	50	50	405.993042
217219	Fam171a2	family with sequence sin	50	67.5018463	404.336609
74732	Stx11	syntaxin 11	50	54.5501022	404.178864
76789	Mzt1	mitotic spindle organizin	122.287422	159.021362	404.114777
16975	Lrp8	low density lipoprotein r	50	50	403.940582
224171	C330027C09Rik	RIKEN cDNA C330027	50	50	403.033508
102124	E130303B06Rik	RIKEN cDNA E130303I	50	50	402.28418
15369	Hmox2	heme oxygenase (decycli	50	51.0217972	401.441162
14573	Gdnf	glial cell line derived neu	50	50	395.877045
231872	Aimp2	aminoacyl tRNA synthet	50	50	395.104706
18704	Pik3c2a	phosphatidylinositol 3-ki	50	50	394.14505
269252	Gtf3c4	general transcription fact	123.881126	144.271515	393.709595
52184	Odf2l	outer dense fiber of sperm	83.6535797	109.915085	393.709595
70078	Nol7	nucleolar protein 7	149.076202	176.754852	393.656982
270906	Prr11	proline rich 11	50	50	393.256042
27078	B9d1	B9 protein domain 1	50	61.3506546	393.203461
17151	Ccndbp1	cyclin D-type binding-pr	122.14801	117.322556	392.726929
67689	Aldh3b1	aldehyde dehydrogenase	50	58.484436	392.511658
380840	Lyrm4	LYR motif containing 4	147.487259	188.736084	391.308777
12237	Bub3	budding uninhibited by b	125.086693	138.19812	390.919312
68865	Arv1	ARV1 homolog (yeast)	68.1870575	100.524185	389.668762
76179	Usp31	ubiquitin specific peptida	116.593811	146.917023	388.617065
68559	Pdrg1	p53 and DNA damage re	87.3146591	115.371651	388.099457
379043	Raet1e	retinoic acid early transc	50	50	387.838196
12532	Cdc25c	cell division cycle 25 ho	50	50	387.634399
14697	Gnb5	guanine nucleotide bindi	50	61.1525917	385.524445
319953	Ttll1	tubulin tyrosine ligase-lik	50	55.4102516	385.396301
216049	Zfp365	zinc finger protein 365	50	50	383.687256
67838	Dnajb11	DnaJ (Hsp40) homolog,	101.710274	102.530258	381.449158
100210	Gpn2	GPN-loop GTPase 2	131.886078	132.147369	380.839478
13800	Enah	enabled homolog (Droso	50	50	379.961975
232539	Klhdc5	kelch domain containing	143.651917	144.326691	379.815735
26896	Med14	mediator complex subuni	123.806664	181.020218	379.653046
13527	Dtna	dystrobrevin alpha	50	50	377.879944
67121	Mastl	microtubule associated se	50	65.4505005	377.217712
14659	Glrp1	glutamine repeat protein	50	50	377.013947
210719	Mkx	mohawk homeobox	121.181648	135.763397	375.456116
75219	Dusp18	dual specificity phosphat	50	50.8463745	374.688721
70134	2210011C24Rik	RIKEN cDNA 2210011C	50	50	374.549042
20210	Saa3	serum amyloid A 3	50	50	373.055298
67979	Atad1	ATPase family, AAA do	131.402893	102.387375	372.310913
64297	Gprc5b	G protein-coupled recept	50	50	371.758759
66607	Ms4a4d	membrane-spanning 4-do	50	50	371.183655
224170	Dzip3	DAZ interacting protein	50	73.9161758	369.735901
56075	Pdss1	prenyl (solanesyl) diphos	50	69.2971191	368.957001
53886	Cdkl2	cyclin-dependent kinase-	50	59.3997574	366.741882
66078	Tsen34	tRNA splicing endonucle	50	50	366.66626
218454	Lhfpl2	lipoma HMGIC fusion pa	95.3893204	117.741302	366.62027
237211	Fancb	Fanconi anemia, comple	50	50	366.184814
216795	Wnt9a	wingless-type MMTV in	50	50	366.091125
75212	Rnf121	ring finger protein 121	125.983353	121.136627	366.068115
11416	Slc33a1	solute carrier family 33 (	123.218925	116.936325	365.823273
321000	4933421E11Rik	RIKEN cDNA 4933421I	170.176147	175.985229	365.305664
77951	Cyp20a1	cytochrome P450, family	50	50	364.579346
21778	Tex9	testis expressed gene 9	50	50	363.736328
15893	Ica1	islet cell autoantigen 1	50	55.1895561	363.573639
21987	Tpd52l1	tumor protein D52-like 1	50	50	362.707642
60527	Fads3	fatty acid desaturase 3	50	50	361.899139

330695	Ctxn1	cortexin 1	50	50	361.894226
245670	Rragb	Ras-related GTP binding	50	50	361.754547
72137	Wdsub1	WD repeat, SAM and U-	89.1079712	128.047531	361.701965
72433	Rab38	RAB38, member of RAS	50	50	361.614868
229841	Cenpe	centromere protein E	50	50	361.399597
56513	Pard6a	par-6 (partitioning defect	74.5032501	77.3440399	361.358521
210094	Iglon5	IgLON family member 5	81.7905579	82.437027	361.213928
76804	Kdm4c	lysine (K)-specific deme	115.966469	157.531647	361.115295
68277	2310057M21Rik	RIKEN cDNA 2310057M	50	50	360.678192
223690	Ankrd54	ankyrin repeat domain 54	89.003418	107.821304	360.533569
218756	Slc4a7	solute carrier family 4, so	109.032433	135.26683	360.068542
381822	1190002F15Rik	RIKEN cDNA 1190002F	50	50	359.731689
76547	Tmem101	transmembrane protein 1	83.9624939	123.736877	357.592133
404710	Iqgap3	IQ motif containing GTP	50	50	357.429443
68895	Rasl11a	RAS-like, family 11, me	50	50	357.104065
68214	Gsto2	glutathione S-transferase	50	50	356.162476
67391	Fundc2	FUN14 domain containin	64.1124954	94.2527466	355.871643
20197	S100a3	S100 calcium binding pr	50	50	355.807556
20589	Ighmbp2	immunoglobulin mu bind	142.346527	147.722015	355.796021
381306	BC055324	cDNA sequence BC0553	50	50	355.633362
71233	Enkur	enkurin, TRPC channel i	50	50	355.528198
74136	Sec14l1	SEC14-like 1 (S. cerevis	50	59.8736877	355.051636
230157	Tmeff1	transmembrane protein w	50	50	354.545532
30056	Timm9	translocase of inner mito	160.477692	151.006989	354.26123
77593	Usp45	ubiquitin specific petidas	116.110634	88.1793594	353.755096
94180	Acsbg1	acyl-CoA synthetase bub	50	50	353.331116
66405	Mcts2	malignant T cell amplifie	112.828171	137.470963	351.743744
56496	Tspan6	tetraspanin 6	50	50	350.215485
242466	Zfp462	zinc finger protein 462	50	50	349.796448
232431	Gprc5a	G protein-coupled recept	50	50	348.12854
72119	Tpx2	TPX2, microtubule-asso	50	50	346.053101
19872	Rny1	RNA, Y1 small cytoplas	50	50	344.215912
103551	E130012A19Rik	RIKEN cDNA E130012A	50	50	344.064728
69757	Leng1	leukocyte receptor cluste	111.617844	157.664642	343.936554
27401	Skp2	S-phase kinase-associate	50	50	343.78537
67994	Mrps11	mitochondrial ribosomal	140.348862	166.802307	342.745178
100048499	LOC100048499	uncharacterized LOC100	110.860596	99.8295593	339.879333
18974	Pole2	polymerase (DNA direct	50	50	338.646851
64406	Sp5	trans-acting transcription	50	50	338.559753
319482	9530053A07Rik	RIKEN cDNA 9530053A	50	50	338.042145
56419	Diap3	diaphanous homolog 3 (I	50	50	336.786682
15394	Hoxa1	homeobox A1	50	50	336.292053
232664	Ccdc136	coiled-coil domain conta	50	50	335.130249
75317	4930547N16Rik	RIKEN cDNA 4930547N	50	50	334.990601
234825	Klhdc4	kelch domain containing	115.557739	121.246971	332.124725
68281	4930430F08Rik	RIKEN cDNA 4930430F	50	71.0499573	330.502808
269033	4930503L19Rik	RIKEN cDNA 4930503L	50	50	329.723907
21384	Tbx15	T-box 15	50	50	328.956512
268783	Mtmr12	myotubularin related pro	107.647842	141.252502	327.758545
56220	Zfp386	zinc finger protein 386 (I	50	72.1534424	327.607361
66362	Exosc3	exosome component 3	149.494446	156.275391	327.125916
73680	Zbtb8a	zinc finger and BTB dom	50	50	325.753784
93737	Pard6g	par-6 partitioning defecti	50	50	325.375793
71306	Mfap3l	microfibrillar-associated	50	50	323.119598
66140	Fam33a	family with sequence sin	50	50	321.806641
243339	Tmem130	transmembrane protein 1	50	50	320.934052
66437	Fis1	fission 1 (mitochondrial	50	52.6317444	320.492004
18162	Npr3	natriuretic peptide recept	50	50	320.086121
70681	Fam175a	family with sequence sin	132.857178	114.732208	319.765686
319765	Igf2bp2	insulin-like growth facto	50	50	319.126465
105377	Ankrd32	ankyrin repeat domain 32	50	66.8397598	318.771484

214901	Chtf18	CTF18, chromosome tra	50	50	317.603149
217653	Mis18bp1	MIS18 binding protein 1	50	50	317.080566
22145	Tuba4a	tubulin, alpha 4A	134.027924	128.190414	316.801239
208628	Kntc1	kinetochore associated 1	50	50	315.795532
20539	Slc7a5	solute carrier family 7 (c	50	50	314.975525
232827	Nlrp2	NLR family, pyrin doma	50	50	314.784943
242620	Dmrta2	doublesex and mab-3 rel	50	50	314.673187
268490	Lsm12	LSM12 homolog (S. cere	79.2003937	94.3178177	314.056976
66682	Trappc5	trafficking protein partic	114.482071	136.633438	313.656006
77634	Snapc3	small nuclear RNA activ	50	50	312.65033
66098	Chchd6	coiled-coil-helix-coiled-c	50	50	312.366028
100342	Fam46b	family with sequence sin	50	50	310.755646
57810	Cdon	cell adhesion molecule-r	50	50	310.638977
239839	Ccdc14	coiled-coil domain conta	50	57.1404533	310.400696
232946	Bloc1s3	biogenesis of lysosome-r	89.0731201	113.200058	308.982544
12297	Cacnb3	calcium channel, voltage	50	50	308.505981
67030	Fancl	Fanconi anemia, comple	50	71.9652786	308.377808
270669	Mbtps2	membrane-bound transcr	50	50	307.871704
53885	Nphp1	nephronophthisis 1 (juve	50	58.2212982	307.569336
193385	Fam65b	family with sequence sin	50	74.3349304	307.104279
83675	Bicc1	bicaudal C homolog 1 (D	50	50	306.488068
66060	0610010O12Rik	RIKEN cDNA 0610010O	50	52.9076157	306.465027
11636	Ak1	adenylate kinase 1	50	50	306.128174
22059	Trp53	transformation related pr	50	63.9410019	305.447876
24116	Whsc2	Wolf-Hirschhorn syndro	90.0046234	102.221848	302.332214
19249	Ptpn13	protein tyrosine phospha	50	50	301.931274
71436	Flrt3	fibronectin leucine rich t	50	50	301.546753
18108	Nmt2	N-myristoyltransferase 2	50	50	301.285461
101985	AA960436	expressed sequence AA9	73.850563	93.9995117	301.012695
100040736	9130206I24Rik	RIKEN cDNA 9130206I	50	50	300.593628
78833	Gins3	GINS complex subunit 3	50	50	300.442474
13732	Emp3	epithelial membrane prot	50	50	300.192688
21939	Cd40	CD40 antigen	50	50	298.350586
100862531l70359	LOC100862531lG	tRNA modification GTP	129.848801	140.953995	297.844452
17245	Mdm1	transformed mouse 3T3 c	50	50	295.525818
67877	Naa20	N(alpha)-acetyltransfera	50	63.4996109	294.437958
11834	Aqr	aquarius	57.8612518	68.2290115	294.13562
103743	Tmem98	transmembrane protein 9	52.5858727	55.9945297	294.083008
330959	Snapc5	small nuclear RNA activ	104.335289	135.851105	294.071503
67222	Srfbp1	serum response factor bi	50	57.2182617	293.542358
71793	Ints12	integrator complex subun	61.6316414	79.0417023	292.461121
94220	Cnnm4	cyclin M4	50	55.6733894	291.833374
67486	Polr3g	polymerase (RNA) III (D	87.628334	65.2750778	289.572235
100043314	Tigit	T cell immunoreceptor w	50	50	289.258362
26895	Cops7b	COP9 (constitutive photo	117.934044	126.184341	288.642151
14299	Ncs1	neuronal calcium sensor	50	50	288.154114
240261	Ccdc112	coiled-coil domain conta	50	50	288.147522
28081	D11Wsu99e	DNA segment, Chr 11, W	78.8613815	98.2097092	286.846039
67008	1600012F09Rik	RIKEN cDNA 1600012F	90.970993	78.5125961	286.676788
621080	AI429214	expressed sequence AI42	72.709938	96.589859	286.630798
54635	Pdgfc	platelet-derived growth f	50	61.5161743	286.392517
14221	Fjx1	four jointed box 1 (Dros	50	50	286.275818
233826	Palb2	partner and localizer of B	50	56.2251282	285.851868
14697	Gnb5	guanine nucleotide bindi	50	50	285.810791
17231	Mcpt8	mast cell protease 8	50	50	285.746704
75659	Wdr54	WD repeat domain 54	50	50	285.648102
66959	Dusp26	dual specificity phospha	50	50	285.531433
12571	Cdk6	cyclin-dependent kinase	50	50	284.566833
72930	Ppp2r2b	protein phosphatase 2 (fo	50	73.1338348	284.555328
12816	Col12a1	collagen, type XII, alpha	50	51.6739845	284.520813
69917	Obfc2b	oligonucleotide/oligosac	92.2510223	115.40419	284.037689

67538	Zswim3	zinc finger, SWIM doma	107.169418	132.037033	282.71814
22174	Tyro3	TYRO3 protein tyrosine	50	50	279.160461
16551	Kif11	kinesin family member 1	50	50	276.684082
101631	Pwwp2b	PWWP domain containin	50	50	276.672546
77591	Ddx10	DEAD (Asp-Glu-Ala-As	50	73.6855774	276.585449
242642	Hpdl	4-hydroxyphenylpyruvat	50	50	275.905151
228859	Fitm2	fat storage-inducing tran	79.5093155	97.3495636	275.311951
80913	Pum2	pumilio 2 (Drosophila)	83.997345	110.64225	275.28894
103199	Fig4	FIG4 homolog (S. cerevi	85.3122406	121.058823	275.039154
66356	2310008H09Rik	RIKEN cDNA 2310008H	114.242859	112.670952	274.789368
214239	A430105I19Rik	RIKEN cDNA A430105I	50	50	274.725281
15561	Htr3a	5-hydroxytryptamine (se	50	50	274.498505
74174	Gtsf1	gametocyte specific facto	50	50	274.358826
27366	Txnl4a	thioredoxin-like 4A	50	50	274.033447
98366	Smap1	stromal membrane-assoc	50	52.8425407	273.870789
270685	Mthfd1l	methylenetetrahydrofolat	50	50	273.719604
332397	Nanos1	nanos homolog 1 (Droso	50	50	273.591431
209630	Frmd4a	FERM domain containin	78.8217773	106.552299	271.09201
271981	Tbck	TBC1 domain containing	50	50	269.898987
66583	Exosc1	exosome component 1	50	50	267.545837
12578	Cdkn2a	cyclin-dependent kinase	50	50	267.383148
18452	P4ha2	procollagen-proline, 2-ox	50	50	266.778412
56220	Zfp386	zinc finger protein 386 (I	50	60.4579697	265.708649
71860	Wdr16	WD repeat domain 16	50	50	264.918213
228071	Sestd1	SEC14 and spectrin dom	50	50	264.656952
234912	9230110C19Rik	RIKEN cDNA 9230110C	50	66.002243	264.150818
72569	Bbs5	Bardet-Biedl syndrome 5	50	55.0792084	264.011169
77532	Jrkl	jerky homolog-like (mou	50	50	263.912537
69706	Lrr1	leucine rich repeat protei	50	50	263.738373
17005	Ltk	leukocyte tyrosine kinase	50	50	263.610199
67883	Uxs1	UDP-glucuronate decarb	50	50	263.511597
28015	Polr2m	polymerase (RNA) II (D	67.8781357	84.6411514	263.186218
66209	1110054O05Rik	RIKEN cDNA 1110054O	97.775116	130.748215	263.058044
101351	A130022J15Rik	RIKEN cDNA A130022J	50	50	260.907013
211666	Mgst2	microsomal glutathione S	50	70.4105072	260.698334
20908	Stx3	syntaxin 3	104.230728	113.508469	260.418976
171170	Mbnl3	muscleblind-like 3 (Dros	50	50	260.128113
19099	Mapk8ip1	mitogen-activated protein	50	50	259.976929
210992	Lpcat1	lysophosphatidylcholine	50	50	259.640045
68190	5330426P16Rik	RIKEN cDNA 5330426P	50	50	259.621979
12981	Csf2	colony stimulating factor	50	50	258.861145
67291	Ccdc137	coiled-coil domain conta	75.4743652	96.5346832	258.657379
67180	Yipf5	Yip1 domain family, me	81.5117493	86.7901077	256.826782
64297	Gprc5b	G protein-coupled recept	50	50	255.715927
68925	Rpap1	RNA polymerase II asso	76.8542023	114.787369	255.617325
20729	Spin1	spindlin 1	50	50	255.507233
237877	Atad5	ATPase family, AAA do	50	50	254.698746
73673	2410076I21Rik	RIKEN cDNA 2410076I	97.9493866	110.246124	254.158112
54607	Socs6	suppressor of cytokine si	50	50	254.094025
14349	Fv1	Friend virus susceptibilit	50	50	253.91983
70408	Polr3f	polymerase (RNA) III (D	104.021614	124.662109	253.001236
57080	Gtf2ird1	general transcription fact	50	50	252.169754
16469	Jrk	jerky	50	72.2185135	252.123749
243312	Elfn1	leucine rich repeat and fi	50	73.22155	251.972565
73067	Tmem192	transmembrane protein 1	65.0091553	83.9366226	251.379333
230500	Efcab7	EF-hand calcium binding	50	50	250.275055
240334	Pcyox1l	prenylcysteine oxidase 1	50	50	248.501968
245522	Zc4h2	zinc finger, C4H2 domai	50	50	248.275192
320213	Senp5	SUMO/sentrin specific p	50	57.3611488	248.101013
74238	Mterfd3	MTERF domain containi	50	59.367218	247.670471
53892	Ppm1d	protein phosphatase 1D r	50	50	247.322113

67553	Gstcd	glutathione S-transferase	50	50	246.978668
15408	Hoxb13	homeobox B13	50	50	245.397827
65112	Pmepa1	prostate transmembrane	50	50	245.159546
116905	Dph1	DPH1 homolog (S. cerev	90.2184982	109.100204	244.328064
407821	Znrf3	zinc and ring finger 3	53.2734146	50	243.79892
268469	Zfp652	zinc finger protein 652	50	74.5782623	243.76442
240672	Dusp5	dual specificity phosphat	50	50	243.333893
67573	Loxl4	lysyl oxidase-like 4	103.742798	90.9578705	242.293701
15425	Hoxc6	homeobox C6	50	50	241.194351
633640	Gm7120	predicted gene 7120	50	50	241.182831
235973	A630095E13Rik	RIKEN cDNA A630095	50	50	239.915878
74430	4930452B06Rik	RIKEN cDNA 4930452	50	50	238.759018
72267	Lrrc8e	leucine rich repeat conta	50	53.391449	238.736008
116972	Fam57a	family with sequence sin	50	50	238.660431
69195	Tmem121	transmembrane protein 1	50	50	238.0672
53607	Snrpa	small nuclear ribonucleo	50	50	238.003113
19092	Prkg2	protein kinase, cGMP-de	50	50	236.974426
18693	Pick1	protein interacting with	50	58.3486214	236.445282
72155	Cenpn	centromere protein N	50	50	236.305603
66044	Dtd1	D-tyrosyl-tRNA deacylas	50	50	236.032837
79264	Krit1	KRIT1, ankyrin repeat c	50	64.0838852	235.6138
66371	Chmp4c	charged multivesicular b	50	63.5222473	234.940063
66311	Cenpw	centromere protein W	50	50	234.788879
15395	Hoxa10	homeobox A10	50	50	234.765869
233276	Tubgcp5	tubulin, gamma complex	71.1209869	105.627075	234.282745
109229	Fam118b	family with sequence sin	50	50	233.91629
237859	Ccdc55	coiled-coil domain conta	50	50	233.017426
230098	E130306D19Rik	RIKEN cDNA E130306	50	50	232.660843
58186	Rad18	RAD18 homolog (S. cere	50	50	232.108704
76916	4930455C21Rik	RIKEN cDNA 4930455	82.5478058	112.472893	231.858917
74998	Rab11fip2	RAB11 family interactin	50	65.2298126	231.817841
57764	Ntn4	netrin 4	50	50	231.480972
74365	Lonrf3	LON peptidase N-termin	50	51.508461	231.288712
26432	Plod2	procollagen lysine, 2-oxo	50	50	231.027435
50912	Exosc10	exosome component 10	50	50	230.853241
52592	Brms1l	breast cancer metastasis-	50	59.6529922	230.823669
105203	BC016423	cDNA sequence BC0164	63.4249573	85.0924454	229.242828
17245	Mdm1	transformed mouse 3T3	50	50	229.242828
14841	Gsg2	germ cell-specific gene 2	50	50	229.190247
71844	Nupl1	nucleoporin like 1	58.0703659	79.4052811	228.649612
66337	1700025K23Rik	RIKEN cDNA 1700025	50	57.6695557	227.155884
231830	Micall2	MICAL-like 2	50	69.232048	226.981689
71793	Ints12	integrator complex subun	59.524662	79.8792114	226.940613
72136	Chst14	carbohydrate (N-acetylga	50	56.3680153	226.388474
207596	Thsd4	thrombospondin, type I,	50	50	226.138702
171180	Syt12	synaptotagmin XII	74.3733521	96.0706558	225.673645
18973	Pole	polymerase (DNA directe	50	50	224.958832
69928	Apitd1	apoptosis-inducing, TAF	50	50	224.267014
74041	4632434I11Rik	RIKEN cDNA 4632434	50	50	223.348419
237436	Gas2l3	growth arrest-specific 2 l	50	65.8367233	223.046051
76167	Snrnp35	small nuclear ribonucleo	50	50	222.871887
269356	Slc4a11	solute carrier family 4, s	50	50	222.796295
68190	5330426P16Rik	RIKEN cDNA 5330426	50	50	222.755203
70546	Zdhhc2	zinc finger, DHHC doma	50	54.163887	221.522751
106565	Dlk2	delta-like 2 homolog (Dr	50	71.8025894	221.034698
223669	Zfp7	zinc finger protein 7	50	50	220.970612
13639	Efna4	ephrin A4	50	50	220.267288
20460	Stil	Scl/Tal1 interrupting locu	50	50	220.157196
66197	Cks2	CDC28 protein kinase re	50	50	219.831818
244238	Mrgpre	MAS-related GPR, mem	50	50	219.586975
57230	Sap30bp	SAP30 binding protein	53.9308548	76.0325928	219.174515

69137	2200002K05Rik	RIKEN cDNA 2200002K	50	50	219.098923
381066	Zfp948	zinc finger protein 948	50	50	218.901733
114679	Selm	selenoprotein M	50	56.9423904	217.552612
57808	Rpl35a	ribosomal protein L35A	50	50	216.384247
20055	Rps16	ribosomal protein S16	50	50	215.919189
56844	Tssc4	tumor-suppressing subch	50	50	215.919189
75216	4930534B04Rik	RIKEN cDNA 4930534B	50	65.5608521	214.634155
103765	Tmem17	transmembrane protein 1	50	50	214.448456
66184	Rps4y2	ribosomal protein S4, Y-	50	50	214.100098
13797	Emx2	empty spiracles homolog	50	50	213.541382
12572	Cdk7	cyclin-dependent kinase	64.6653824	72.8254318	213.390198
17954	Nap1l2	nucleosome assembly pro	50	50	213.239014
212391	Lcor	ligand dependent nuclear	50	65.2750778	212.913651
11486	Ada	adenosine deaminase	50	64.5252762	212.611298
171167	Fut10	fucosyltransferase 10	50	50	212.460114
240084	Cchcr1	coiled-coil alpha-helical	50	56.7216949	212.070648
69310	Pacrg	PARK2 co-regulated	50	68.8458252	211.972061
66101	Ppih	peptidyl prolyl isomerase	50	50	211.972061
108673	Ccdc86	coiled-coil domain conta	50	56.1501541	211.617111
69885	2610002D18Rik	RIKEN cDNA 2610002D	50	50	211.454437
17187	Max	Max protein	50	50	211.018951
76793	Snip1	Smad nuclear interacting	50	50	209.507156
77015	Mpped2	metallophosphoesterase d	96.7089539	76.8149338	209.355988
110253	Triobp	TRIO and F-actin bindin	50	50	209.204788
77622	Apex2	apurinic/apyrimidinic end	50	54.3294067	208.373291
20841	Zfp143	zinc finger protein 143	50	62.7073669	208.123505
20238	Atxn1	ataxin 1	83.8626938	98.8816986	208.047928
74111	Rbm19	RNA binding motif prote	50	51.2325897	207.809662
381259	Als2cr4	amyotrophic lateral scler	50	50	207.646973
72008	Zfyve19	zinc finger, FYVE doma	52.2373466	50	207.623962
69408	Dnajc17	DnaJ (Hsp40) homolog, s	50	67.9757767	207.582886
19014	Med1	mediator complex subuni	50	50	207.541809
67219	Med18	mediator of RNA polyme	50	50	207.15892
74187	Katnb1	katanin p80 (WD40-cont	50	50	206.879562
75284	Bcdin3d	BCDIN3 domain contain	50	60.1495552	205.885376
101831	C230052I12Rik	RIKEN cDNA C230052I	50	70.4331436	205.117981
56401	Lepre1	leprecan 1	50	59.8736877	204.577332
269060	Dagla	diacylglycerol lipase, alp	50	50	204.531326
66912	Bzw2	basic leucine zipper and	96.4998474	82.0833435	203.484558
264134	Ttc26	tetratricopeptide repeat d	50	50	203.408981
71785	Pdgfd	platelet-derived growth f	50	50	202.542969
100568458l71766	Raver1-fdx1l|Rave	Raver1-Fdx1l readthroug	50	50	202.066422
73754	Thap1	THAP domain containing	60.5559692	84.4756317	201.060745
66656	Eef1d	eukaryotic translation elo	50	50	200.682785
83672	Sytl3	synaptotagmin-like 3	59.9682312	84.2223969	200.595703
97863	C78339	expressed sequence C78	50	50	200.403427
230674	Kdm4a	lysine (K)-specific deme	50	63.9735413	200.217743
52864	Slx4	SLX4 structure-specific e	50	50	199.700104
20289	Scx	scleraxis	50	50	199.624527
78611	Btbd19	BTB (POZ) domain cont	50	50	198.281967
546336	Prrg1	proline rich Gla (G-carbo	50	50	197.968109
245631	Mum1l1	melanoma associated ant	50	50	197.968109
78755	Fam122b	family with sequence sin	51.7541656	69.0438843	197.85144
59004	Pias4	protein inhibitor of activ	50	70.8518982	197.752838
15275	Hk1	hexokinase 1	50	50	197.06102
78796	Zcchc4	zinc finger, CCHC doma	50	50	196.724152
57265	Fzd2	frizzled homolog 2 (Dros	50	50	196.660065
76303	Osbp	oxysterol binding protein	50	50	196.520386
75747	Sesn3	sestrin 3	50	72.2185135	196.305115
70892	Ttll7	tubulin tyrosine ligase-lik	50	50	195.881149
17454	Mov10	Moloney leukemia virus	67.1462402	80.5342255	195.70697

75668	Rasl10a	RAS-like, family 10, me	50	50	195.189331
386454	Rnf39	ring finger protein 39	50	50	195.038147
407793	BC039966	cDNA sequence BC0399	50	50	194.921478
101148	B630005N14Rik	RIKEN cDNA B630005I	50	50	194.57312
18483	Palm	paralemmin	50	62.7399025	194.561615
66768	Pacrgl	PARK2 co-regulated-like	50	50	194.19516
54651	Usp27x	ubiquitin specific peptida	95.5984344	91.3638916	194.043976
266744	Lgsn	lengsin, lens protein with	50	67.2033386	192.951202
24083	Gm16515	predicted gene, Gm1651	50	50	192.712921
24017	Rnf13	ring finger protein 13	50	71.0499573	192.195297
71310	Tbc1d9	TBC1 domain family, me	50	50	192.119705
332579	Card9	caspase recruitment dom	50	50	191.782837
108950	1700049L16Rik	hematological and neuro	50	61.4836349	190.271027
216019	Hkdc1	hexokinase domain conta	50	50	189.794479
18971	Pold1	polymerase (DNA directe	50	50	188.527527
58175	Rgs20	regulator of G-protein si	50	50	188.410843
208820	Gm11818	predicted gene 11818	50	70.764183	188.300751
237246	BC022960	cDNA sequence BC0229	50	50	188.096985
83671	Sytl2	synaptotagmin-like 2	50	60.3476181	187.696014
270118	Maml2	mastermind like 2 (Droso	50	62.9606018	187.673019
72543	Fam125b	family with sequence sin	50	70.6212921	187.673019
407785	Ndufs6	NADH dehydrogenase (u	79.4744644	70.465683	187.544846
67694	Ift74	intraflagellar transport 74	50	50	187.370651
67774	Loh12cr1	loss of heterozygosity, 12	78.6475143	80.2102585	186.864532
68423	Ankrd13d	ankyrin repeat domain 13	50	50	186.678833
19012	Ppap2a	phosphatidic acid phosph	50	50	186.550659
226182	Taf5	TAF5 RNA polymerase	63.4598083	91.7727432	186.539154
193452	Zfp184	zinc finger protein 184 (I	50	50	186.376465
18599	Padi1	peptidyl arginine deimina	50	50	185.835846
330836	Slc7a6	solute carrier family 7 (c	50	50	184.853165
214616	Spata5l1	spermatogenesis associat	50	50	184.84166
66067	Gtpbp8	GTP-binding protein 8 (p	50	50	184.84166
99512	Wdr47	WD repeat domain 47	50	62.7724457	183.650299
69727	Usp46	ubiquitin specific peptida	50	50	183.586197
14201	Fhl3	four and a half LIM dom	50	50.9241829	183.283844
384864	Gm1943	WD repeat domain 70 ps	50	50	183.045563
11490	Adam15	a disintegrin and metallo	50	60.6786613	182.452332
26448	Stk30	serine/threonine kinase 3	50	50	181.877197
71131	Zfp689	zinc finger protein 689	50	72.0204544	181.423645
69590	Gpx8	glutathione peroxidase 8	50	59.6529922	181.260971
13394	Dlx4	distal-less homeobox 4	50	50	181.231384
67096	Mmachc	methylmalonic aciduria c	59.041481	64.3045807	180.040024
57753	Noc3l	nucleolar complex associ	69.8805618	82.1611481	179.801743
100042856|74558|	Gm4070|Gvin1|Gn	predicted gene 4070|GTP	50	50	179.121429
665162	Gm15754	predicted gene 15754	50	50	179.086929
68052	Rps13	ribosomal protein S13	50	56.280304	178.354034
67980	Gnpda2	glucosamine-6-phosphate	50	50	177.726288
67574	Alg13	asparagine-linked glycos	62.4189911	82.5247345	177.650711
243979	Mrgprb2	MAS-related GPR, mem	50	50	176.860291
70239	Gtf3c5	general transcription fact	50	50	176.610519
60507	Qtrt1	queuine tRNA-ribosyltra	50	50	176.296661
227099	Pms1	postmeiotic segregation i	50	50.9567184	175.930206
240726	Slco5a1	solute carrier organic ani	50	50	175.174301
12836	Col7a1	collagen, type VII, alpha	50	50	174.697754
56176	Pigp	phosphatidylinositol glyc	50	50	174.535065
16201	Ilf3	interleukin enhancer binc	50	50	174.244202
100039596	Tcf24	transcription factor 24	50	50	174.16861
100038398	Gm10567	predicted gene 10567	50	50	173.692078
268880	AI480653	expressed sequence AI48	50	50	173.442291
675363	Gm9640	predicted gene 9640	50	64.6030884	173.279602
170762	Nup155	nucleoporin 155	50	54.3746796	172.860565

22351	Vill	villin-like	50	50	171.605118
67169	Nradd	neurotrophin receptor ass	50	50	170.988892
381677	Vgf	VGF nerve growth factor	50	70.8193588	170.901794
20588	Smarcc1	SWI/SNF related, matrix	50	50	170.686523
208836	Fanci	Fanconi anemia, complem	50	50	170.209976
78832	2700078E11Rik	RIKEN cDNA 2700078E	50.0305634	61.5388107	169.623322
64929	Scel	sciellin	50	50	169.355469
67220	Plekho1	pleckstrin homology dom	50	71.4913483	169.087616
75291	Zbtb3	zinc finger and BTB dom	50	50	168.803345
66590	Farsa	phenylalanyl-tRNA synth	50	70.0695572	168.79184
24069	Sufu	suppressor of fused homo	50	50	167.408203
16854	Lgals3	lectin, galactose binding,	50	50	167.344116
14823	Grm8	glutamate receptor, meta	50	50	167.291534
399558	Flrt2	fibronectin leucine rich t	50	50	167.041748
432628	Mfsd2b	major facilitator superfan	50	50	165.396835
78619	Zfp449	zinc finger protein 449	50	50	164.304062
56376	Pdlim5	PDZ and LIM domain 5	50	50	163.548157
83922	Tsga14	testis specific gene A14	50	65.0543823	162.769257
70325	Pigw	phosphatidylinositol glyc	50	70.3779678	162.693665
56455	Dynll1	dynein light chain LC8-t	50	54.9363213	162.682159
17309	Mgat3	mannoside acetylglucosa	50	50	161.763565
51944	D2Ertd750e	DNA segment, Chr 2, ER	50	50	161.548309
74307	1700092M07Rik	RIKEN cDNA 1700092M	58.0006599	57.3385162	161.472717
320642	A630066F11Rik	RIKEN cDNA A630066	58.414135	66.5313492	161.245941
399603	Fam84b	family with sequence sim	50	50	161.158844
70422	Ints2	integrator complex subun	50	50	160.769379
270685	Mthfd1l	methylenetetrahydrofolat	50	50	160.519608
71924	Tube1	epsilon-tubulin 1	50	50	159.967468
171567	Nme7	non-metastatic cells 7, pr	50	63.6552277	159.612518
68229	AI846148	expressed sequence AI84	50	50	159.415344
12556	Cdh16	cadherin 16	50	50	158.973297
230767	Iqcc	IQ motif containing C	50	50	158.315979
69955	Fars2	phenylalanine-tRNA syn	50	50	158.130295
17865	Mybl2	myeloblastosis oncogene	50	50	158.043213
72415	Sgol1	shugoshin-like 1 (S. pom	50	50	157.839447
215798	Gpr126	G protein-coupled recept	50	50	157.665253
192897	Itgb4	integrin beta 4	50	50	157.310303
140571	Plxnb3	plexin B3	50	64.5153809	156.932343
68531	1110020A21Rik	RIKEN cDNA 1110020A	50	50	156.81076
69269	Scnm1	sodium channel modifier	50	72.1307983	156.735153
16878	Lif	leukemia inhibitory facto	50	50	156.40979
21898	Tlr4	toll-like receptor 4	50	50	156.141937
192287	Slc25a36	solute carrier family 25,	50	53.1834869	156.118927
243659	Styk1	serine/threonine/tyrosine	50	50	156.05484
21983	Tpbg	trophoblast glycoprotein	50	50	156.03183
22673	Zfp185	zinc finger protein 185	50	57.371048	155.729477
17688	Msh6	mutS homolog 6 (E. coli)	50	73.3092651	155.717987
212679	Mars2	methionine-tRNA synthe	50	50	154.107574
22038	Plscr1	phospholipid scramblase	50	50	153.95639
194744	Slc25a43	solute carrier family 25,	50	50.5931396	153.816711
58230	Rnf8	ring finger protein 8	50	50	153.654022
18510	Pax8	paired box gene 8	50	50	153.566925
14420	Galc	galactosylceramidase	50	50	153.427246
66658	Ccdc51	coiled-coil domain conta	50	72.7150803	152.962204
233902	Fbxl19	F-box and leucine-rich re	50	73.2441864	152.950714
77065	Ints7	integrator complex subun	50	50	152.247391
67371	Gtf3c6	general transcription fact	50	61.9349289	151.945023
217216	BC030867	cDNA sequence BC0308	50	50	151.880936
70551	Tmtc4	transmembrane and tetra	50	50	151.619659
12021	Bard1	BRCA1 associated RING	50	50	151.521057
268980	Strn	striatin, calmodulin bindi	50	50	151.47998

67304	3110070M22Rik	RIKEN cDNA 3110070M	50	60.9545326	151.427399
100043040	1110002L01Rik	RIKEN cDNA 1110002L	50	50	151.264709
80707	Wwox	WW domain-containing	52.8852844	72.2185135	151.177612
64051	Sv2a	synaptic vesicle glycopro	50	50	150.549881
102747	Lrrc49	leucine rich repeat contai	50	50	150.334625
74125	Armc8	armadillo repeat containi	50	50	150.259033
217430	Pqlc3	PQ loop repeat containin	50	52.0149307	149.956665
69785	Ceacam13	carcinoembryonic antige	50	50	149.445618
55984	Camkk1	calcium/calmodulin-depe	50	50	149.20076
66236	1500011B03Rik	RIKEN cDNA 1500011B	50	50	149.113678
18227	Nr4a2	nuclear receptor subfami	50	65.9470673	147.306061
71354	Wdr31	WD repeat domain 31	50	50	147.003708
14853	Gspt2	G1 to S phase transition 2	50	62.3536911	147.003708
76781	Mettl4	methyltransferase like 4	50	50	146.538666
212276	Zfp748	zinc finger protein 748	50	59.4874687	145.933929
57258	Xpo4	exportin 4	50	50	145.846848
94092	Trim16	tripartite motif-containing	50	50	145.457397
94216	Col4a6	collagen, type IV, alpha 6	50	50	145.143524
12386	Ctnna2	catenin (cadherin associa	50	50	145.067932
56190	Rbm38	RNA binding motif prote	50	50	145.067932
71592	Pogk	pogo transposable eleme	53.7217407	50	144.21344
66067	Gtpbp8	GTP-binding protein 8 (p	50	50	144.178925
72469	Plcd3	phospholipase C, delta 3	50	50	144.050751
246154	Vasn	vasorin	50	50	143.487122
213027	Evi5l	ecotropic viral integratio	50	50	143.44603
71831	1700007B14Rik	RIKEN cDNA 1700007B	50	50	143.434525
226075	Glis3	GLIS family zinc finger	50	50	142.916901
194952	Jmjd4	jumonji domain containin	50	50	142.89389
74420	4933406P04Rik	RIKEN cDNA 4933406P	70.6077118	64.580452	141.760025
100043899	R3hdml	R3H domain containing-	50	50	141.33606
100040608	Fancf	Fanconi anemia, comple	53.2686615	56.3354759	141.271988
214639	4930486L24Rik	RIKEN cDNA 4930486L	50	50	140.795441
381534	Ube2u	ubiquitin-conjugating en	50	50	140.754349
235584	Dusp7	dual specificity phosphat	50	56.1374168	140.568665
74254	Gpn1	GPN-loop GTPase 1	50	63.5547791	140.330383
66993	Smarcd3	SWI/SNF related, matrix	50	51.6838837	140.202209
68916	Cdkal1	CDK5 regulatory subunit	50	50	140.190704
71988	Esco2	establishment of cohesio	50	50	139.888336
74255	Smu1	smu-1 suppressor of mec	50	56.6113472	139.475891
110784	Nr3c2	nuclear receptor subfami	50	50	139.411804
117171	1110038F14Rik	RIKEN cDNA 1110038F	50	50	139.272125
17756	Mtap2	microtubule-associated p	50	50	139.26062
22351	Vill	villin-like	50	50	139.173523
71436	Flrt3	fibronectin leucine rich t	50	50	138.807068
228356	1110051M20Rik	RIKEN cDNA 1110051M	50	50	138.772568
232879	Zbtb45	zinc finger and BTB dom	50	50	138.731476
209737	Kif15	kinesin family member 1	50	50	138.254944
223739	5031439G07Rik	RIKEN cDNA 5031439G	50	50	138.10376
18968	Pola1	polymerase (DNA directe	50	50	137.88356
237336	Tbpl1	TATA box binding prote	50	52.7647285	137.400436
106628	Trip10	thyroid hormone receptor	50	52.9302521	136.882812
56224	Tspan5	tetraspanin 5	50	50	136.871307
14862	Gstm1	glutathione S-transferase	50	50	135.918213
73261	1700037C18Rik	RIKEN cDNA 1700037C	50	50	135.725952
233552	Gdpd5	glycerophosphodiester ph	50	50	135.336487
66520	2610001J05Rik	RIKEN cDNA 2610001J	50	65.6613007	135.226395
22685	Zfp239	zinc finger protein 239	50	50	135.162292
71544	Arhgap42	Rho GTPase activating p	50	50	135.162292
229521	Syt11	synaptotagmin XI	50	50	134.924026
73451	Zfp763	zinc finger protein 763	50	54.2515984	134.621674
55979	Agpat1	1-acylglycerol-3-phospha	50	50	134.534576

13175	Dclk1	doublecortin-like kinase 1	50	50	134.220703
67071	Rps6ka6	ribosomal protein S6 kin	50	50	133.615982
68545	Ecscr	endothelial cell-specific c	50	57.9227905	133.551895
71532	9030418K01Rik	RIKEN cDNA 9030418K	50	54.8811493	132.988251
18378	Omp	olfactory marker protein	50	50.7034874	132.621811
26447	Poli	polymerase (DNA directe	50	50	132.459106
51801	Ramp1	receptor (calcitonin) acti	50	50	132.18634
77744	6720463M24Rik	RIKEN cDNA 6720463M	50	65.5509491	131.842896
73340|504193	Nptxr|Npcd	neuronal pentraxin recep	50	50	131.767303
68736	1110034B05Rik	RIKEN cDNA 1110034B	50	50	131.709793
68980	Wdr53	WD repeat domain 53	50	50	131.627625
108812	Als2cr12	amyotrophic lateral scler	50	50	131.616119
68617	1110012J17Rik	RIKEN cDNA 1110012J	50	50	131.593109
230996	9430015G10Rik	RIKEN cDNA 9430015G	50	50	131.506027
68519	Eml1	echinoderm microtubule	50	57.3059731	130.988388
67463	Poc5	POC5 centriolar protein	50	50	130.523346
100503044	A730020M07Rik	RIKEN cDNA A730020	50	50	130.360657
76813	Armc6	armadillo repeat containi	54.4140358	57.260704	129.884125
66548	Adamtsl5	ADAMTS-like 5	50	50	129.820023
13713	Elk3	ELK3, member of ETS o	50	50	129.593262
224111	Ubxn7	UBX domain protein 7	50	50	129.180801
14687	Gnaz	guanine nucleotide bindi	50	50	129.128204
71020	Spats1	spermatogenesis associat	50	61.6590576	129.116714
72661	Serp2	stress-associated endopla	50	50	128.96553
17930	Myom2	myomesin 2	50	50	128.58757
70928	Trim69	tripartite motif-containin	50	50	128.360809
100113398	Adat3	adenosine deaminase, tR	50	50	128.360809
67216	Mboat2	membrane bound O-acyl	50	50	128.122528
19726	Rfx3	regulatory factor X, 3 (in	50	50	127.808662
237339	L3mbtl3	l(3)mbt-like 3 (Drosophi	50	56.0270691	127.494797
16578	Kif9	kinesin family member 9	50	62.0778198	126.965668
13640	Efna5	ephrin A5	50	50	126.954163
69942	Rnf113a1	ring finger protein 113A	50	60.600853	126.878571
109168	Atl3	atlastin GTPase 3	50	50	126.878571
18452	P4ha2	procollagen-proline, 2-ox	50	50	126.867065
72371	2210408I21Rik	RIKEN cDNA 2210408I	50	50	126.825989
14221	Fjx1	four jointed box 1 (Droso	50	50	126.402023
71176	Fbxo24	F-box protein 24	50	56.666523	126.273849
100216533	Snord55	small nucleolar RNA, C/	50	50	126.262344
70835	Prss22	protease, serine, 22	50	50	126.11116
19653	Rbm4	RNA binding motif prote	50	50	125.808807
66101	Ppih	peptidyl prolyl isomerase	50	50	125.646118
210035	Tmem194	transmembrane protein 1	50	52.8199043	125.181076
21819	Tg	thyroglobulin	50	50	125.169571
77286	Nkrf	NF-kappaB repressing fa	50	50	125.006889
385658	Fam55c	family with sequence sin	50	50	124.739029
109294	Prex2	phosphatidylinositol-3,4,	50	50	124.628937
73420	1700054N08Rik	RIKEN cDNA 1700054N	50	50	124.349579
17196	Mbp	myelin basic protein	50	50	124.088295
70808	4632415L05Rik	RRS1 ribosome biogenes	50	50	123.884537
225182	Rbbp8	retinoblastoma binding p	50	50	123.861526
71934	Car13	carbonic anhydrase 13	50	50	123.570671
11982	Atp10a	ATPase, class V, type 10	50	50	123.105621
19725	Rfx2	regulatory factor X, 2 (in	50	50	122.85585
19058	Ppp3r1	protein phosphatase 3, re	50	50	122.693161
70080	2210010C17Rik	RIKEN cDNA 2210010C	50	50	122.663582
218397	Rasa1	RAS p21 protein activato	50	52.0474701	122.564987
24113	Vax2	ventral anterior homeobo	50	50	121.245438
226250	Afap1l2	actin filament associated	50	50	121.222435
20761	Sprr2g	small proline-rich protein	50	50	121.007172
12704	Cit	citron	50	50	120.844482

13527	Dtna	dystrobrevin alpha	50	50	120.757385
75871	Zfp821	zinc finger protein 821	50	50	120.681801
70645	Oip5	Opa interacting protein 5	50	50	120.303848
224014	Fgd4	FYVE, RhoGEF and PH	50	50	120.228256
76498	Paqr4	progestin and adipoQ rec	50	50	120.216751
209212	Osgin2	oxidative stress induced	50	59.2894058	119.850304
109294	Prex2	phosphatidylinositol-3,4,	50	50	119.838806
69234	Zfp688	zinc finger protein 688	50	53.5597992	118.943214
74051	Steap2	six transmembrane epithe	50	50	118.769035
74243	2210009G21Rik	RIKEN cDNA 2210009G	50	50	118.681938
242297	Fam110b	family with sequence sim	50	50	118.681938
21983	Tpbg	trophoblast glycoprotein	50	50	118.606354
74559	Elovl7	ELOVL family member	50	50	118.466675
16196	Il7	interleukin 7	50	50	118.391083
72119	Tpx2	TPX2, microtubule-assoc	50	50	118.391083
18393	Orc2	origin recognition compl	50	50	118.29248
22070	Tpt1	tumor protein, translation	50	50	118.141304
67803	Limd2	LIM domain containing 2	50	50	118.118301
12023	Barx2	BarH-like homeobox 2	50	50	117.815933
12064	Bdnf	brain derived neurotroph	50	50	117.618744
238799	Tnpo1	transportin 1	50	50	117.449486
68549	Sgol2	shugoshin-like 2 (S. pom	50	50	117.222717
234258	Neil3	nei like 3 (E. coli)	50	50	117.211212
192656	Ripk2	receptor (TNFRSF)-inter	50	50	117.037018
66296	Haus2	HAUS augmin-like comp	50	50	116.972939
18020	Nfatc2ip	nuclear factor of activate	50	52.4336853	115.967255
54216	Pcdh7	protocadherin 7	50	50	115.967255
50762	Fbxo6	F-box protein 6	50	50	115.653397
320150	Zdhhc17	zinc finger, DHHC doma	50	50	115.135757
20166	Rtkn	rhotekin	50	52.4336853	114.723297
22348	Slc32a1	solute carrier family 32 (	50	50	114.508026
18423	Otx1	orthodenticle homolog 1	50	50	114.420944
15331	Hmgn2	high mobility group nucl	50	50	114.333847
15245	Hhip	Hedgehog-interacting pro	55.6893158	50	114.281265
245469	Pdzd4	PDZ domain containing 4	50	50	113.717621
231503	Tmem150c	transmembrane protein 1	50	50	113.014297
208228	Mob3a	MOB kinase activator 3A	50	50	112.810532
73293	Ccdc103	coiled-coil domain conta	50	50	112.799026
23967	Osr1	odd-skipped related 1 (D	50	50	112.64785
270106	Rpl13	ribosomal protein L13	50	50	112.258392
12005	Axin1	axin 1	50	50	112.1828
381073	Npw	neuropeptide W	50	50	112.171295
68800	1110059M19Rik	RIKEN cDNA 1110059M	50	50	111.81636
75782	Lca5	Leber congenital amauro	50	50	111.177124
213389	Prdm9	PR domain containing 9	50	50	111.101532
19240	Tmsb10	thymosin, beta 10	50	50	110.863258
56468	Socs5	suppressor of cytokine si	50	50	110.560898
225467	Pggt1b	protein geranylgeranyltra	50	50	110.398209
269224	Pask	PAS domain containing s	50	50	110.031761
73720	Cst6	cystatin E/M	50	50	109.834564
105522	Ankrd28	ankyrin repeat domain 28	50	52.6770172	109.630806
26465	Zfp146	zinc finger protein 146	50	50	109.630806
99010	Lpcat4	lysophosphatidylcholine	50	50	109.555214
79456	Recql4	RecQ protein-like 4	50	50	109.252853
18181	Nrf1	nuclear respiratory factor	50	50	109.252853
98488	Gtf3c3	general transcription fact	50	50	108.950485
68876	Xrcc6bp1	XRCC6 binding protein	50	50	108.851891
69312	Ppp1r42	protein phosphatase 1, re	50	50	108.851891
12316	Aspm	asp (abnormal spindle)-li	50	50	108.590614
382090	4922501C03Rik	RIKEN cDNA 4922501C	50	50	108.450935
73467	1700066M21Rik	RIKEN cDNA 1700066M	50	50	108.398354

	73681	Trmt11	tRNA methyltransferase	53.7217407	50	108.13707
	113853	Vmn1r53	vomeronasal 1 receptor 5	50	50	107.933304
	170763	Zfp87	zinc finger protein 87	50	50	107.706535
	22696	Zfp37	zinc finger protein 37	50	50	107.381165
	68659	Fam198b	family with sequence sin	50	50	107.317078
	52665	Echdc1	enoyl Coenzyme A hydra	50	50	106.84053
	16420	Itgb6	integrin beta 6	50	50	106.613754
	212679	Mars2	methionine-tRNA synthe	50	50	106.375488
	266690	Cyb5r4	cytochrome b5 reductase	50	50	106.322899
	71710	Lrrcc1	leucine rich repeat and c	50	50	106.160217
	76820	Fam49a	family with sequence sin	50	50	105.770767
	13175	Dclk1	doublecortin-like kinase	50	50	105.497978
	100900	Hscb	HscB iron-sulfur cluster	50	50	105.433884
	12566	Cdk2	cyclin-dependent kinase	50	50	105.282715
	20732	Spint1	serine protease inhibitor,	50	50	105.055939
	76507	Abp1	amiloride binding protein	50	50	105.055939
	17279	Melk	maternal embryonic leuc	50	50	104.968842
	101631	Pwwp2b	PWWP domain containin	50	50	104.852173
	22367	Vrk1	vaccinia related kinase 1	50	50	104.765076
	19279	Ptprr	protein tyrosine phospha	50	50	104.590897
	100217451	Snora7a	small nucleolar RNA, H/	50	50	104.515305
	65102	Nif3l1	Ngg1 interacting factor 3	50	50	104.428207
	269437	Plch1	phospholipase C, eta 1	50	50	104.201431
	30050	Fbxw2	F-box and WD-40 domai	50	50	104.178429
	268465	Eme1	essential meiotic endonu	50	50	104.027252
	74007	Btbd11	BTB (POZ) domain cont	50	50	103.899078
	225289	AW554918	expressed sequence AW5	50	50	103.445534
	66660	Sltm	SAFB-like, transcription	50	50	103.381447
	225998	Rorb	RAR-related orphan rece	50	50	103.218765
	277328	Trpa1	transient receptor potenti	50	50	103.143173
	319859	E030011O05Rik	RIKEN cDNA E030011O	50	50	102.980484
	230967	BC046331	cDNA sequence BC0463	50	50	102.806297
	12669	Chrm1	cholinergic receptor, mus	50	50	102.666626
	78767	2610021K21Rik	RIKEN cDNA 2610021K	50	50	102.43985
	15427	Hoxc9	homeobox C9	50	50	102.43985
	209334	Gen1	Gen homolog 1, endonuc	50	50	102.428345
	218311	Zfp455	zinc finger protein 455	50	50	102.35276
	72022	Slc35f2	solute carrier family 35,	50	50	102.254166
	11537	Cfd	complement factor D (ad	50	50	102.147346
	19383	Raly	hnRNP-associated with l	50	50	101.736526
	76072	Rnf183	ring finger protein 183	50	50	101.725029
	387524	Znrf2	zinc and ring finger 2	50	50	101.49826
	667063	Gm8439	predicted gene 8439	50	50	100.615822
	71177	4933424B01Rik	RIKEN cDNA 4933424B	50	50	100.492577
	270097	Vat1l	vesicle amine transport p	50	50	100.42849
	74320	Wdr33	WD repeat domain 33	50	50	100.42849
	71361	Aifm2	apoptosis-inducing factor	50	50	100.265808
Pattern II -Down	71760	Agxt2l1	alanine-glyoxylate amino	1141.25317	1633.60291	50
	18171	Nr1i2	nuclear receptor subfami	529.233032	578.67334	50
	14120	Fbp2	fructose bisphosphatase 2	103.608139	101.559761	50
	18670	Abcb4	ATP-binding cassette, su	23244.5625	18359.5586	50
	246278	Cd207	CD207 antigen	652.272949	448.203827	50
	18383	Tnfrsf11b	tumor necrosis factor rec	114.93515	107.071503	50
	26876	Adh4	alcohol dehydrogenase 4	4548.24072	3390.19434	50
	18815	Plg	plasminogen	37147.4453	38324.5977	50
	100038347	Fam174b	family with sequence sin	178.564484	248.411713	50
	232714	Mgam	maltase-glucoamylase	737.938477	619.775146	50
	23923	Aadat	aminoadipate aminotrans	6195.4043	5038.36865	50
	73644	2210039B01Rik	RIKEN cDNA 2210039B	266.984894	242.338318	50
	14317	Ftcd	formiminotransferase cyc	18980.9883	19433.0449	50
	20493	Slc10a1	solute carrier family 10 (	144.832138	145.485336	50

18762	Prkcz	protein kinase C, zeta	311.038269	322.52594	50
11699	Ambp	alpha 1 microglobulin/bi	12280.5703	12746.127	50
69861	2010003K11Rik	RIKEN cDNA 2010003I	1053.59302	1044.22729	50
216166	Plk5	polo-like kinase 5 (Droso	128.259842	174.55072	50
15360	Hmgcs2	3-hydroxy-3-methylgluta	43320.8672	31370.6426	50
67260	Lass4	LAG1 homolog, ceramid	130.884857	189.122314	50
104816	Aspg	asparaginase homolog (S	504.999573	700.176392	50
74374	Clec16a	C-type lectin domain fan	160.75174	209.182999	50
272538	Tmco7	transmembrane and coile	154.465652	212.666046	50
170757	Eltd1	EGF, latrophilin seven tr	692.18689	1010.27686	50
22413	Wnt2	wingless-related MMTV	309.214874	326.008972	50
50500	Ttpa	tocopherol (alpha) transf	1877.34912	1559.39111	50
22035	Tnfsf10	tumor necrosis factor (lig	118.730896	121.367233	50
27276	Plekhb1	pleckstrin homology dom	4014.43701	2767.56982	50
64381	Ms4a8a	membrane-spanning 4-do	157.334625	213.107422	50
67477	Abhd15	abhydrolase domain cont	494.912994	376.271088	50
69865	A1cf	APOBEC1 complementa	1454.63281	1512.0603	50
11826	Aqp1	aquaporin 1	1022.30841	1308.35645	50
108000	Cenpf	centromere protein F	135.582001	134.78299	50
117147	Acsm1	acyl-CoA synthetase mec	1295.39563	1872.73413	50
234353	Psd3	pleckstrin and Sec7 doma	130.297119	106.542397	50
108114	Slc22a7	solute carrier family 22 (	248.261261	278.204254	50
14198	Fhit	fragile histidine triad gen	495.017578	359.518005	50
14618	Gjb1	gap junction protein, beta	4024.8501	4184.51758	50
75847	Ispd	isoprenoid synthase dom	284.662964	229.419128	50
64075	Smoc1	SPARC related modular	4029.05786	5223.9248	50
26459	Slc27a5	solute carrier family 27 (	4516.96387	4915.38867	50
78057	4930583I09Rik	RIKEN cDNA 4930583I	318.146576	262.752716	50
66825	Rnf186	ring finger protein 186	750.322144	757.690308	50
230558	C8a	complement component	1859.17371	2028.58228	50
330166	Miat	myocardial infarction ass	276.3396	342.885162	50
15112	Hao1	hydroxyacid oxidase 1, li	4376.65137	5871.02246	50
11818	Apoh	apolipoprotein H	11227.9082	15509.0996	50
76654	Upp2	uridine phosphorylase 2	778.927979	988.706665	50
14121	Fbp1	fructose bisphosphatase 1	14388.4795	11550.7891	50
76559	Atg2b	ATG2 autophagy related	113.206802	134.263794	50
53872	Caprin1	cell cycle associated prot	141.823746	146.387924	50
234199	Fgl1	fibrinogen-like protein 1	15284.9863	21015.8398	50
18979	Pon1	paraoxonase 1	49672.668	39213.1484	50
244416	Ppp1r3b	protein phosphatase 1, re	2863.70264	2437.42017	50
217721	Mfsd7c	major facilitator superfan	162.654373	216.028824	50
14547	Gdap2	ganglioside-induced diffe	213.298004	206.4823	50
16912	Psmb9	proteasome (prosome, ma	780.038574	843.784363	50
218865	Chdh	choline dehydrogenase	1662.44165	2009.33936	50
22262	Uox	urate oxidase	12504.1201	12981.9727	50
12165	Gdf2	growth differentiation fac	531.430298	717.059631	50
54140	Avpr1a	arginine vasopressin rece	683.907837	559.560486	50
320440	9530091C08Rik	RIKEN cDNA 95300910	269.052277	332.380859	50
12611	Cebpg	CCAAT/enhancer bindin	126.287521	164.585449	50
55985	Cxcl13	chemokine (C-X-C motif	153.778107	136.886688	50
234878	BC021891	cDNA sequence BC0218	330.265686	295.531738	50
74637	Shpk	sedoheptulokinase	306.998596	272.572266	50
27413	Abcb11	ATP-binding cassette, su	6217.62109	5074.75977	50
212965	BC024386	cDNA sequence BC0234	9567.01367	8524.57422	50
18551	Pcsk4	proprotein convertase sul	179.774811	213.834595	50
79235	Lrat	lecithin-retinol acyltransf	345.901733	366.945251	50
16918	Mycl1	v-myc myelocytomatosis	372.625549	356.32074	50
67473	Slc47a1	solute carrier family 47,	5141.84766	4589.18652	50
15160	Serpind1	serine (or cysteine) pepti	3774.02539	4364.03223	50
22139	Ttr	transthyretin	43707.8125	56918.5312	50
71776	Tha1	threonine aldolase 1	201.971008	219.466583	50

13106	Cyp2e1	cytochrome P450, family	33193.5547	33997.9375	50
243537	Uroc1	urocanase domain contai	10726.1807	9933.13672	50
239037	Lrit1	leucine-rich repeat, immu	796.134033	533.108093	50
72107	Dscc1	defective in sister chrom	395.583771	293.295044	50
71775	1300017J02Rik	RIKEN cDNA 1300017J	11207.3037	11010.0908	50
71902	Cand1	cullin associated and ned	108.683914	106.035927	50
15109	Hal	histidine ammonia lyase	5785.76709	6030.59814	50
75957	Mir17hg	MIR17 host gene 1 (non-	129.818695	132.676483	50
13082	Cyp26a1	cytochrome P450, family	369.69165	377.394348	50
103655	Sec14l4	SEC14-like 4 (S. cerevis	8139.44189	10322.5332	50
12352	Car5a	carbonic anhydrase 5a, m	3386.45801	4703.62109	50
110446	Acat1	acetyl-Coenzyme A acety	494.987427	403.420929	50
15139	Hc	hemolytic complement	11510.5723	16217.3984	50
71724	Aox3	aldehyde oxidase 3	3931.23828	5579.85059	50
16790	Anpep	alanyl (membrane) amino	163.650818	154.291962	50
75462	1700001C19Rik	RIKEN cDNA 1700001C	227.345093	184.723938	50
71756	Cpn2	carboxypeptidase N, poly	4499.86523	5024.61621	50
16995	Ltb4r1	leukotriene B4 receptor 1	121.873947	165.214996	50
99586	Dpyd	dihydropyrimidine dehyd	6072.52246	4569.70703	50
20672	Sox18	SRY-box containing gene	109.620171	100.280853	50
11607	Agtr1a	angiotensin II receptor, t	639.596191	929.134277	50
237758	Zfp454	zinc finger protein 454	121.664825	129.446686	50
212980	Slc45a3	solute carrier family 45,	361.582123	530.189514	50
104158	Ces1d	carboxylesterase 1D	15496.9414	10711.0664	50
14164	Fgf1	fibroblast growth factor 1	479.69043	364.322388	50
233058	Zfp420	zinc finger protein 420	119.557846	134.043091	50
103149	Upb1	ureidopropionase, beta	6929.81934	6980.72559	50
212892	Rsph4a	radial spoke head 4 homo	144.662628	101.196182	50
17175	Masp2	mannan-binding lectin se	960.416992	1321.03809	50
13123	Cyp7b1	cytochrome P450, family	614.710083	755.849792	50
268756	Gulo	gulonolactone (L-) oxida	4376.4502	5662.58887	50
11812	Apoc1	apolipoprotein C-I	44939.2422	59389.4258	50
16424	Itih1	inter-alpha trypsin inhibi	16600.584	18281.1211	50
28253	Slco1b2	solute carrier organic ani	37855.9453	33193.5	50
26930	Ppnr	per-pentamer repeat gene	725.819336	815.934204	50
30924	Angptl3	angiopoietin-like 3	4487.88086	5351.78809	50
19647	Rbbp6	retinoblastoma binding p	133.918594	126.768616	50
66107	1100001G20Rik	RIKEN cDNA 1100001G	13160.2061	12197.0547	50
13009	Csrp3	cysteine and glycine-rich	880.8078	1075.52222	50
14061	F2	coagulation factor II	24353.9277	31468.9766	50
54326	Elovl2	elongation of very long c	5408.18164	4932.35986	50
15233	Hgd	homogentisate 1, 2-dioxy	4138.99463	5917.04736	50
20872	Stk16	serine/threonine kinase 1	226.966476	176.97554	50
217316	Slc16a5	solute carrier family 16 (	994.653076	1444.38586	50
20304	Ccl5	chemokine (C-C motif) l	108.305283	150.377426	50
16596	Klf1	Kruppel-like factor 1 (er	141.863342	121.367233	50
52685	Cd300lg	CD300 antigen like fami	172.352844	186.062256	50
16011	Igfbp5	insulin-like growth facto	308.757019	362.095612	50
236920	Stard8	START domain containi	607.989868	718.682251	50
230163	Aldob	aldolase B, fructose-bisp	16058.3555	21430.3203	50
18573	Pde1a	phosphodiesterase 1A, ca	286.551331	193.309875	50
329251	Ppp1r12b	protein phosphatase 1, re	108.40509	161.939911	50
243168	Hsd17b13	hydroxysteroid (17-beta)	179.839752	215.421906	50
14130	Fcgr2b	Fc receptor, IgG, low aff	463.492004	682.073608	50
76263	Gstk1	glutathione S-transferase	7946.54053	5457.95166	50
18703	Pigr	polymeric immunoglobul	22524.6816	19294.6367	50
237320	Aldh8a1	aldehyde dehydrogenase	3541.88672	3345.83447	50
74519	Cyp2j9	cytochrome P450, family	493.284424	413.834656	50
330938	Dixdc1	DIX domain containing 1	591.308228	794.581909	50
11536	Gpr182	G protein-coupled recept	377.680756	520.691162	50
20743	Spnb3	spectrin beta 3	895.821289	1059.56836	50

226143	Cyp2c44	cytochrome P450, family	3632.66919	2482.27661	50
20317	Serpinf1	serine (or cysteine) pepti	19642.5957	18500.1953	50
210108	D130043K22Rik	RIKEN cDNA D130043	430.779907	588.041626	50
668253	Dleu2	deleted in lymphocytic le	335.122864	228.406189	50
76257	Slc38a3	solute carrier family 38,	2500.53467	2626.40527	50
56508	Rapgef4	Rap guanine nucleotide e	363.654236	419.612366	50
102141	Snx25	sorting nexin 25	505.57782	487.585327	50
11814	Apoc3	apolipoprotein C-III	18147.2363	24045.1055	50
72090	Entpd8	ectonucleoside triphosph	244.400574	220.888382	50
76654	Upp2	uridine phosphorylase 2	1723.55518	2082.40381	50
19130	Prox1	prospero-related homeob	302.470947	367.850708	50
218103	Slc17a2	solute carrier family 17 (	1605.58008	1156.63501	50
230678	Tmem125	transmembrane protein 1	174.942993	141.109619	50
17858	Mx2	myxovirus (influenza vir	277.789124	189.011963	50
66901	Proz	protein Z, vitamin K-dep	992.755127	1137.65234	50
76654	Upp2	uridine phosphorylase 2	1460.32007	1712.69556	50
19699	Reln	reelin	1631	1540.87244	50
57757	Pglyrp2	peptidoglycan recognitio	460.323608	636.485779	50
13982	Esr1	estrogen receptor 1 (alph	1560.07861	2126.44678	50
17761	Mtap7	microtubule-associated p	199.490143	222.926987	50
13195	Ddc	dopa decarboxylase	1013.37512	1228.68811	50
11536	Gpr182	G protein-coupled recept	296.294128	362.040436	50
19228	Pth1r	parathyroid hormone 1 re	128.812729	143.070404	50
70373	1700020O03Rik	RIKEN cDNA 1700020O	120.668365	140.051407	50
26458	Slc27a2	solute carrier family 27 (	40538.6953	30344.6699	50
18534	Pck1	phosphoenolpyruvate car	29267.418	40259.2969	50
170442	Bbox1	butyrobetaine (gamma),	6385.625	4260.0293	50
93841	Uchl4	ubiquitin carboxyl-termin	1487.69189	1577.74426	50
20678	Sox5	SRY-box containing gen	739.761902	864.758972	50
433855	AI506816	expressed sequence AI50	135.133682	100.215775	50
68910	Zfp467	zinc finger protein 467	271.577515	332.919861	50
20493	Slc10a1	solute carrier family 10 (	148.46312	132.280365	50
17289	Mertk	c-mer proto-oncogene ty	581.932983	779.711792	50
224860	Plcl2	phospholipase C-like 2	125.360764	136.941864	50
269423	3110057O12Rik	RIKEN cDNA 3110057O	197.13443	194.897186	50
72792	2810459M11Rik	RIKEN cDNA 2810459M	1282.34497	1052.58691	50
16427	Itih4	inter alpha-trypsin inhibi	3410.01489	4571.86035	50
625098	Slc38a6	solute carrier family 38,	226.448441	336.402924	50
66451	2610528J11Rik	RIKEN cDNA 2610528J	128.473724	147.270706	50
225884	BC021614	cDNA sequence BC0216	1090.12622	1614.26514	50
12359	Cat	catalase	174.455048	150.565582	50
22354	Vipr1	vasoactive intestinal pept	652.875	550.390259	50
14161	Fga	fibrinogen alpha chain	1399.69922	1804.78381	50
59083	Fetub	fetuin beta	10194.5391	14402.6641	50
14068	F7	coagulation factor VII	955.160522	1249.17603	50
102022	Ces2a	carboxylesterase 2A	1363.98022	1598.28308	50
225631	Onecut2	one cut domain, family m	168.347977	167.662445	50
235106	Ntm	neurotrimin	130.780304	189.034592	50
53321	Cntnap1	contactin associated prot	412.818268	276.561768	50
27421	Abcc6	ATP-binding cassette, su	166.101593	177.637634	50
11816	Apoe	apolipoprotein E	25954.0176	32831.4883	50
16000	Igf1	insulin-like growth facto	1919.77722	1499.06616	50
21834	Thrb	thyroid hormone recepto	228.769287	257.857788	50
13435	Dnmt3a	DNA methyltransferase 3	106.995155	146.046982	50
228357	Lrp4	low density lipoprotein re	179.047653	149.861053	50
66175	Mustn1	musculoskeletal, embryo	174.803589	233.067688	50
20183	Rxrg	retinoid X receptor gamm	163.615982	135.883636	50
228993	Slc17a9	solute carrier family 17,	263.711975	385.275757	50
217692	Sipa1l1	signal-induced proliferati	255.348969	268.858643	50
16542	Kdr	kinase insert domain pro	401.770081	319.218323	50
13074	Cyp17a1	cytochrome P450, family	2340.36279	2131.39673	50

21835	Thrsp	thyroid hormone respons	2173.34399	2119.21631	50
16917	Lmx1b	LIM homeobox transcrip	4518.52588	6406.38281	50
11848	Rhoa	ras homolog gene family	23641.4297	28182.9023	50
16782	Lamc2	laminin, gamma 2	322.664734	284.709137	50
68509	Ptx4	pentraxin 4	396.429749	283.904175	50
239789	Gm606	predicted gene 606	296.229187	258.133667	50
13819	Epas1	endothelial PAS domain	4080.67896	6061.76855	50
13609	S1pr1	sphingosine-1-phosphate	788.875244	733.893372	50
230766	Fam167b	family with sequence sin	150.256439	176.005035	50
240327	Gm4951	predicted gene 4951	684.685669	891.334473	50
70456	Brp44	brain protein 44	290.730469	202.414993	50
69291	1700001L05Rik	RIKEN cDNA 1700001L	339.8992	280.872406	50
94175	Hrg	histidine-rich glycoprotei	6524.44727	8965.70703	50
240752	Pik3c2b	phosphoinositide-3-kinas	151.013672	187.051163	50
192216	Tmem47	transmembrane protein 4	116.454399	154.269318	50
380713	Scarf1	scavenger receptor class	120.145584	170.042007	50
216198	Tcp11l2	t-complex 11 (mouse) lik	189.642761	221.758423	50
54604	Pcnx	pecanex homolog (Drosc	191.680054	169.171967	50
26464	Vnn3	vanin 3	1400.89209	1141.03491	50
11889	Asgr1	asialoglycoprotein recept	4824.38525	4231.11963	50
13101	Cyp2d10	cytochrome P450, family	4970.7998	5127.66895	50
11988	Slc7a2	solute carrier family 7 (c	1798.18677	2369.44287	50
21789	Tfpi2	tissue factor pathway inh	1107.24829	852.001038	50
327956	Vmo1	vitelline membrane outer	158.988525	134.263794	50
13998	Fgd6	FYVE, RhoGEF and PH	185.847031	265.826904	50
217593	Slc25a21	solute carrier family 25 (	388.152283	273.046204	50
214547	She	src homology 2 domain-c	101.471054	147.942703	50
212933	Pm20d1	peptidase M20 domain co	1500.18018	2087.46143	50
20717	Serpina3m	serine (or cysteine) pepti	2577.9624	3193.72974	50
13143	Dapk2	death-associated protein	102.1586	149.25415	50
235386	Agphd1	aminoglycoside phospho	641.761841	560.0401	50
56189	Prodh2	proline dehydrogenase (c	880.278687	633.547424	50
11287	Pzp	pregnancy zone protein	19714.3457	27766.7148	50
230857	Ece1	endothelin converting en	288.797729	313.533997	50
232493	Gys2	glycogen synthase 2	1600.20166	1900.78516	50
19208	Ptcra	pre T cell antigen recepto	495.296387	482.086304	50
435684	Shf	Src homology 2 domain	185.368607	215.168671	50
67217	2810055F11Rik	RIKEN cDNA 2810055F	354.085693	270.676544	50
11625	Ahsg	alpha-2-HS-glycoprotein	29315.4219	38903.0938	50
240638	Slc16a12	solute carrier family 16 (	762.187805	811.402954	50
11890	Asgr2	asialoglycoprotein recept	12120.5605	12896.8262	50
66968	Plin5	perilipin 5	2024.27734	1484.61475	50
20714	Serpina3k	serine (or cysteine) pepti	7551.63623	9635.15234	50
257633	Acsf3	acyl-CoA synthetase fam	123.702103	153.376633	50
231103	Gckr	glucokinase regulatory pr	6181.71094	9183.13672	50
53376	Usp2	ubiquitin specific peptida	225.661102	248.411713	50
384059	Tlr12	toll-like receptor 12	1510.20654	1463.53406	50
107526	Gimap4	GTPase, IMAP family m	166.68457	233.245941	50
14067	F5	coagulation factor V	6222.18164	7037.0708	50
231510	Agpat9	1-acylglycerol-3-phospha	161.329987	115.767776	50
242341	Atp6v0d2	ATPase, H+ transporting	174.21109	260.581116	50
230801	Pigv	phosphatidylinositol glyc	152.323822	138.19812	50
12038	Bche	butyrylcholinesterase	4352.04395	4870.99219	50
243374	Gimap8	GTPase, IMAP family m	158.50061	176.699677	50
20503	Slc16a7	solute carrier family 16 (	1177.22241	1259.8374	50
71985	Acad10	acyl-Coenzyme A dehyd	190.209915	161.566437	50
72094	Ugt2a3	UDP glucuronosyltransfe	878.261963	666.368896	50
15007	H2-Q10	histocompatibility 2, Q re	14312.7246	20517.7461	50
17777	Mttp	microsomal triglyceride t	1795.9895	2051.12305	50
240328	F830016B08Rik	RIKEN cDNA F830016E	282.994812	195.416382	50
56188	Fxyd1	FXYD domain-containin	9786.4248	13813.876	50

14160	Lgr5	leucine rich repeat contai	383.09079	428.283203	50
13108	Cyp2g1	cytochrome P450, family	458.270508	330.527588	50
192653	Ttc36	tetratricopeptide repeat d	6484.11035	6464.82715	50
68312	Gstm7	glutathione S-transferase	296.782104	391.981506	50
14453	Gas2	growth arrest specific 2	323.954254	279.064392	50
52815	Ldhd	lactate dehydrogenase D	313.832825	360.388062	50
27400	Hsd17b6	hydroxysteroid (17-beta)	3615.07666	4608.46436	50
69721	Nkiras1	NFKB inhibitor interacti	110.51207	129.027924	50
11905	Serpinc1	serine (or cysteine) pepti	945.294189	1365.68799	50
320011	Uggt1	UDP-glucose glycoprote	269.819031	284.962341	50
52076	Tmem38b	transmembrane protein 3	135.995483	144.712891	50
101613	Nlrp6	NLR family, pyrin doma	1084.46277	1198.96777	50
13077	Cyp1a2	cytochrome P450, family	7650.71387	8798.42871	50
66112	Mosc1	MOCO sulphurase C-ter	2366.09961	2620.50146	50
381626	Rbm33	RNA binding motif prote	375.529419	418.27829	50
194590	Reps2	RALBP1 associated Eps	220.171844	314.02774	50
20216	Acsm3	acyl-CoA synthetase med	286.282013	425.838562	50
14411	Slc6a12	solute carrier family 6 (n	516.152344	705.224121	50
14080	Fabp1	fatty acid binding protein	38064.7266	39394.5312	50
74318	Hopx	HOP homeobox	1222.28967	1506.62915	50
12583	Cdo1	cysteine dioxygenase 1, c	13340.709	19445.1484	50
239102	Zfhx2	zinc finger homeobox 2	115.109421	108.548462	50
16562	Kif1c	kinesin family member 1	100.639351	123.957581	50
12124	Bik	BCL2-interacting killer	198.145157	268.618134	50
320311	Rnf152	ring finger protein 152	215.156281	159.979126	50
16597	Klf12	Kruppel-like factor 12	175.904602	245.204559	50
26874	Abcd2	ATP-binding cassette, su	330.300537	277.412018	50
107526	Gimap4	GTPase, IMAP family m	101.784729	122.250008	50
70772	Ggnbp1	gametogenetin binding p	173.21463	184.889465	50
71687	Tmem25	transmembrane protein 2	1278.73291	1689.51257	50
27376	Slc25a10	solute carrier family 25 (	195.6548	184.590973	50
140792	Colec12	collectin sub-family mem	141.693848	155.626038	50
58909	Fam13a	family with sequence sim	148.17955	207.650864	50
12012	Baat	bile acid-Coenzyme A: a	1881.7041	1957.8562	50
106648	Cyp4f15	cytochrome P450, family	4261.14258	5181.50977	50
214579	Aldh5a1	aldhehyde dehydrogenase	290.077759	236.099426	50
11302	Aatk	apoptosis-associated tyro	158.709717	161.654144	50
98496	Pid1	phosphotyrosine interacti	681.791321	989.882324	50
102103	Mtus1	mitochondrial tumor sup	837.316772	856.727661	50
70789	Kynu	kynureninase (L-kynuren	813.97522	899.933105	50
108151	Sema3d	sema domain, immunogl	134.336838	134.341599	50
68126	Fahd2a	fumarylacetoacetate hydr	120.872726	135.322006	50
11522	Adh1	alcohol dehydrogenase 1	22533.6953	29724.9805	50
236149	Slc22a26	solute carrier family 22 (	943.480286	639.011047	50
14060	F13b	coagulation factor XIII, b	9056.43164	11122.6514	50
319848	Slc17a4	solute carrier family 17 (	208.461456	203.219971	50
268860	Abat	4-aminobutyrate aminotr	1993.90186	2420.43213	50
217258	Abca8a	ATP-binding cassette, su	299.850677	398.172302	50
320405	Cadps2	Ca2+-dependent activato	145.86821	186.321167	50
68350	Mul1	mitochondrial ubiquitin l	130.954575	185.418579	50
98256	Kmo	kynurenine 3-monooxyge	6313.77051	7298.96191	50
75396	Spp2	secreted phosphoprotein	5205.94141	6529.77832	50
280662	Afm	afamin	1835.77039	1283.4447	50
104776	Aldh6a1	aldehyde dehydrogenase	1550.71448	1381.08423	50
14261	Fmo1	flavin containing monoo	2004.70605	2326.35205	50
20776	Tmie	transmembrane inner ear	251.169861	356.298096	50
208677	Creb3l3	cAMP responsive elemen	6347.08008	5951.68652	50
69836	Pla2g12b	phospholipase A2, group	6527.04834	8639.17188	50
74129	Dmgdh	dimethylglycine dehydro	1507.1427	1274.56738	50
74156	Acot12	acyl-CoA thioesterase 12	1641.77576	1361.0957	50
55938	Apom	apolipoprotein M	5580.1748	5855.98535	50

15446	Hpgd	hydroxyprostaglandin de	5220.24512	3642.29004	50
12623	Ces1g	carboxylesterase 1G	2230.92163	2757.73486	50
209645	Bend7	BEN domain containing	312.123474	312.01178	50
60344	Fign	fidgetin	507.420227	350.610901	50
14468	Gbp1	guanylate binding protein	174.455048	125.457169	50
76279	Cyp2d26	cytochrome P450, family	9755.9707	7059.45459	50
17195	Mbl2	mannose-binding lectin (	23271.8223	18034.9355	50
16621	Klkb1	kallikrein B, plasma 1	4683.28076	4065.24268	50
56720	Tdo2	tryptophan 2,3-dioxygena	9341.47754	8935.70117	50
18478	Pah	phenylalanine hydroxylas	1139.85742	1708.41748	50
667716	Gm11789	predicted gene 11789	411.791687	556.797546	50
235302	D630033O11Rik	RIKEN cDNA D630033	207.355682	155.658569	50
100504343	Gm20185	predicted gene, 20185	159.058228	120.948471	50
99650	4933434E20Rik	RIKEN cDNA 4933434E	143.895874	193.189621	50
214253	Etnk2	ethanolamine kinase 2	2582.37109	3134.62988	50
71891	Cdadc1	cytidine and dCMP deam	342.145599	440.530396	50
19893	Rpgr	retinitis pigmentosa GTP	115.866669	111.138809	50
30056	Timm9	translocase of inner mito	135.930527	149.331955	50
14728	Lilrb4	leukocyte immunoglobul	100.051613	121.312057	50
23965	Odz3	odd Oz/ten-m homolog 3	170.624481	150.86409	50
14263	Fmo5	flavin containing monoo	549.391968	686.851135	50
12398	Cbfa2t3	core-binding factor, runt	206.333878	257.41922	50
100038730	Gm10387	predicted gene 10387	120.489349	111.171356	50
242484	D630039A03Rik	RIKEN cDNA D630039	363.410278	270.400665	50
12398	Cbfa2t3	core-binding factor, runt	585.13147	474.84436	50
108030	Lin7a	lin-7 homolog A (C. eleg	233.087814	206.615295	50
13085|233005	Cyp2a12|Cyp2a22	cytochrome P450, family	9494.03906	9671.84961	50
121021	Cspg4	chondroitin sulfate prote	208.805237	199.042297	50
11606	Agt	angiotensinogen (serpin p	2685.74658	2086.20801	50
26557	Homer2	homer homolog 2 (Droso	140.583313	120.309021	50
54710	Hs3st3b1	heparan sulfate (glucosar	770.15155	936.097534	50
12491	Cd36	CD36 antigen	334.500244	232.781921	50
19013	Ppara	peroxisome proliferator a	427.223389	501.926331	50
320311	Rnf152	ring finger protein 152	427.731903	424.604919	50
18208	Ntn1	netrin 1	115.383484	147.381058	50
11905	Serpinc1	serine (or cysteine) pepti	28333.4062	29545.7344	50
229900	Gbp7	guanylate binding protein	216.849792	195.085342	50
18366	Olfr64	olfactory receptor 64	210.543091	249.307236	50
319430	Gpr77	G protein-coupled recept	271.233734	389.33316	50
67801	Pllp	plasma membrane proteo	668.347778	500.053223	50
12895|100134861	Cpt1b|BC090627	carnitine palmitoyltransfe	256.245636	294.385803	50
17329	Cxcl9	chemokine (C-X-C motif	241.780304	248.53479	50
319772	C130050O18Rik	RIKEN cDNA C130050	153.016113	159.605637	50
12944	Crp	C-reactive protein, pentra	1784.03992	1996.44141	50
18606	Enpp2	ectonucleotide pyrophosp	689.90564	919.873535	50
71891	Cdadc1	cytidine and dCMP deam	105.680267	123.262955	50
217214	Nags	N-acetylglutamate syntha	536.764343	683.192688	50
59083	Fetub	fetuin beta	824.769958	1088.8645	50
18606	Enpp2	ectonucleotide pyrophosp	146.281677	109.584045	50
433281	Gm5524	predicted gene 5524	208.735535	147.997879	50
104307	Rnu12	RNA U12, small nuclear	130.33197	160.761459	50
320977	A330023F24Rik	RIKEN cDNA A330023	459.322388	617.872314	50
100038628	Gm10768	predicted gene 10768	551.613037	612.942017	50
12895|100134861	Cpt1b|BC090627	carnitine palmitoyltransfe	108.026474	129.85553	50
100041449|56388	Cyp3a59|Cyp3a25	cytochrome P450, subfan	4451.20508	3134.89868	50
11722|109959|545	Amy1|Amy2a5|An	amylase 1, salivaryamyla	7774.81152	8860.40723	50
26365|26367	Ceacam1|Ceacam2	carcinoembryonic antige	2157.89648	2672.18701	50
69627	Fam89a	family with sequence sin	577.60498	477.191406	50.7491302
338350	Acad12	acyl-Coenzyme A dehyd	130.506241	186.311249	50.9118118
26419	Mapk8	mitogen-activated protein	367.853973	453.559937	51.0744972
17329	Cxcl9	chemokine (C-X-C motif	204.675232	207.88147	51.3653564

66113	Apoa5	apolipoprotein A-V	2217.49707	2859.10791	52.0160904
12499	Entpd5	ectonucleoside triphosph	425.824524	512.676758	52.3710365
16169	Il15ra	interleukin 15 receptor, a	601.592896	544.053772	52.4581299
50765	Trfr2	transferrin receptor 2	4438.58105	5244.70264	52.4581299
230661	Tesk2	testis-specific kinase 2	132.130035	108.934685	52.5452232
70373	1700020O03Rik	RIKEN cDNA 1700020O	211.609253	264.91156	52.609314
207781	C2cd2	C2 calcium-dependent do	1259.84302	1412.79517	52.6208115
68338	Golt1a	golgi transport 1 homolo	135.995483	162.67981	52.6849022
76088	Dock8	dedicator of cytokinesis 8	464.741943	457.714966	52.7719955
237387	Lrrc3	leucine rich repeat contai	399.907043	343.514709	52.8771667
15445	Hpd	4-hydroxyphenylpyruvic	4777.01758	4620.89697	53.0628548
19212	Pter	phosphotriesterase relate	237.05307	292.84375	53.3011284
12606	Cebpa	CCAAT/enhancer bindin	2555.12451	2593.61768	53.3126297
11991	Hnrnpd	heterogeneous nuclear ri	231.319855	234.888428	53.7776794
22680	Zfp207	zinc finger protein 207	352.914978	345.663666	53.8532677
77219	Ptgr2	prostaglandin reductase 2	203.002319	210.51709	53.9288559
100041089	Gm3134	predicted gene 3134	323.266724	314.810059	53.9288559
229503	Rrnad1	ribosomal RNA adenine	147.317749	209.458862	54.2427216
26358	Aldh1a7	aldehyde dehydrogenase	6212.08301	7135.21582	54.6206741
72055	Slc38a10	solute carrier family 38,	189.329102	163.936096	54.6321793
20979	Syt1	synaptotagmin I	274.018738	336.56842	54.9345398
66948	Acad8	acyl-Coenzyme A dehyd	139.965485	141.086975	54.9345398
230866	C230096C10Rik	RIKEN cDNA C230096C	136.797089	168.94136	54.9986267
192119	Dicer1	Dicer1, Dcr-1 homolog (	289.599335	420.041016	55.0101318
105171	Arrdc3	arrestin domain containin	1386.67871	1674.05396	55.0857201
19193	Pipox	pipecolic acid oxidase	2207.53735	1740.20898	55.1613083
246103	Atxn7	ataxin 7	257.03772	363.351868	55.1728134
75475	Oplah	5-oxoprolinase (ATP-hyd	1054.95874	1085.93176	55.3239937
111975	Igf2as	insulin-like growth facto	196.372437	238.702499	55.550766
54354	Rassf5	Ras association (RalGDS	136.100037	187.369476	56.0914001
106861	Abhd3	abhydrolase domain cont	785.871582	702.387573	56.3296738
70750	Kdsr	3-ketodihydrosphingosin	164.164124	171.10022	56.7832184
58911	Sumf1	sulfatase modifying facto	167.620819	179.741302	57.097084
26378	Decr2	2-4-dienoyl-Coenzyme A	584.394836	697.056885	57.3468628
93760	Arid1a	AT rich interactive doma	164.442932	127.638672	57.7248154
30935	Tor3a	torsin family 3, member	222.573502	179.819122	57.7889023
72472	Slc16a10	solute carrier family 16 (	799.88855	979.659546	57.7889023
268936	Brpf3	bromodomain and PHD f	154.500504	219.40152	58.1783562
22282	Usf2	upstream transcription fa	401.008057	375.222778	58.1783562
112419	2010002M12Rik	RIKEN cDNA 2010002M	146.590607	207.949371	58.4166298
75863	Clec4g	C-type lectin domain fan	4466.35986	4582.0166	58.4807167
13690	Eif4g2	eukaryotic translation ini	193.547821	190.378571	59.3467216
227696	Phyhd1	phytanoyl-CoA dioxygen	3068.70117	3483.18091	59.573494
11727|11730|2190	Ang|Ang3|Ang4	angiogenin, ribonuclease	7034.62402	6764.40234	60.1256332
234797	6430548M08Rik	RIKEN cDNA 6430548M	352.750244	471.251007	60.3524017
72780	Rspo3	R-spondin 3 homolog (X	151.910339	179.731415	60.4279938
240025	Dact2	dapper homolog 2, antag	137.066406	195.867676	60.5035858
240613	9930021J03Rik	RIKEN cDNA 9930021J	233.666046	213.711517	60.7303543
237542	Osbpl8	oxysterol binding protein	159.676086	176.115387	61.1954002
71750	R3hdm2	R3H domain containing 2	143.965576	200.850311	61.1954002
414085	9330151L19Rik	RIKEN cDNA 9330151L	232.699692	192.826035	61.4336739
414872	Zyg11b	zyg-ll homolog B (C. ele	171.346863	159.0737	61.6719513
231999	Plekha8	pleckstrin homology don	311.530975	213.282852	61.8116226
66391	2310061J03Rik	RIKEN cDNA 2310061J	445.848816	514.017944	62.514946
382051	Pdp2	pyruvate dehyrogenase p	728.190918	989.170654	62.590538
68021	Bphl	biphenyl hydrolase-like (	486.56427	348.231384	62.8813934
69536	Hemk1	HemK methyltransferase	155.53183	223.017517	63.1426773
68943	Pink1	PTEN induced putative k	549.08783	708.265747	63.5091248
171207	Arhgap4	Rho GTPase activating p	227.932831	322.756531	63.5962181
234135	Whsc1l1	Wolf-Hirschhorn syndron	234.611816	311.317139	63.7358971
14852	Gspt1	G1 to S phase transition	142.037613	138.253296	64.1483612

104175	Sbk1	SH3-binding kinase 1	177.77713	209.205627	64.9157639
23806	Arih1	ariadne ubiquitin-conjuga	322.943542	357.080444	65.2181244
246738	Dnajc28	DnaJ (Hsp40) homolog, s	334.579468	388.229675	65.5319901
239796	1600021P15Rik	RIKEN cDNA 1600021P	369.796204	497.11908	65.5319901
66333	Aqp11	aquaporin 11	511.524902	533.35144	65.6190796
103677	Smg6	Smg-6 homolog, nonsens	143.487152	203.716522	66.2123108
66412	Arrdc4	arrestin domain containin	156.67244	223.566437	66.2238083
241274	Pnpla7	patatin-like phospholipas	1833.63013	1485.41968	66.473587
232371	C1rl	complement component	448.787476	417.515747	66.537674
329828	AI464131	expressed sequence AI46	211.5047	166.526428	66.6773529
211446	Exoc3	exocyst complex compon	138.65535	204.531403	66.688858
16880	Lifr	leukemia inhibitory facto	841.52124	761.153564	66.7003555
232146	Fam176a	family with sequence sim	3663.55322	3271.76709	66.8515396
104776	Aldh6a1	aldehyde dehydrogenase	1369.65967	979.436035	66.8630371
235582	Glyctk	glycerate kinase	4945.15674	4851.21729	67.0783081
104910	Slc25a47	solute carrier family 25,	24977.1738	27574.4922	67.2409897
56441	Nat6	N-acetyltransferase 6	153.155518	203.891953	67.3050842
319565	Syne2	synaptic nuclear envelop	174.041595	159.891418	67.5433578
319278	A230050P20Rik	RIKEN cDNA A230050	1670.35156	2232.11914	68.1595764
16971	Lrp1	low density lipoprotein r	4544.71436	4081.36475	68.8398972
54598	Calcrl	calcitonin receptor-like	1066.71826	1302.18823	68.9269867
17777	Mttp	microsomal triglyceride t	1402.6886	1357.43018	69.0781708
217869	Eif5	eukaryotic translation ini	142.416229	178.25444	69.7814941
11807	Apoa2	apolipoprotein A-II	58553.8516	65665.2109	69.7929916
102442	Dennd4a	DENN/MADD domain c	370.223938	449.768494	70.083847
243085	Ugt2b35	UDP glucuronosyltransfe	2367.5127	2267.5437	70.0953522
17920	Myo6	myosin VI	171.625687	159.349579	70.2465363
217351	Tnrc6c	trinucleotide repeat conta	154.639908	226.620819	70.2580414
216874	Camta2	calmodulin binding trans	202.523895	268.803467	70.8512573
21386	Tbx3	T-box 3	3129.11914	2609.94775	70.8627625
11990	Atrn	attractin	287.352936	195.338562	71.4904938
544963	Iqgap2	IQ motif containing GTP	3677.77148	3511.23047	72.0311279
72180	Zfp661	zinc finger protein 661	146.630203	158.479523	72.3334961
57344	As3mt	arsenic (+3 oxidation sta	2458.13208	2685.64355	72.420578
13423	Dnase2a	deoxyribonuclease II alp	152.532928	223.235397	73.1879883
74616	Scrn3	secernin 3	490.355286	549.214661	73.8798065
219189	1300010F03Rik	RIKEN cDNA 1300010F	2072.67456	1383.56152	74.5075378
234733	Ddx19b	DEAD (Asp-Glu-Ala-As	241.252777	282.930817	74.97258
230103	Npr2	natriuretic peptide recept	258.327271	294.684296	76.2806244
240753	Plekha6	pleckstrin homology dom	287.039276	314.634644	76.2806244
67432	Hoga1	4-hydroxy-2-oxoglutarate	1297.45667	1362.01392	76.4433136
213742	Xist	inactive X specific transc	464.363312	499.686829	76.6815872
226418	Yod1	YOD1 OTU deubiquitina	384.296356	429.045715	77.3733978
223672l71898	Apol9alApol9b	apolipoprotein L 9alapoli	1441.4729	1618.59705	77.6642609
319530	Zfp750	zinc finger protein 750	411.443176	611.779175	78.1408081
16008	Igfbp2	insulin-like growth facto	2310.44043	3262.70996	78.2164001
27362	Dnajb9	DnaJ (Hsp40) homolog, s	616.717224	745.716187	79.146492
11606	Agt	angiotensinogen (serpin p	4973.33789	6940.79199	79.2220764
60440	Iigp1	interferon inducible GTP	7088.36279	8578.43945	79.3847656
76261	0610040J01Rik	RIKEN cDNA 0610040J	4032.65869	4502.2627	79.5244446
17388	Mmp15	matrix metallopeptidase	530.462402	762.646057	79.600029
12684	Cideb	cell death-inducing DNA	5237.43652	4733.16504	79.6871262
100503386	Gm4980	predicted gene 4980	897.414917	780.506897	79.8498077
20869	Stk11	serine/threonine kinase 1	166.176056	177.647522	80.1406708
69179	Tmem110	transmembrane protein 1	216.401459	298.794067	81.4602127
227377	Farp2	FERM, RhoGEF and ple	641.338867	727.664307	81.9252625
320940	Atp11c	ATPase, class VI, type 1	504.795197	401.259216	82.7797623
100861531	Rn45s	45S pre-ribosomal RNA	1070.57568	886.289551	83.483078
11761	Aox1	aldehyde oxidase 1	2267.14893	2057.27686	83.622757
71768	Vwce	von Willebrand factor C	284.319214	311.404846	83.6868439
320302	Glt28d2	glycosyltransferase 28 dc	377.392426	518.614319	83.7969513

19263	Ptprb	protein tyrosine phosphat	1132.73975	1285.62207	84.0993042
330790	Hapln4	hyaluronan and proteogly	204.839981	301.364594	84.5643539
15451	Hpn	hepsin	21182.2109	29081.9727	84.6514435
14528	Gch1	GTP cyclohydrolase 1	1561.85144	1975.02673	84.9538116
14871	Gstt1	glutathione S-transferase	4609.16553	6476.35156	85.2676697
14635	Galk1	galactokinase 1	402.42749	290.152954	85.3317642
110265	Msra	methionine sulfoxide red	2166.97852	1861.43042	86.110672
67630	Samd8	sterile alpha motif domai	200.805023	285.855042	86.8024902
17979	Ncoa3	nuclear receptor coactiva	230.318634	315.293884	87.581398
67484	Eepd1	endonuclease/exonucleas	224.1371	335.730927	88.2617188
11529	Adh7	alcohol dehydrogenase 7	289.952606	220.271561	88.8779373
269823	Pon3	paraoxonase 3	465.413635	696.261841	89.3429871
22259	Nr1h3	nuclear receptor subfami	2425.61011	1961.79346	90.1333923
280408	Rilp	Rab interacting lysosoma	254.317657	325.690674	90.1333923
245860	Atg9a	autophagy-related 9A (ye	225.656342	325.479858	90.2089844
320817	Atad2b	ATPase family, AAA do	334.579468	412.513306	90.4472656
21429	Ubtf	upstream binding transcr	331.08313	419.511902	91.7668076
16005	Igfals	insulin-like growth facto	13145.373	12810.2314	91.8308945
71893	Noxo1	NADPH oxidase organiz	397.765198	426.137054	92.057663
103142	Rdh9	retinol dehydrogenase 9	1945.11646	2131.69385	92.2203522
218989	6720456H20Rik	RIKEN cDNA 6720456H	243.125305	271.468781	92.6623917
239570	Ttc38	tetratricopeptide repeat d	939.077759	1340.97852	93.4643097
102093	Phkb	phosphorylase kinase bet	195.211227	234.963409	93.5399017
214137	Arhgap29	Rho GTPase activating p	1222.47498	1628.17468	94.307312
69134	Fam25c	family with sequence sin	3320.19092	4789.40674	94.3828964
12361	Cask	calcium/calmodulin-depe	208.56601	255.157089	94.3828964
207798	Gramd1c	GRAM domain containin	663.595215	977.82605	94.7723541
72129	Pex13	peroxisomal biogenesis f	311.268005	345.641022	95.4756699
66468	Ska1	spindle and kinetochore a	323.84494	404.897888	95.7665253
171210	Acot2	acyl-CoA thioesterase 2	936.257935	895.657837	97.09758
68177	Ebpl	emopamil binding protei	916.198792	799.564575	97.3243484
21355	Tap2	transporter 2, ATP-bindi	979.853455	1199.27893	99.0974426
213989	Tmem82	transmembrane protein 8	536.320801	627.581543	99.4228058
21375	Tbr1	T-box brain gene 1	459.591736	449.447357	99.6380768
78329	2310010J17Rik	RIKEN cDNA 2310010J	272.295135	381.474426	99.6495819
353170	Txlng	taxilin gamma	370.732452	331.894226	99.9634399
67155	Smarca2	SWI/SNF related, matrix	264.524658	293.074341	100.341393
93765	Ube2n	ubiquitin-conjugating enz	227.658768	216.204254	100.579666
252966	Cables2	CDK5 and Abl enzyme s	416.17041	530.621033	100.591171
624219	Gm6484	predicted gene 6484	392.524689	398.668884	100.655258
67727	Stx17	syntaxin 17	305.688446	321.986938	101.422668
12724	Clcn2	chloride channel 2	459.770721	467.933472	101.974808
244416	Ppp1r3b	protein phosphatase 1, re	457.74295	444.246826	102.0504
71918	Zcchc24	zinc finger, CCHC doma	5063.03516	4881.26562	102.201576
20623	Snrk	SNF related kinase	610.570557	721.051941	102.300171
15220	Foxq1	forkhead box Q1	559.5578	739.736145	102.526947
12176	Bnip3	BCL2/adenovirus E1B in	335.6203	406.482361	102.893394
11425	Apoc4	apolipoprotein C-IV	21871.75	17659.4805	103.20726
70503	Ddo	D-aspartate oxidase	294.291748	315.793304	103.20726
15377	Foxa3	forkhead box A3	6983.89453	9637.91992	103.358444
68440	Dusp23	dual specificity phosphat	228.246506	300.524231	103.509621
12572	Cdk7	cyclin-dependent kinase	322.081726	295.463806	104.212944
110616	Atxn3	ataxin 3	218.543304	273.046204	105.207123
74008	Arsg	arylsulfatase G	595.796265	812.471008	105.986031
213988	Tnrc6b	trinucleotide repeat conta	560.305542	803.38855	106.386986
78914	Nadsyn1	NAD synthetase 1	243.773224	364.036621	106.84053
227541	Camk1d	calcium/calmodulin-depe	545.900391	444.334564	107.456757
54338	Slc23a2	solute carrier family 23 (	265.734985	276.110474	107.706535
14555	Gpd1	glycerol-3-phosphate deh	17519.7812	17194.6562	109.479622
66836	Tmem223	transmembrane protein 2	1152.34412	1392.55762	109.566711
231207	Cpeb2	cytoplasmic polyadenyla	637.229431	871.331787	110.031761

232906	Grlf1	glucocorticoid receptor D	281.893799	384.701355	110.473801
219189	1300010F03Rik	RIKEN cDNA 1300010F	2544.32812	1790.42725	110.560898
75869	Arl5b	ADP-ribosylation factor-	283.417786	360.320129	111.037445
19885	Rorc	RAR-related orphan rece	423.009399	567.830933	111.241211
67769	Gpatch2	G patch domain containi	298.261719	407.365173	111.717758
545622	Ptpn3	protein tyrosine phospha	382.577515	362.459167	112.333984
11432	Acp2	acid phosphatase 2, lysos	343.48584	403.395447	113.1129
68750	Rreb1	ras responsive element b	702.473022	523.077759	113.264076
13107	Cyp2f2	cytochrome P450, family	10398.1484	7865.29346	113.688034
15357	Hmgcr	3-hydroxy-3-methylgluta	322.012024	334.848145	114.258255
170716	Cyp4f13	cytochrome P450, family	1124.54614	994.416504	114.420944
16439	Itpr2	inositol 1,4,5-triphosphat	894.55072	1032.46948	115.176842
68777	Tmem53	transmembrane protein 5	1130.49329	823.075745	116.269615
320808	Dcaf5	DDB1 and CUL4 associa	316.831696	353.885986	116.420799
12013	Bach1	BTB and CNC homology	267.837189	375.71936	116.594986
382117	D9Ertd402e	DNA segment, Chr 9, ER	392.320312	578.831787	117.060028
68646	1110020G09Rik	RIKEN cDNA 1110020G	391.757935	351.847382	118.594849
67958	2610101N10Rik	RIKEN cDNA 2610101N	270.367157	375.33313	119.332672
21843	Tial1	Tia1 cytotoxic granule-as	668.476196	777.329468	119.751709
232078	Thnsl2	threonine synthase-like 2	1470.79321	1199.65796	120.984161
269630	5031425E22Rik	RIKEN cDNA 5031425E	304.17395	372.249023	121.158348
77031	Slc9a8	solute carrier family 9 (s	583.756348	825.091675	122.489403
68671	Pcyt2	phosphate cytidylyltransf	593.534058	492.906067	122.617569
219181	Akap11	A kinase (PRKA) anchor	410.248688	462.905579	123.181213
218850	D14Abb1e	DNA segment, Chr 14, A	254.975098	381.130615	124.186897
67867	Lrrc28	leucine rich repeat contai	256.903076	364.74115	124.250984
20338	Sel1l	sel-1 suppressor of lin-12	511.355408	617.839844	124.791626
22318	Vamp2	vesicle-associated memb	423.09021	613.019897	125.33226
102448	Xylb	xylulokinase homolog (H	1439.74927	1426.63391	126.122665
23802	Amfr	autocrine motility factor	1126.18579	1064.18469	126.239342
192166	Sardh	sarcosine dehydrogenase	3610.86426	4921.53125	126.285355
18706	Pik3ca	phosphatidylinositol 3-ki	255.802063	286.549683	126.779976
69215	Sat2	spermidine/spermine N1-	409.117554	420.316895	126.890076
73137	Prrc1	proline-rich coiled-coil 1	425.046692	460.192139	127.872749
100503842	2410022M11Rik	RIKEN cDNA 2410022M	484.492126	434.577271	129.506165
13688	Eif4ebp2	eukaryotic translation ini	452.035095	570.496277	131.529022
72194	Fbxl20	F-box and leucine-rich re	297.195557	360.62854	132.122253
22770	Zhx1	zinc fingers and homeobo	333.687561	489.516418	132.610291
234854	Cdk10	cyclin-dependent kinase	340.377625	347.105286	132.610291
240753	Plekha6	pleckstrin homology dom	691.190369	559.31012	132.848572
66205	Cd302	CD302 antigen	2420.09082	3513.61279	132.999756
212862	Chpt1	choline phosphotransfera	366.274536	303.964844	133.354706
68204	2900060B14Rik	RIKEN cDNA 2900060B	928.30835	1390.87134	135.453156
77733	Rnf170	ring finger protein 170	282.720734	382.454803	135.476166
29809	Rabgap1l	RAB GTPase activating	647.759644	822.86499	136.458832
12751	Tpp1	tripeptidyl peptidase I	504.202728	563.96167	137.17366
52633	Nit2	nitrilase family, member	580.847778	544.572998	137.313339
320808	Dcaf5	DDB1 and CUL4 associa	294.196686	418.057587	137.400436
19934	Rpl22	ribosomal protein L22	468.965393	388.959686	137.615707
278279	Tmtc2	transmembrane and tetrat	709.028442	624.03064	139.952438
14874	Gstz1	glutathione transferase ze	4110.88281	4512.09521	141.196381
213649	Arhgef19	Rho guanine nucleotide e	1123.7002	1405.42737	143.207764
229487	Pet112l	PET112-like (yeast)	307.611664	332.501129	143.335922
53972	Ngef	neuronal guanine nucleot	863.429138	1082.71887	143.975159
68526	Gpr155	G protein-coupled recept	338.414795	331.91684	144.463211
71336	Rbks	ribokinase	390.213318	391.936249	145.132019
380629	Heca	headcase homolog (Dros	543.419556	583.834229	147.218979
70012	Ccdc21	coiled-coil domain conta	497.94043	658.578003	147.608429
27364	Srr	serine racemase	2349.37207	1745.62451	147.910797
56358	Copz2	coatomer protein comple	588.089172	728.391479	151.07901
67201	Glod4	glyoxalase domain conta	347.565125	318.932556	151.793854

50776	Polg2	polymerase (DNA directe	345.836792	328.674316	154.043488
70209	Tmem143	transmembrane protein 1	370.862366	310.599854	154.822388
18128	Notch1	Notch gene homolog 1 (I	607.357788	887.770813	155.037659
240186	Zfp438	zinc finger protein 438	773.465698	1118.89746	156.368713
20318	Sdf4	stromal cell derived facto	423.582886	413.769592	157.060516
64008	Aqp9	aquaporin 9	3944.47119	4431.43799	157.502563
11819	Nr2f2	nuclear receptor subfami	464.741943	641.094971	159.775208
94217	Lrp1b	low density lipoprotein-r	406.292969	413.912476	159.914886
231326	Aasdh	aminoadipate-semialdehy	363.858643	529.855652	160.077576
67207	Lsm1	LSM1 homolog, U6 sma	588.722839	521.59375	160.315842
78255	Ralgps2	Ral GEF with PH domai	691.100098	825.854248	162.304199
21685	Tef	thyrotroph embryonic fac	606.326477	556.080322	163.646759
223646	Naprt1	nicotinate phosphoribosy	4590.65137	5662.1377	164.867706
67422	Dhdds	dehydrodolichyl diphosp	371.350281	398.393005	165.58252
225913	Dak	dihydroxyacetone kinase	1813.73254	1800.15479	165.733704
74764	Klc4	kinesin light chain 4	1281.56714	1189.43384	167.041748
224656	Zfp523	zinc finger protein 523	339.246521	456.130493	167.367126
12339	Capn7	calpain 7	428.793304	531.442993	167.733582
16988	Lst1	leukocyte specific transc	544.86438	704.997681	168.837845
71670	Acy3	aspartoacylase (aminoacy	751.038208	673.438232	169.117203
53621	Cnot4	CCR4-NOT transcription	562.856079	632.872559	169.669342
100504663	Atg14	VATG14 autophagy rela	797.841797	1013.94519	169.832031
110095	Pygl	liver glycogen phosphory	4649.18896	5198.5376	169.832031
17117	Amacr	alpha-methylacyl-CoA ra	646.713989	514.153687	171.854889
212483	Fam193b	family with sequence sin	602.918823	771.048096	174.081512
329650	Med12l	mediator of RNA polyme	487.779358	476.840515	174.319794
14913	Guca1a	guanylate cyclase activat	897.579712	966.990723	176.08139
69870	Polr3gl	polymerase (RNA) III (D	431.253571	483.131775	176.319672
243382	Ppm1k	protein phosphatase 1K (	1748.76294	1982.41162	177.249756
107766	Haao	3-hydroxyanthranilate 3,4	5613.83887	6424.4248	179.941422
213819	Casd1	CAS1 domain containing	381.611145	412.282715	180.202698
21416	Tcf7l2	transcription factor 7 like	496.750641	424.37146	180.330872
71361	Aifm2	apoptosis-inducing factor	412.753296	605.030945	181.03421
320560	Dennd5b	DENN/MADD domain c	658.454529	871.695312	182.039886
98432	Phlpp1	PH domain and leucine r	462.872559	619.183777	183.272339
223631	BC025446	cDNA sequence BC0254	2658.0957	2005.75732	184.591888
71755	Dhdh	dihydrodiol dehydrogena	1675.30371	2076.39258	185.23111
68636	Fahd1	fumarylacetoacetate hydr	608.926147	538.476929	186.213791
170460	Stard5	StAR-related lipid transfe	1624.93262	2294.77539	186.678833
14544	Gda	guanine deaminase	1031.11023	1000.43182	187.132385
15586	Hyal1	hyaluronoglucosaminidas	1016.0318	1460.69751	188.08548
15458	Hpx	hemopexin	22475.9238	31850.582	188.550522
209558	Enpp3	ectonucleotide pyrophosp	476.373108	568.041748	189.968658
382793	Mtx3	metaxin 3	855.275269	710.609924	190.5849
67800	Dgat2	diacylglycerol O-acyltran	3303.48071	2936.69092	193.125397
224585	Zfp160	zinc finger protein 160	598.525879	617.505981	194.834381
52637	Cisd1	CDGSH iron sulfur doma	989.965393	1178.85181	194.886963
66443	Tnfaip8l1	tumor necrosis factor, alp	438.371399	464.067017	195.90416
100504527	Gm10374	predicted gene 10374	882.970215	706.249756	198.694427
270076	Gcdh	glutaryl-Coenzyme A deh	2607.94971	2514.76978	201.08374
76974	1190003J15Rik	RIKEN cDNA 1190003J	12460.1016	13359.2852	202.089432
67017	2010011I20Rik	RIKEN cDNA 2010011I	947.05896	863.249512	203.56015
71782	Ankle2	ankyrin repeat and LEM	560.135986	839.19928	205.873886
54403	Slc4a4	solute carrier family 4 (a	1120.90405	1254.11768	207.862244
107351	Kank1	KN motif and ankyrin re	901.150513	1029.69812	208.274704
227334	Usp40	ubiquitin specific peptida	555.194885	751.36084	209.053604
72108	Ddhd2	DDHD domain containin	558.865479	479.278076	210.02478
29869	Ulk2	Unc-51 like kinase 2 (C.	569.943787	689.990417	214.1987
16438	Itpr1	inositol 1,4,5-trisphospha	667.818726	954.65863	214.547058
71664	Mettl7b	methyltransferase like 7B	26093.7383	23428.1055	215.55275
19883	Rora	RAR-related orphan rece	1315.24084	1295.94385	216.070374

21848	Trim24	tripartite motif-containin	726.720825	683.851929	216.785202
11639	Ak4	adenylate kinase 4	907.073792	612.139893	216.872284
246103	Atxn7	ataxin 7	573.335571	838.387207	217.034973
232232	Hdac11	histone deacetylase 11	953.015564	1022.79712	217.151642
52466	Slc46a1	solute carrier family 46,	587.477661	476.32135	218.25592
381110	Fam82a1	family with sequence sin	745.23053	628.895874	218.668396
15511	Hspa1b	heat shock protein 1B	515.718262	645.882324	219.238602
67393	Cxxc5	CXXC finger 5	591.726501	752.359619	219.889343
208922	Cpeb3	cytoplasmic polyadenyla	488.267273	645.51593	220.535141
71330	Rcbtb1	regulator of chromosome	863.125	907.483459	221.499756
76784	Mtif2	mitochondrial translation	663.983398	677.801208	221.889191
235493	BC031353	cDNA sequence BC0313	1153.12817	1258.21179	223.284332
67815	Sec14l2	SEC14-like 2 (S. cerevis	5835.41309	6561.57422	223.749374
53323	Ube2k	ubiquitin-conjugating en	473.84314	565.899841	224.237427
56738	Mocs1	molybdenum cofactor sy	2100.63403	1923.29895	225.208603
68267	Slc25a22	solute carrier family 25 (	3702.21069	5220.81641	225.371277
56632	Sphk2	sphingosine kinase 2	1325.39722	1960.06604	225.481384
212503	Paox	polyamine oxidase (exo-	662.831665	878.485962	226.010529
13688	Eif4ebp2	eukaryotic translation ini	827.265076	889.491089	226.970184
224826	Ubr2	ubiquitin protein ligase E	673.068726	682.349487	230.26001
77411	Esrp2	epithelial splicing regula	511.16687	658.221497	230.63797
14132	Fcgrt	Fc receptor, IgG, alpha c	7221.70312	6797.57617	231.696228
103140	Gstt3	glutathione S-transferase	9524.61133	10885.4541	233.655014
69993	Chn2	chimerin (chimaerin) 2	522.707764	582.918945	237.16011
54636	Wdr45	WD repeat domain 45	735.861572	871.062988	237.67775
67118	Bfar	bifunctional apoptosis re	789.667358	938.869019	238.660431
232947	Ppp1r37	protein phosphatase 1, re	588.225464	802.651489	239.084381
110821	Pcca	propionyl-Coenzyme A c	491.136261	502.543152	239.386749
68364	0610030E20Rik	RIKEN cDNA 0610030E	515.490112	540.794312	240.997147
18186	Nrp1	neuropilin 1	1312.44946	1413.39502	242.717651
382073	Ccdc84	coiled-coil domain conta	547.982056	625.475037	243.723343
71448	Tmem80	transmembrane protein 8	668.466675	774.200073	243.787415
22034	Traf6	TNF receptor-associated	512.730469	600.44165	244.880188
212073	4831426I19Rik	RIKEN cDNA 4831426I	632.834839	552.665161	244.891693
69020	Zfp707	zinc finger protein 707	594.307129	652.105713	247.322113
68404	Nrn1	neuritin 1	6815.77783	7446.36426	248.902924
18633	Pex16	peroxisomal biogenesis f	1072.79199	906.412476	250.740097
208846	Daam1	dishevelled associated ac	647.908508	905.432129	251.390839
21763	Tex2	testis expressed gene 2	996.779053	937.785278	251.594604
70804	Pgrmc2	progesterone receptor me	955.919373	907.25	252.797485
18618	Pemt	phosphatidylethanolamin	6004.92334	5540.20312	252.891144
72244	1600014C10Rik	RIKEN cDNA 1600014C	3512.41748	3421.54028	254.536057
15258	Hipk2	homeodomain interacting	830.842163	834.693359	254.943588
75352	4930550L24Rik	RIKEN cDNA 4930550L	629.932617	541.92041	255.349472
67528	Nudt7	nudix (nucleoside diphos	1256.56689	917.438843	255.628845
231997	Fkbp14	FK506 binding protein 14	581.321472	824.351807	260.377899
59038	Pxmp4	peroxisomal membrane p	2349.07593	1567.7251	261.738525
18563	Pcx	pyruvate carboxylase	3210.3313	3313.8999	263.639771
209200	Dtx3l	deltex 3-like (Drosophila)	1126.78784	1366.44763	264.842651
320683	Zfp629	zinc finger protein 629	767.138428	1076.97095	265.028351
26363	Btd	biotinidase	1084.39624	1546.93164	267.598419
20315	Cxcl12	chemokine (C-X-C motif	10355.6562	8990.01758	268.301727
17714	Grpel2	GrpE-like 2, mitochondri	677.078369	552.938232	268.656677
68636	Fahd1	fumarylacetoacetate hydr	953.776001	1296.72192	271.818329
99887	Tmem56	transmembrane protein 5	18902.3164	13782.3457	275.893616
244631	Pskh1	protein serine kinase H1	714.856689	753.879089	277.980591
54426	Hgfac	hepatocyte growth factor	2269.28613	3189.31714	278.14328
224647	D17Wsu92e	DNA segment, Chr 17, W	632.128296	687.723999	279.102966
108934	BC024659	cDNA sequence BC0246	673.786377	840.987488	282.241608
72947	Agxt2l2	alanine-glyoxylate amino	728.035645	1017.7395	282.986023
110135	Fgb	fibrinogen beta chain	23522.0117	34436.1562	285.206055

114664	Hsd17b11	hydroxysteroid (17-beta)	1241.95581	854.137329	285.288208
15486	Hsd17b2	hydroxysteroid (17-beta)	5397.71924	5828.70508	285.961975
68631	Cryl1	crystallin, lambda 1	1086.73462	1564.4812	286.717865
70031	Cmtm8	CKLF-like MARVEL tra	582.028076	793.860352	287.444183
14186	Fgfr4	fibroblast growth factor r	1109.43298	1109.19678	287.455688
15525	Hspa4	heat shock protein 4	971.884888	767.973877	288.979004
114229	Kiss1r	KISS1 receptor	2847.15088	3111.2417	291.402832
76574	Mfsd2a	major facilitator superfan	4959.24512	3776.64429	297.001465
14431	Gamt	guanidinoacetate methylt	1014.54108	1091.974	302.843292
80283	Abtb1	ankyrin repeat and BTB	2260.19775	2354.53882	304.84314
59007	Ngly1	N-glycanase 1	973.337646	1125.13208	306.151184
58223	Mmp19	matrix metallopeptidase	2448.41626	2643.20508	307.081299
70484	Slc35d2	solute carrier family 35,	1585.98022	1168.99976	311.947021
320191	Hook3	hook homolog 3 (Drosop	1538.83777	1595.19189	313.080872
14073	Faah	fatty acid amide hydrolas	2024.47522	1798.20398	317.719788
328133	Slc39a9	solute carrier family 39 (	828.709839	970.905273	318.638397
74781	Wipi2	WD repeat domain, phos	1059.3374	1287.1853	319.89386
268566	Gphn	gephyrin	1890.51392	2105.69287	323.160675
74525	8430419L09Rik	RIKEN cDNA 8430419L	2841.21191	2242.1665	323.387451
13382	Dld	dihydrolipoamide dehydr	952.397705	756.888184	324.835175
68066	Slc25a39	solute carrier family 25,	945.477905	1344.19556	325.172058
73166	Tm7sf2	transmembrane 7 superfa	1026.01379	1132.10107	329.758423
434402	Gm5617	predicted gene 5617	765.090088	1016.87231	331.538086
100383	Bsdc1	BSD domain containing	894.748779	1056.50415	334.555115
18634	Pex7	peroxisomal biogenesis f	921.432983	793.578857	334.729309
212647	Aldh4a1	aldehyde dehydrogenase	3579.90283	2428.37305	334.926514
50850	Spast	spastin	744.359253	980.429199	339.21051
20181	Rxra	retinoid X receptor alpha	3168.29492	3030.76831	339.803711
12411	Cbs	cystathionine beta-syntha	2297.73047	1927.06909	341.391113
235132	Zbtb44	zinc finger and BTB dom	2161.9248	2440.85352	342.768188
108077	Skiv2l	superkiller viralicidic act	708.957153	1030.90491	351.796326
14081	Acsl1	acyl-CoA synthetase long	10363.2715	9082.23242	354.900452
20186	Nr1h4	nuclear receptor subfami	5198.62988	5522.80371	358.336548
20502	Slc16a2	solute carrier family 16 (	1187.61304	1181.94434	359.330719
18569	Pdcd4	programmed cell death 4	1774.74231	2499.66479	365.962952
18553	Pcsk6	proprotein convertase sul	4025.89087	3072.28613	366.212738
20969	Sdc1	syndecan 1	1093.27417	1320.19482	366.450989
69834	Rab43	RAB43, member RAS on	2374.45166	2398.3667	367.369598
11938	Atp2a2	ATPase, Ca++ transporti	1060.99609	1222.4718	370.386658
67732	Iah1	isoamyl acetate-hydrolyz	2851.09863	2553.38184	374.532623
12411	Cbs	cystathionine beta-syntha	1500.77271	1239.51343	375.217834
100037258	Dnajc3	DnaJ (Hsp40) homolog,	2892.60449	2410.9082	375.316467
320541	Slc35e2	solute carrier family 35,	1647.60559	1585.82373	376.235046
211922	Fam116a	family with sequence sin	1324.80786	1678.3208	378.257904
67101	2310039H08Rik	RIKEN cDNA 2310039H	1226.14709	1195.58789	380.408936
15259	Hipk3	homeodomain interacting	1444.29126	1776.6012	381.286438
67914	Coq9	coenzyme Q9 homolog (	2125.69287	1765.17871	381.955261
66586	Crls1	cardiolipin synthase 1	793.513733	819.346558	382.11795
320415	Gchfr	GTP cyclohydrolase I fee	4393.86865	3773.43311	393.732605
30934	Tor1b	torsin family 1, member	902.824951	1069.79968	396.255005
18408	Slc25a15	solute carrier family 25 (	4822.61719	6091.95312	400.511078
13113	Cyp3a13	cytochrome P450, family	4226.4502	6161.18506	402.865906
110446	Acat1	acetyl-Coenzyme A acety	2029.54785	1392.4303	403.248779
104009	Qsox1	quiescin Q6 sulfhydryl o	2175.26709	2894.75171	404.847656
67880	Dcxr	dicarbonyl L-xylulose re	1387.26807	1180.13635	407.47525
235293	Sc5d	sterol-C5-desaturase (fun	1159.91187	1175.96155	409.78241
246277	Csad	cysteine sulfinic acid dec	14201.416	13701.5469	411.346802
15500	Hsf2	heat shock factor 2	1157.12683	976.213257	414.299744
67384	Bag4	BCL2-associated athanog	1114.51343	1062.96655	414.805908
108958	Fam73b	family with sequence sin	1175.30396	1637.36182	414.897919
231670	Fbxo21	F-box protein 21	1649.51611	1138.86621	418.141724

65116	Prrg2	proline-rich Gla (G-carbo	875.991821	1090.67529	431.774292
142688	Asb13	ankyrin repeat and SOCS	1824.21997	1435.75464	436.621948
75578	Fggy	FGGY carbohydrate kina	2392.04736	1836.45789	439.075378
72144	Slc37a3	solute carrier family 37 (	1003.1554	1032.22339	445.650085
16432	Itm2b	integral membrane protei	3457.78027	3462.44116	451.505066
58809	Rnase4	ribonuclease, RNase A fa	22701.9199	29873.4805	455.660889
71898///223672	Apol9b///Apol9a	apolipoprotein L 9b///apoli	1390.04028	1326.59497	456.190002
74440	4933407C03Rik	RIKEN cDNA 4933407C	937.016724	1160.19312	459.71814
20181	Rxra	retinoid X receptor alpha	4146.5498	4264.89453	462.084442
20334	Sec23a	SEC23A (S. cerevisiae)	1686.56897	2109.61572	463.56665
226252	Fam160b1	family with sequence sim	960.920654	1058.08154	465.536926
66146	Tmem57	transmembrane protein 5	1644.70337	1545.41797	469.751953
74178	Stk40	serine/threonine kinase 4	1908.77966	1519.2146	471.321259
16922	Phyh	phytanoyl-CoA hydroxyl	6675.09473	9557.85938	473.763153
20411	Sorbs1	sorbin and SH3 domain c	1569.75183	2169.90137	479.110352
104174	Gldc	glycine decarboxylase	2418.2229	3133.31543	481.721497
68477	Rmnd5a	required for meiotic nucl	2118.6543	2478.64795	482.62204
353156	Egfl7	EGF-like domain 7	2320.30835	3258.48975	485.888855
52588	Tspan14	tetraspanin 14	1077.76953	1421.53247	488.615051
225010	Lclat1	lysocardiolipin acyltransf	1760.85352	1651.92639	492.493164
74754	Dhcr24	24-dehydrocholesterol re	2813.604	2466.92676	493.173462
12509	Cd59a	CD59a antigen	1962.68518	2046.91138	494.03949
207304	Hectd1	HECT domain containing	1364.45874	1450.40125	497.31781
52633	Nit2	nitrilase family, member	1316.75854	1273.67188	500.568207
102294	Cyp4v3	cytochrome P450, family	11565.3496	10629.9453	502.433289
110391	Qdpr	quinoid dihydropteridine	2645.88965	2795.21362	510.984894
11639	Ak4	adenylate kinase 4	3206.58496	2221.25391	511.956055
235043	Tmem205	transmembrane protein 2	9080.33789	6180.95605	516.762573
80285	Parp9	poly (ADP-ribose) polym	1517.52551	1461.55066	516.879272
66972	Slc25a23	solute carrier family 25 (	2200.73779	2361.70459	518.251404
67302	Zc3h13	zinc finger CCCH type c	1273.75073	1539.60339	524.697998
226791	Lyplal1	lysophospholipase-like 1	1555.45435	1771.57324	526.070068
140630	Ube4a	ubiquitination factor E4A	1800.95752	2065.82324	527.337036
11798	Xiap	X-linked inhibitor of apo	1135.32031	1267.29285	529.616333
56690	Mlycd	malonyl-CoA decarboxyl	9857.61133	8986.04492	530.040283
67095	Trak1	trafficking protein, kines	1229.72266	1245.64917	537.405396
72278	Ccpg1	cell cycle progression 1	2585.33203	3854.49048	540.64917
74334	Ranbp10	RAN binding protein 10	3112.35522	3875.14111	541.288452
28042	Ept1	ethanolaminephosphotran	1480.08142	2066.06665	543.061523
12892	Cpox	coproporphyrinogen oxid	3037.68872	3733.74854	544.154297
216440	Os9	amplified in osteosarcom	1123.61475	1536.58728	547.095764
11981	Atp9a	ATPase, class II, type 9A	2263.18555	2954.94775	556.222473
272589	Tbcel	tubulin folding cofactor E	1677.13196	1618.38196	557.44342
69129	Pex11c	peroxisomal biogenesis f	2316.60303	1893.82056	558.623291
52065	Mfhas1	malignant fibrous histioc	1173.20325	1347.04333	558.914185
15490	Hsd17b7	hydroxysteroid (17-beta)	2902.38721	3380.33105	561.111206
13804	Endog	endonuclease G	2495.96899	2973.14697	562.187561
212862	Chpt1	choline phosphotransfera	1976.9541	1743.72168	562.657532
270166	Clpx	caseinolytic peptidase X	2449.73877	2839.34985	566.936646
223267	A2ld1	AIG2-like domain 1	1260.87439	1447.57031	577.731262
57435	Plin4	perilipin 4	1322.08459	1825.4585	579.062317
20425	Shmt1	serine hydroxymethyltran	1648.15381	1319.44507	584.89917
110198	Akr7a5	aldo-keto reductase famil	2795.45361	2409.31812	586.049438
57279	Slc25a20	solute carrier family 25 (	3512.26392	2852.29883	586.422485
18569	Pdcd4	programmed cell death 4	2374.61304	3289.83447	587.631958
70510	Rnf167	ring finger protein 167	1331.26978	1598.88574	589.30481
109299	C330006A16Rik	RIKEN cDNA C330006A	1398.10071	1364.05249	596.415222
67155	Smarca2	SWI/SNF related, matrix	2135.67651	2681.53271	602.501953
228607	Mavs	mitochondrial antiviral si	1250.10815	1284.67969	613.087891
73389	Hbp1	high mobility group box	1706.04663	1674.59448	637.224243
66552	2010106G01Rik	RIKEN cDNA 2010106G	1393.62219	1290.38965	638.159241

67636	Lyrm5	LYR motif containing 5	2325.62793	2008.97705	645.036377
69632	Arhgef12	Rho guanine nucleotide e	1470.69666	1813.86621	645.244995
664994	Isoc2a	isochorismatase domain c	1314.49634	1663.15784	649.512573
13528	Dtnb	dystrobrevin, beta	1470.79639	2109.65137	652.151672
23972	Papss2	3'-phosphoadenosine 5'-p	2925.69702	3667.86377	652.465576
13177	Eci1	enoyl-Coenzyme A delta	4209.4248	3310.53296	657.848938
70335	Reep6	receptor accessory protei	18253.3555	23482.1035	659.603882
14287	Fpgs	folylpolyglutamyl synthe	1350.29919	1405.84473	660.156067
14962	Cfb	complement factor B	45025.6797	53988.0664	660.400879
170459	Stard4	StAR-related lipid transfe	1668.59302	1888.33838	670.045288
13850	Ephx2	epoxide hydrolase 2, cyto	44938.4023	32613.1523	676.026733
27376	Slc25a10	solute carrier family 25 (	1633.5	2235.73364	682.264648
214627	Papd5	PAP associated domain c	1436.74878	1898.28955	682.862793
28199	Dcaf11	DDB1 and CUL4 associa	3826.59399	4400.26611	693.675537
16432	Itm2b	integral membrane protei	3414.99072	4070.75854	704.795532
22117	Tst	thiosulfate sulfurtransfera	4891.43848	5045.56543	705.342773
100046684l28295	LOC100046684lD	ES1 protein homolog, mi	1675.5509	1696.14197	707.708984
58887	Repin1	replication initiator 1	1485.23169	1839.61694	708.888916
55951	Brp44l	brain protein 44-like	2381.89404	3017.31006	711.687378
66273	1810020D17Rik	RIKEN cDNA 1810020D	1451.44067	2027.79565	712.847534
224824	Pex6	peroxisomal biogenesis f	4425.0293	3731.48364	724.396484
16548	Khk	ketohexokinase	7964.07422	9458.14258	727.793091
27399	Ip6k1	inositol hexaphosphate k	1519.23804	2033.39648	732.885559
14600	Ghr	growth hormone receptor	2831.53076	2128.18262	740.832397
319263	Pcmtd1	protein-L-isoaspartate (D	3495.29077	4259.41553	741.187378
233016	Blvrb	biliverdin reductase B (fl	18166.9844	18652.8633	750.992798
68024	Hist1h2bc	histone cluster 1, H2bc	4577.18555	6184.19727	758.313538
66790	Grtp1	GH regulated TBC prote	1776.52441	2247.30762	766.263672
66885	Acadsb	acyl-Coenzyme A dehyd	1613.97949	1880.37793	770.851685
217463	Snx13	sorting nexin 13	2016.578	2498.10449	770.950317
19662	Rbp4	retinol binding protein 4,	4052.13623	4596.04199	775.792969
57279	Slc25a20	solute carrier family 25 (	2884.12305	2174.17236	780.134521
21452	Tcn2	transcobalamin 2	2076.36572	2949.91016	829.756409
13202	Ddt	D-dopachrome tautomera	6461.34863	9691.35938	831.563965
28169	Agpat3	1-acylglycerol-3-phospha	2274.53931	1811.35376	858.950745
68778	1110038D17Rik	RIKEN cDNA 1110038D	2837.04395	2995.64941	863.58313
68728	Trp53inp2	transformation related pr	5483.72559	5007.16602	864.158264
229003	BC006779	cDNA sequence BC0067	3722.1145	4106.54395	866.820312
235439	Herc1	hect (homologous to the	2974.50464	2396.1499	871.36731
108156	Mthfd1	methylenetetrahydrofolat	1788.67371	2253.68652	875.04657
239743	Klhl6	kelch-like 6 (Drosophila)	2017.55872	2107.46704	887.410522
15529	Sdc2	syndecan 2	7581.4668	8058.92871	909.251221
12040	Bckdhb	branched chain ketoacid	8930.25195	9978.89844	935.683228
13036	Ctsh	cathepsin H	8866.05078	9012.18262	946.137756
68180	Hyi	hydroxypyruvate isomera	6778.32422	5575.72266	950.839111
140740	Sec63	SEC63-like (S. cerevisiae	1959.26331	2485.16553	961.691284
15356	Hmgcl	3-hydroxy-3-methylgluta	15003.0498	10306.375	990.869202
67836	Wdr83	WD repeat domain conta	2208.08838	2291.72827	1012.67377
17117	Amacr	alpha-methylacyl-CoA ra	3978.31738	4323.92773	1018.23792
214597	Sidt2	SID1 transmembrane fam	2667.50122	3423.69189	1030.97327
15199	Hebp1	heme binding protein 1	11255.5195	11303.3809	1038.37939
22661	Zfp148	zinc finger protein 148	2389.44922	2355.3042	1047.46509
69635	Dapk1	death associated protein k	2690.98682	3279.1123	1065.79419
235674	Acaa1b	acetyl-Coenzyme A acylt	34858.2695	27056.0859	1070.63696
192197	Bcas3	breast carcinoma amplifie	2291.33838	3286.07959	1071.76416
64384	Sirt3	sirtuin 3 (silent mating ty	4301.28125	5555.99561	1092.6748
66349	Atp5sl	ATP5S-like	2743.17383	2896.32495	1099.46472
17158	Man2a1	mannosidase 2, alpha 1	4257.79688	5549.93359	1108.86597
66904	Pccb	propionyl Coenzyme A c	4231.21387	4521.97559	1119.82983
74159	Acbd5	acyl-Coenzyme A bindin	3109.73804	2897.54004	1141.71484
57875	Angptl4	angiopoietin-like 4	6672.93701	9018.84277	1146.80737

12633	Cflar	CASP8 and FADD-like a	2403.80347	2565.22559	1148.96509
100727	Ugt2b34	UDP glucuronosyltransfe	3147.63525	2789.56323	1149.31348
14854	Gss	glutathione synthetase	2348.64819	3030.4502	1161.53271
76491	Abhd14b	abhydrolase domain cont	2386.82715	3081.17749	1171.04236
20656	Sod2	superoxide dismutase 2,	3103.02905	3433.86963	1199.05688
93721	Cpn1	carboxypeptidase N, poly	9510.39746	13364.0039	1296.14783
11946	Atp5a1	ATP synthase, H+ transp	2899.18066	3244.50098	1316.07739
66890	Lman2	lectin, mannose-binding 2	5884.12695	4965.30566	1350.60583
23821	Bace1	beta-site APP cleaving e	3602.74365	3296.99561	1362.87769
56876	Nelf	nasal embryonic LHRH f	6422.48975	7427.90674	1381.78369
74148	1300001I01Rik	RIKEN cDNA 1300001I	5420.05811	5479.37451	1388.25146
13601	Ecm1	extracellular matrix prote	3453.5835	3353.00708	1389.27026
76408	Abcc3	ATP-binding cassette, su	3795.37256	4517.02539	1414.30054
20917	Suclg2	succinate-Coenzyme A li	3281.17383	4090.7854	1430.92725
12894	Cpt1a	carnitine palmitoyltransfe	7801.28516	11602.3525	1431.53027
14085	Fah	fumarylacetoacetate hydr	14215.6133	20951.0742	1482.62292
68917	Hint2	histidine triad nucleotide	9683.99219	8048.03906	1486.93652
19305	Pex5	peroxisomal biogenesis f	3636.47754	4491.41797	1513.18286
70361	Lman1	lectin, mannose-binding,	3109.18848	3184.71777	1531.19141
19301	Pxmp2	peroxisomal membrane p	9699.36914	9583.78711	1531.67285
12780	Abcc2	ATP-binding cassette, su	5650.38184	6397.67188	1543.98096
19268	Ptprf	protein tyrosine phospha	4319.33691	5157.42285	1574.38965
12266	C3	complement component 3	36598.5117	47907.4297	1580.3186
73724	Mcee	methylmalonyl CoA epin	3341.00879	4976.7749	1615.29236
27981	D4Wsu53e	DNA segment, Chr 4, W	6598.14697	6974.5293	1622.82507
14860	Gsta4	glutathione S-transferase	3680.95068	3772.8374	1623.51196
69574	Cmbl	carboxymethylenebuteno	7220.53564	6872.44336	1643.05371
50799	Slc25a13	solute carrier family 25 (	7119.96582	7329.59375	1643.30518
29811	Ndrg2	N-myc downstream regu	60692.1133	62133.7148	1648.67542
22134	Tgoln1	trans-golgi network prote	3959.64136	4312.66553	1661.81995
67452	Pnpla8	patatin-like phospholipas	4809.94727	5992.17188	1663.30225
14651	Hagh	hydroxyacyl glutathione	4845.43457	6972.12402	1681.61316
56752	Aldh9a1	aldehyde dehydrogenase	5813.29395	5762.7666	1725.39307
230249	AI314180	expressed sequence AI31	3637.78613	3746.72876	1738.98462
208884	Zdhhc9	zinc finger, DHHC doma	5891.66113	6104.1123	1761.37085
15488	Hsd17b4	hydroxysteroid (17-beta)	7184.96387	4920.7041	1776.00928
211389	Suox	sulfite oxidase	9515.08008	10090.0957	1807.021
14645	Glul	glutamate-ammonia ligas	10078.7344	10861.7148	1820.25427
51798	Ech1	enoyl coenzyme A hydra	10560.3242	8522.71973	1862.99072
100502992l20280	Gm19491lScp2	predicted gene, 19491lste	15690.2021	11628.293	1879.94775
78920	Dlst	dihydrolipoamide S-succ	4094.6167	5123.20117	1917.72803
73192	Xpot	exportin, tRNA (nuclear	5556.7002	6132.7793	1928.86621
217069	Trim25	tripartite motif-containin	5735.76465	7958.29248	1984.25757
12460	Ccs	copper chaperone for sup	8400.47168	11714.0957	1990.8125
72999	Insig2	insulin induced gene 2	10764.3145	12566.1719	1992.77795
11370	Acadvl	acyl-Coenzyme A dehyd	12938.7002	12432.5039	2004.56519
13849	Ephx1	epoxide hydrolase 1, mic	5250.54102	6969.00488	2038.62024
70984	4931406C07Rik	RIKEN cDNA 4931406C	10842.1475	9986.63086	2120.5144
18035	Nfkbia	nuclear factor of kappa li	6857.57861	8629.6416	2177.74463
14828	Hspa5	heat shock protein 5	5116.59082	5764.19824	2242.62573
14473	Gc	group specific componen	11648.6348	17316.6641	2275.8938
15473	Hrsp12	heat-responsive protein 1	15376.2822	12922.7969	2291.1582
18082	Nipsnap1	4-nitrophenylphosphatase	9170.51562	12629.8691	2316.18677
16010	Igfbp4	insulin-like growth factor	12931.5557	16646.7539	2332.50439
11655	Alas1	aminolevulinic acid synth	12460.6748	14272.0283	2345.18896
74114	Crot	carnitine O-octanoyltrans	21497.1406	17274.3828	2398.70361
238055	Apob	apolipoprotein B	14862.5879	19347.9688	2414.20288
58875	Hibadh	3-hydroxyisobutyrate deh	6479.65723	8707.10254	2425.95557
23986	Eci2	enoyl-Coenzyme A delta	9487.41797	6908.33008	2632.22559
217615	Ctage5	CTAGE family, member	7087.7041	8398.00391	2808.09155
64209	Herpud1	homocysteine-inducible,	14149.3398	20928.4258	2863.43359

53945	Slc40a1	solute carrier family 40 (	17442.9375	18887.2031	3087.62012
113868	Acaa1a	acetyl-Coenzyme A acylt	12772.25	11770.5879	3124.4541
56615	Mgst1	microsomal glutathione S	12267.1064	9509.38672	3164.51709
12896	Cpt2	carnitine palmitoyltransfe	21977.1289	14835.3193	3208.0686
57436	Gabarapl1	gamma-aminobutyric aci	10044.8281	13311.1221	3221.03076
11430	Acox1	acyl-Coenzyme A oxidas	13612.6582	16231.4395	3344.74121
11364	Acadm	acyl-Coenzyme A dehyd	20127.1211	14720.0156	3400.06836
27973	Vkorc1	vitamin K epoxide reduc	11726.043	12399.4668	3657.93359
15473	Hrsp12	heat-responsive protein 1	28073.873	19593.125	3958.26123
67125	Tspan31	tetraspanin 31	8038.1499	8075.72412	4000.60156
19299	Abcd3	ATP-binding cassette, su	28246.4375	19228.2051	4051.77148
14600	Ghr	growth hormone receptor	14200.9746	13495.5322	4493.60254
11534	Adk	adenosine kinase	10073.7227	14015.4043	4955.31055
110213	Tmbim6	transmembrane BAX inh	11330.9453	13882.0752	5005.15137
66092	Ghitm	growth hormone inducib	10572.4473	12834.0879	5272.12988
71900	Tmem106b	transmembrane protein 1	15437.873	16232.6035	5340.8335
76238	Grhpr	glyoxylate reductase/hyd	19756.8086	19426.6328	5724.86133
56018	Stard10	START domain containi	13550.0566	18595.168	5937.09766
103172	Chchd10	coiled-coil-helix-coiled-c	20447.5234	22940.8574	6042.87305
101502	Hsd3b7	hydroxy-delta-5-steroid d	30313.2559	25661.1055	6657.59277
18242	Oat	ornithine aminotransferas	35267.5547	29672.2051	6711.43799
57874	Ptplad1	protein tyrosine phosphat	15874.5127	20008.6484	7067.60205
14661	Glud1	glutamate dehydrogenase	56500.0977	51925.8555	7726.37793
14859	Gsta3	glutathione S-transferase	31050.5449	38174.0586	8070.07227
68465	Adipor2	adiponectin receptor 2	34666.9258	35141.5039	8213.96094
57874	Ptplad1	protein tyrosine phosphat	19765.9922	19155.998	8255.71973
107503	Atf5	activating transcription fa	30668.832	39056.1016	8504.11621
14775	Gpx1	glutathione peroxidase 1	18224.084	19215.7031	8647.5918
52538	Acaa2	acetyl-Coenzyme A acylt	58482.6641	60199.0938	9620.22461
15107	Hadh	hydroxyacyl-Coenzyme A	25414.0977	27484.125	11338.5996
11669	Aldh2	aldehyde dehydrogenase	36541.8281	35247.5117	11820.4688
68801	Elovl5	ELOVL family member	35787.543	42984.6797	12511.3457
22041	Trf	transferrin	31044.0117	42361.9453	14671.0234
19662	Rbp4	retinol binding protein 4,	37808.9648	45243.7891	16254.9609
20363	Sepp1	selenoprotein P, plasma,	62336.0312	65664.625	23347.7969

Supplementary Table 2: List of genes participate in Glutathion pathway.

Group	Entrez Gene	Gene symbol	Description	Normalize Value		
				Normal liver	Gnmt-/- HCC	Ymac-1
Up regulation	20810	Srm	spermidine synthase	804.56665	933.604858	7861.4609
	14381	G6pdx	glucose-6-phosphate dehy	50	193.773895	454.02091
	110208	Pgd	phosphogluconate dehydro	117.625122	502.523315	1638.8667
	382985	Rrm2b	ribonucleotide reductase M	81.3770905	208.423309	454.21317
	72692	Hnrpll	heterogeneous nuclear ribo	866.386841	1288.99316	4632.8223
	14862	Gstm1	glutathione S-transferase	50	50	135.91821
	66073	Txndc12	thioredoxin domain contai	1032.11621	1142.88831	2894.1958
	20135	Rrm2	ribonucleotide reductase M	50	447.213501	3187.5278
	20133	Rrm1	ribonucleotide reductase M	50	50	714.13257
	68214	Gsto2	glutathione S-transferase o	50	50	356.16248
	14873	Gsto1	glutathione S-transferase o	12182.5176	11097.4551	36592.375
	14870	Gstp1	glutathione S-transferase	2667.90039	2870.19092	8398.4141
	211666	Mgst2	microsomal glutathione S-	50	70.4105072	260.69833
Down regulation	14854	Gss	glutathione synthetase	2348.64819	3030.4502	1161.5327
	14775	Gpx1	glutathione peroxidase 1	18224.084	19215.7031	8647.5918
	14859	Gsta3	glutathione S-transferase	31050.5449	38174.0586	8070.0723
	14860	Gsta4	glutathione S-transferase	3680.95068	3772.8374	1623.512
	75475	Oplah	5-oxoprolinase (ATP-hydr	1054.95874	1085.93176	55.323994
	76263	Gstk1	glutathione S-transferase k	7946.54053	5457.95166	50
	14871	Gstt1	glutathione S-transferase	4609.16553	6476.35156	85.26767
	14874	Gstz1	glutathione transferase zet	4110.88281	4512.09521	141.19638
	16790	Anpep	alanyl (membrane) aminop	163.650818	154.291962	50
	56615	Mgst1	microsomal glutathione S-	12267.1064	9509.38672	3164.5171
	68312	Gstm7	glutathione S-transferase	296.782104	391.981506	50

Supplementary Table 3. G6PD and CD133 expression in Tumor (T) versus tumor-adjacent (TA) tissues from 37 HCC patients and the associations with their clinical characteristics.

		G6PD expression (T/TA)			CD133 expression (T/TA)		
		Low	High	*P* value[c]	Low	High	*P* value
**Age**				0.823			0.098
	< 60	7	6		5	8	
	≥ 60	12	12		16	8	
**Gender**				0.248			0.236
	Female	8	11		9	10	
	Male	11	7		12	6	
**Cirrhosis**				0.909			0.565
	Negative	13	12		15	10	
	Positive	6	6		6	6	
**Viral infection**[a]				0.281			0.983
	NBNC	6	5		6	5	
	HBV	8	4		7	5	
	HCV	5	9		8	6	
**Tumor size (cm)**				0.419			**0.039**
	< 5	12	9		15	6	
	≥ 5	7	9		6	10	
**AFP (ng/ml)**				0.254			
	< 20	12	8		12	8	0.666
	≥ 20	7	10		9	8	
**TNM**[b] **stage**				**0.033**			**0.005**
	Early	13	6		15	4	
	Late	6	12		6	12	
**Tumor type**				0.092			**0.041**
	Solitary	17	12		19	10	
	Multiple	2	6		2	6	
**PVTT**				0.072			**0.015**
	Negative	13	7		15	5	
	Positive	6	11		6	11	

[a]HBV, HBV sAg (+); HCV, anti-HCV antibody (+); NBNC, HBV sAg (−) and anti-HCV antibodies (−).

[b]Early stage, TNM stage = I; Late stage, beyond TNM stage I (TNM stage = II+IIIA+IIIB+IIIC+IV).

[c]Estimated by χ^2 test.

Supplementary Table 4. Characteristics and univariate survival analysis of 37 HCC cases

		Case No.	Median OS[c] (years)	*P* value[e]
**Age**				0.44
	< 60	13	2.858	
	≥ 60	24	—[d]	
**Gender**				0.246
	Female	19	—[d]	
	Male	18	—[d]	
**Cirrhosis**				0.311
	Negative	25	—[d]	
	Positive	12	2.858	
**Viral infection**[a]				0.974
	NBNC	11	—[d]	
	HBV	12	—[d]	
	HCV	14	—[d]	
**Tumor size (cm)**				0.057
	< 5	21	—[d]	
	≥ 5	16	2.542	
**AFP (ng/ml)**				0.649
	< 20	20	—[d]	
	≥ 20	17	—[d]	
**TNM[b] stage**				**0.014**
	Early	19	—[d]	
	Late	18	2.542	
**Tumor type**				**0.003**
	Solitary	29	—[d]	
	Multiple	8	1.194	
**PVTT**				**0.007**
	Negative	20	—[d]	
	Positive	17	2.514	

**G6PD expression**				**0.003**
	Low	19	—[d]	
	High	18	1.617	
**CD133 expression**				**<0.0001**
	Low	21	—[d]	
	High	16	1.617	

[a]HBV, HBV sAg (+); HCV, anti-HCV antibody (+); NBNC, HBV sAg (−) and anti-HCV antibodies (−).

[b]Early stage, TNM stage = I; Late stage, beyond TNM stage I (TNM stage = II+IIIA+IIIB+IIIC+IV).

[c]OS, overall survival.

[d]Median survival was not reached.

[e]Estimated by log-rank test.

Supplementary Table 5. The comparison of gene expression profile in SH-J1 and Ymac-1.

Official symbol		Full name	Expression in SH-J1	Expression in Ymac-1[a]	References
Human	Mouse				
EPCAM	Epcam	Epithelial cell adhesion molecule	Non-detectable	Ymac-1 << HCC[b]	[4]
SRF	Srf	Serum response factor	Ddetectable	Ymac-1 ≈ HCC	[5, 6]
DDR1	Ddr1	Discoidin domain receptor tyrosine kinase 1	Non-detectable	Ymac-1 >> HCC	[7]
SIRT1	Sirt1	Sirtuin 1	Ddetectable	Ymac-1 ≈ HCC	[8]
PPARG	Pparg	Peroxisome proliferator activated receptor gamma	Ddetectable	Ymac-1 ≈ HCC	[9]
HMGB2	Hmgb2	High mobility group box 2	Ddetectable	Ymac-1 >> HCC	[10]
NME2	Nme2	NME/NM23 nucleoside diphosphate kinase 2 (NM23-H2)	Ddetectable	Ymac-1 > HCC	[11]
MOK	Mok	MOK protein kinase (RAGE)	Ddetectable	Ymac-1 >> HCC	[12]
LCN2	Lcn2	Lipocalin 2	Non-detectable	Ymac-1 << HCC	[13]
PTTG1	Pttg1	Pituitary tumor-transforming 1	Ddetectable	Ymac-1 ≈ HCC	[14]
EPHA2	Epha2	EPH receptor A2	Ddetectable	Ymac-1 >> HCC	[15]
DUSP1	Dusp1	Dual specificity phosphatase 1	Non-detectable	Ymac-1 ≈ HCC	[16]
VRK1	Vrk1	Vaccinia related kinase 1	Ddetectable	Ymac-1 >> HCC	[17]
RPL36A	Mrpl44	Ribosomal protein L36a	Ddetectable	Ymac-1 > HCC	[18]

aThe expression in Ymac-1 is showed as comparison of Ymac-1 and Gnmt KO HCC tissue (HCC).

b">>" or "<<" represent a difference in expression is >3-folds; ">" or "<" represent a difference in expression is >1.5-folds but <3-folds; "≈" represent a different in expression is <1.5-folds.

Supplementary References

1. Yen, C.H., et al., *Functional characterization of glycine N-methyltransferase and its interactive protein DEPDC6/DEPTOR in hepatocellular carcinoma.* Molecular Medicine, 2012. **18**(1): p. 286-296.
2. Liu, H.H., et al., *Characterization of reduced expression of glycine N-methyltransferase in cancerous hepatic tissues using two newly developed monoclonal antibodies.* Journal of Biomedical Science, 2003. **10**(1): p. 87-97.
3. Huang da, W., B.T. Sherman, and R.A. Lempicki, *Systematic and integrative analysis of large gene lists using DAVID bioinformatics resources.* Nat Protoc, 2009. **4**(1): p. 44-57.
4. Bae, J.S., et al., *Expression and role of epithelial cell adhesion molecule in dysplastic nodule and hepatocellular carcinoma.* Int J Oncol, 2012. **41**(6): p. 2150-8.
5. Kwon, C.Y., et al., *The role of serum response factor in hepatocellular carcinoma: implications for disease progression.* Int J Oncol, 2010. **37**(4): p. 837-44.
6. Bae, J.S., et al., *Serum response factor induces epithelial to mesenchymal transition with resistance to sorafenib in hepatocellular carcinoma.* Int J Oncol, 2014. **44**(1): p. 129-36.
7. Park, H.S., et al., *Overexpression of discoidin domain receptor 1 increases the migration and invasion of hepatocellular carcinoma cells in association with matrix metalloproteinase.* Oncol Rep, 2007. **18**(6): p. 1435-41.
8. Choi, H.N., et al., *Expression and role of SIRT1 in hepatocellular carcinoma.* Oncol Rep, 2011. **26**(2): p. 503-10.
9. Kim, K.R., et al., *A peroxisome proliferator-activated receptor gamma antagonist induces vimentin cleavage and inhibits invasion in high-grade hepatocellular carcinoma.* Oncol Rep, 2007. **18**(4): p. 825-32.
10. Kwon, J.H., et al., *Overexpression of high-mobility group box 2 is associated with tumor aggressiveness and prognosis of hepatocellular carcinoma.* Clin Cancer Res, 2010. **16**(22): p. 5511-21.
11. Lee, M.J., et al., *Pro-oncogenic potential of NM23-H2 in hepatocellular carcinoma.* Exp Mol Med, 2012. **44**(3): p. 214-24.
12. Cha, H.J., et al., *Overexpression of renal tumor antigen is associated with tumor invasion and poor prognosis of hepatocellular carcinoma.* Ann Surg Oncol, 2012. **19 Suppl 3**: p. S404-11.
13. Wang, Y.P., et al., *Lipocalin-2 negatively modulates the*

epithelial-to-mesenchymal transition in hepatocellular carcinoma through the epidermal growth factor (TGF-beta1)/Lcn2/Twist1 pathway. Hepatology, 2013. **58**(4): p. 1349-61.

14. Cho-Rok, J., et al., *Adenovirus-mediated transfer of siRNA against PTTG1 inhibits liver cancer cell growth in vitro and in vivo.* Hepatology, 2006. **43**(5): p. 1042-52.
15. Cui, X.D., et al., *EFNA1 ligand and its receptor EphA2: potential biomarkers for hepatocellular carcinoma.* Int J Cancer, 2010. **126**(4): p. 940-9.
16. Hao, P.P., et al., *Disruption of a regulatory loop between DUSP1 and p53 contributes to hepatocellular carcinoma development and progression.* J Hepatol, 2015. **62**(6): p. 1278-86.
17. Lee, N., et al., *Vaccinia-related kinase 1 promotes hepatocellular carcinoma by controlling the levels of cell cycle regulators associated with G1/S transition.* Oncotarget, 2015. **6**(30): p. 30130-48.
18. Kim, J.H., et al., *Over-expression of the ribosomal protein L36a gene is associated with cellular proliferation in hepatocellular carcinoma.* Hepatology, 2004. **39**(1): p. 129-38.
